# Modeling Mo(VI)=O Biologically Related Interactions with Oximes and Hydroxylamines: Implications for Uranium Seawater Extraction

**DOI:** 10.1021/acs.inorgchem.5c03731

**Published:** 2026-01-06

**Authors:** Stamatis S. Passadis, Maria Ch. Michaelidou, Wenhao Gao, Afrodite Tryfon, Angelos Kalampounias, John C. Plakatouras, Tatjana N. Parac-Vogt, Athanassios C. Tsipis, Haralampos N. Miras, Anastasios D. Keramidas, Themistoklis A. Kabanos

**Affiliations:** † Department of Chemistry, 54557 University of Cyprus, Nicosia 2109, Cyprus; ‡ Department of Chemistry, 26657 KU Leuven, Celestijnenlaan 200F, 3001 Leuven, Belgium; § School of Chemistry, 3526 The University of Glasgow, Glasgow G12 8QQ, U.K.; ∥ Department of Chemistry, 37796 University of Ioannina, Ioannina 45110, Greece

## Abstract

Molybdenum enzymes play a crucial role in the nitrogen cycle processes. However, the mechanism of Mo­(VI) reduction by hydroxylamine/oximes and its implications for oxime-based sorbents remain unclear. For decades, it has been widely accepted that the reaction of NH_2_OH with Mo­(VI) consistently results in the molybdenum reduction. This study presents evidence that challenges the prevailing view by isolating the first Mo­(VI)–oxido–hydroxylamido complex, [Mo^VI^(O)­(η^2^-NH_2_O)]^3+^, specifically [Mo^VI^(O)­(η^1^,η^1^,η^1^-pidiox-*O*,*N*,*O*′)­(η^2^-NH_2_O)­(H_2_O)], formed via hydrolysis of (2*Z*,6*Z*)-piperidine-2,6-dione dioxime (H_3_pidiox) by Mo­(VI). Τhis discovery enabled us to elucidate the long-standing mechanism of Mo­(VI) conversion to Mo^II^–NO through a combination of experimental techniques (NMR, ESI-MS, XPS, FT-IR) and density functional theory (DFT) calculations. This comprehensive approach provided new insight into molybdenum redox behavior and unambiguously confirmed the Mo­(II) oxidation state in [Mo^II^(η^1^,η^1^,η^1^-Hpidiox-*O*,*N*,*O*′)­(κ^1^-NO)­(η^2^-NH_2_O)­(OH_2_)]. In parallel, we show that H_3_pidiox, a ligand employed in uranyl extraction from seawater, undergoes hydrolysis by [Mo^VI^O_4_]^2–^ only upon formation of the complex *cis*-[Mo^VI^O_2_(pidiox)]^+^. However, this complex is hydrolytically unstable at pH 8.0, suggesting that [Mo^VI^O_4_]^2–^ is unlikely to degrade uranium oxime-based extraction materials in seawater. This study provides fundamental insight into molybdenum–oxime reactivity, offering a molecular basis for designing robust oxime-functionalized materials for efficient and durable uranium seawater extraction processes.

## Graphical abstract

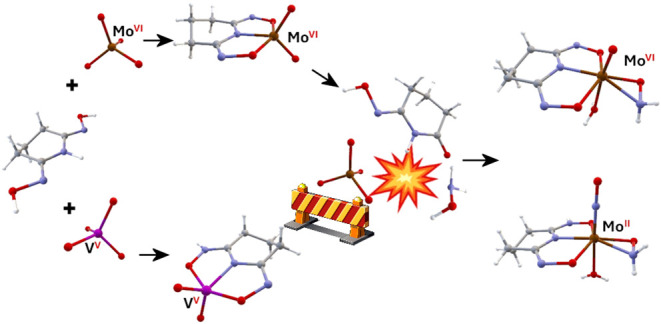

## Introduction

Molybdenum is the only second row transition metal found in several enzymes across almost all living organisms, including humans, microorganisms and plants.^1–5^ Several molybdenum enzymes also play a crucial role in the nitrogen cycle, involving species with nitrogen in all valence states, from NO_3_^–^ to NH_3_.^6^ NO, NH_2_OH and oximes are species involved in several of these reactions.^4,7–13^

The reactivity of [Mo^VI^O_4_]^2–^with oximes and NH_2_OH has been recently involved in the mining of uranyl from seawater. Polymeric sorbents functionalized with oximes have been extensively studied for this application.^14–25^ Li, and co-workers^26^ showed that [Mo^VI^O_4_]^2–^ hydrolyzes the ligand (2Z,6Z)-piperidine-2,6-dione dioxime (H_3_pidiox) (Scheme 1). This reactivity suggests that polymeric sorbents containing oxime functional groups might be susceptible to decomposition by [Mo^VI^O_4_]^2–^ in seawater. Given that the concentration of [Mo^VI^O_4_]^2–^ (107 nmol/L)^27^ in the seawater is significantly higher than that of uranyl (14 nmol/L),^28,29^ and vanadium­(V) (37 nmol L^–1^),^30^ and despite the fact that both uranyl and vanadium­(V) coordinate strongly with oximes, [Mo^VI^O_4_]^2–^ could interfere with uranium mining from seawater. Although Li and co-workers^26^ reported the hydrolysis of H_3_pidiox by [Mo^VI^O_4_]^2–^ and conversion of it to a Mo-NO complex, several studies continue to suggest the use of oxime-based materials for uranyl extraction from seawater.^31,32^

**Scheme 1:**
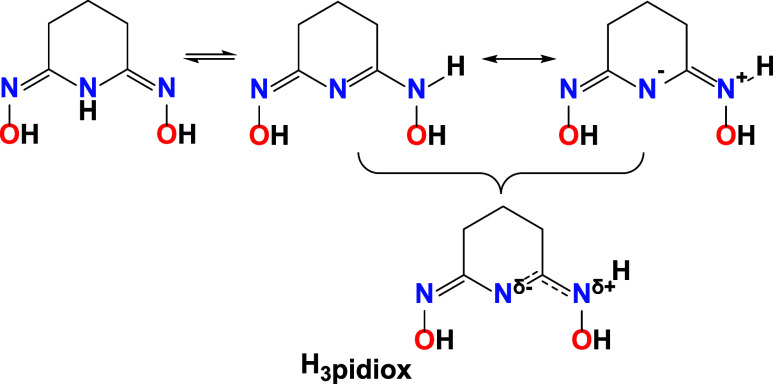
Ligand H_3_pidiox and Its Tautomeric and Resonance Structures

The transformation of Mo­(VI) to Mo–NO complexes via hydroxylamine has been primarily examined by Wieghardt et al.^33–39^ The nearly linear Mo–N–O configuration depicted in Scheme 2 can be formally represented as {MNO}^4^ within the Enemark–Feltham framework,^40^ where the superscript denotes the total count of metal d and NO π* electrons. It is important to note that the Enemark–Feltham notation is employed solely as an electron-counting convention and not for assigning formal oxidation states. An ongoing ambiguity in the literature pertains to the valence state of molybdenum in Mo–NO systems, which is variably reported as either Mo­(IV) or Mo­(II), with corresponding NO^–^ or NO^+^ states, even when the Mo–N–O unit is nearly linear.^26,34^ Ambiguity is frequently observed in transition metals coordinated with the noninnocent nitrosyl ligand, where the NO group in a linear configuration can exhibit charges +1, 0 and in few exceptions −1.^41–45^

**Scheme 2:**
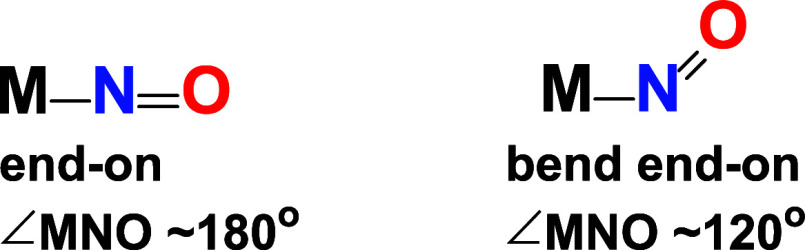
Binding Modes of an *N*-Bound Nitrosyl Ligand at Transition Metal Site

The Mo–NO fragment exhibits significant covalency, with M–N–O angles ranging from 120° to 180*°*;^46–50^ the actual electronic structure is subsequently assessed using DFT (vide infra). Metal nitrosyls with bent M–N–O angles (120°–140°) are typically associated with more reduced NO ligands (Scheme 2). The ν­(NO) values for linear M–N–O units generally range from 1450 to 1950 cm^–1^, whereas bent geometries often exhibit ν­(NO) between 1400 and 1720 cm^–1^.^49,51^

Although Mo–NO chemistry has been examined for many years, the series of redox and ligand-exchange events that take Mo­(VI) precursors into Mo–NO products have not been fully clarified. Li and co-workers^26^ proposed a mechanism; however, it was based mainly on some assumptions and thus, the mechanism of Mo­(VI) reduction by hydroxylamine and its implications for oxime-based sorbents remain unclear.

Herein, we present a systematic study of the redox and coordination steps that lead from the Mo­(VI) starting complex to the formation of the Mo–NO species, based on experimental data and DFT calculations. Specifically, a unique Mo­(VI)-oxido-hydroxylamido complex, bearing a H_3_pidiox ligand, [Mo^VI^(O)­(η^1^,η^1^,η^1^-pidiox-*O*,*N*,*O*′)­(η^2^-NH_2_O)­(H_2_O)], **1** (Scheme 3); was synthesized and characterized physico-chemically and structurally. This synthesis contrasts sharply with the prevailing assumption that molybdenum­(VI) cannot coexist with NH_2_OH which reduces it. With the mechanism of Mo­(VI) to Mo-NO conversion established, we were able to synthesize [Mo^II^(κ^1^-NO)­(η^1^,η^1^,η^1^-Hpidiox-*O*,*N*,*O*′)­(η^2^-NH_2_O)­(H_2_O)] (**3**) (Scheme 3) rapidly and in a high yield. The oxidation state of molybdenum was ascertained through experimental data and density functional theory (DFT) calculations. Additionally, competition studies involving Mo­(VI)–V­(V)/H_3_pidiox were conducted utilizing ^1^H and ^51^V NMR spectroscopy to evaluate the hydrolysis of H_3_pidiox by Mo­(VI) and determine its efficacy as a chelating agent for uranium extraction from seawater. V­(V) was selected as the competing metal ion for Mo­(VI) due to the fact that oximes exhibit stronger ligation for V­(V) among the metal ions present in seawater. Moreover, the vanadium nucleus is NMR-active, providing an additional method to investigate the hydrolysis of oximes from [Mo^VI^O_4_]^2–^.

**Scheme 3:**
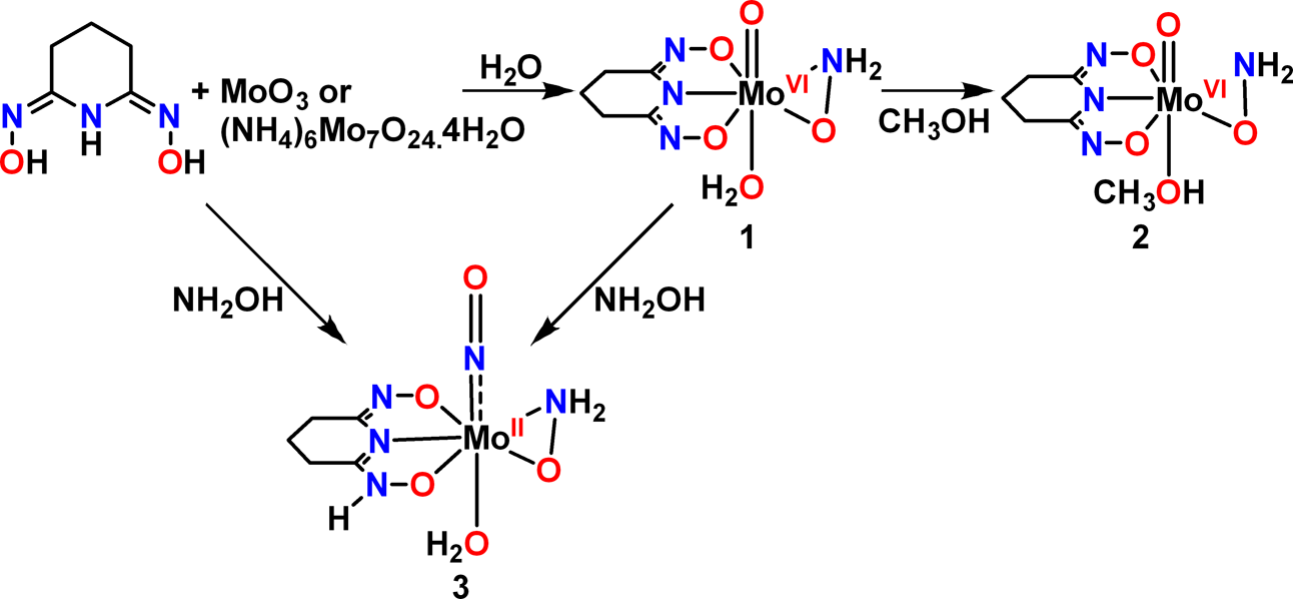
Synthesis of the Complexes **1**, **2**, and **3**

## Experimental Section

All chemicals and solvents were purchased from Sigma-Aldrich and Merck, were of reagent grade, and were used without further purification. C, H, and N analyses were conducted by the microanalytical service of the School of Chemistry, University of Glasgow. The ligand H_3_pidiox was synthesized according to the literature.^52^ *No uncommon hazards are noted*.

### Synthesis of [Mo^VI^(O)­(η^1^,η^1^,η^1^-pidiox-*O*,*N*,*O*′)­(η^2^-NH_2_O)­(H_2_O)] (**1**)

#### Method A

MoO_3_ (0.0500 g, 0.347 mmol) and H_3_pidiox (0.0994 g, 0.694 mmol) were successively added to a stirred aqueous solution (3 mL). The resulting suspension was stirred for 2 days, during which an orange precipitate gradually formed, and the supernatant solution also became orange. The precipitate was collected by filtration (pH of the filtrate 5.5) and washed with cold methanol (2 × 3 mL) and diethyl ether (2 × 5 mL) and dried in air for 3 days to get 0.0835 g of **1**. Yield: (80%, based on MoO_3_). Anal. Calc. for C_5_H_10_N_4_O_5_Mo (*M*_r_ = 302.12), (found values in parentheses): C, 19.88% (19.93%); H, 3.34% (3.30%); N, 18.55% (18.45%). Orange crystals of [Mo^VI^(=O)­(η^1^,η^1^,η^1^-pidiox-*O*,*N*,*O*′)­(η^2^-NH_2_O)­(H_2_O)]·2CH_3_OH, **1**·2CH_3_OH and [Mo^VI^(=O)­(η^1^,η^1^,η^1^-pidiox-*O*,*N*,*O*′)­(η^2^-NH_2_O)­(CH_3_OH)]·2CH_3_OH, **2**·2CH_3_OH suitable for single-crystal X-ray diffraction study were obtained by dissolving **1** in methanol, filtering the solution, and allowing it to evaporate slowly at room temperature (20 °C) for 5 days. The bound water molecule in **1** was not completely replaced by methanol when **1** was dissolved in methanol to get crystals and thus, the crystals which were formed were a mixture of **1**·2CH_3_OH and **2**·2CH_3_OH in approximately 70/30% w/w on the basis of CHN analyses. The color of both crystals was orange, and their shape was very similar and thus, we were unable to separate them even under a microscope.

#### Method B

To a stirred aqueous solution (3 mL) were successively added H_3_pidiox (0.0480 g, 0.336 mmol), and (NH_4_)_6_Mo_7_^VI^O_24_·4H_2_O (0.0600 g, 0.048 mmol). Upon addition of the molybdenum salt the solution cleared after 10 min of stirring and its color became orange. The solution stirred overnight, and an orange precipitate was formed, which was filtered off (pH of the filtrate 5.5), washed with cold CH_3_OH (2 × 2 mL), (C_2_H_5_)_2_O (2 × 5 mL) and air-dried for 3 days to obtain 0.0207 g of an orange solid. Yield: 20% [based on (NH_4_)_6_Mo_7_O_24_·4H_2_O]. The identity of the orange material was confirmed by IR and UV–vis (in water) spectroscopic comparison with compound **1** and CHN elemental analyses.

### Synthesis of [Mo^II^(η^1^ *,η* ^1^ *,η* ^1^-Hpidiox-*O*,*N*,*O*′)­(κ^1^-NO)­(η^2^-NH_2_O)­(OH_2_)]·2H_2_O, 3·2H_2_O

#### Method A

To a stirred aqueous solution (1 mL), were successively added H_3_pidiox (0.0199 g, 0.139 mmol) and 17 μL of NH_2_OH (50% in water) (0.278 mmol) under nitrogen. Then, MoO_3_ (0.0200 g, 0.139 mmol) was added, and the colorless solution turned orange. The resulting suspension was stirred for 60 min and then filtered. The filtrate was allowed to evaporate at room temperature (20 °C). After 2 days the color of the solution turned yellow and X-ray quality pale-yellow crystals were formed, which were collected by filtration (pH of filtrate 5.5), and dried in air for 3 days tο get 15 mg of **3**.2H_2_O. Yield: 30% (based on MoO_3_). Anal. Calc. (exp.) for C_5_H_15_N_5_O_7_Mo (*M*_r_ = 353.16): C, 17.00% (17.09%); H, 4.28% (4.24%); N, 19.83% (20.01%).

#### Method B

To a stirred aqueous solution (1 mL), were successively added H_3_pidiox (0.0243 g, 0.169 mmol) and 20.8 μL of NH_2_OH (50% in water) (0.339 mmol) under a nitrogen atmosphere. Then, (NH_4_)_6_Mo_7_O_24_·4H_2_O (0.03 g, 0.024 mmol) was added, and the colorless solution turned orange. After 1 h of stirring, the solution turned yellow, and a yellow precipitate began to form. The solution was stirred overnight and then, it was filtered off (pH of filtrate 5.5) to obtain 43 mg of a yellow solid. Yield: 80% (based on (NH_4_)_6_Mo_7_O_24_·4H_2_O). The identity of the yellow solid was confirmed by IR and UV–vis (in water) spectroscopic comparison with the crystals of method A and CHN elemental analyses.

#### Method C

To a stirred aqueous solution (3 mL) were successively added H_3_pidiox (0.096 g, 0.670 mmol) and (NH_4_)_6_Mo_7_O_24_·4H_2_O (0.06 g, 0.048 mmol). Upon addition of (NH_4_)_6_Mo_7_O_24_·4H_2_O the solution cleared, and its color turned orange. The orange solution was stirred for 10 min, and then it was filtered. The filtrate was left to evaporate slowly at room temperature (20 °C). After standing for 3 days, the solution turned yellow, and after ten further days, pale-yellow crystals of **3**·2H_2_O suitable for single-crystal X-ray diffraction analysis had formed. The crystals were collected by filtration, and then air-dried for 3 days. Overall yield 29 mg [27% based on (NH_4_)_6_Mo_7_O_24_·4H_2_O]. The identity of the crystalline material was confirmed by single crystal X-ray structure analysis, IR spectroscopic comparison with the crystals of method A and CHN elemental analyses.

#### Method D

To a stirred aqueous solution (1 mL) were successively added H_3_pidiox (0.0148 g, 0.103 mmol) and Na_2_Mo^VI^O_4_·2H_2_O (0.025 g, 0.103 mmol). The mixture was stirred for 15 min, followed by 12.6 μL of NH_2_OH (50% in water) (0.206 mmol). After stirring for 3 h, the solution turned yellow and became clear (pH = 11.1). The pH was adjusted to 7.3 using 0.1 M HCl. Following overnight stirring, a pale yellow precipitate was formed, collected by filtration, washed with cold CH_3_OH (2 × 3 mL), (C_2_H_5_)_2_O (2 × 3 mL) and dried in air. Overall yield 6.3 mg [19% based on Na_2_Mo^VI^O_4_·2H_2_O]. The identity of the precipitate was confirmed by IR spectroscopic comparison with crystals of method A and CHN elemental analyses.

#### Method E

Complex **1** (0.0169 g, 0.055 mmol) was added to water (1.5 mL) and warmed to 40 °C, giving a clear orange solution. The solution was then cooled to room temperature, and 6.66 μL NH_2_OH (50% in water, 0.11 mmol) was added. The solution was stirred overnight, a pale yellow precipitate was formed, collected by filtration (pH of filtrate 7.0), washed with cold CH_3_OH (2 × 3 mL), (C_2_H_5_)_2_O (2 × 3 mL) and dried in air. Yield: 14.82 mg (85%, based on compound **1**). The identity of the precipitate was confirmed by IR spectroscopic comparison with crystals of method A and CHN elemental analyses.

## Results and Discussion

### Synthesis

To explore the effects of varying experimental conditions and their potential impact on the outcome of the chemical system under investigation, we employed the following synthetic protocols. Complex **1** was synthesized according to Scheme 3 using either MoO_3_ (method A), or (NH_4_)_6_Mo_7_O_24_.4H_2_O (method B) as a molybdenum­(VI) source and the ligand H_3_pidiox. Complex **3**·2H_2_O was synthesized using MoO_3_ (method A, Scheme 3), (NH_4_)_6_Mo_7_O_24_·4H_2_O (method B, Scheme 3) and Na_2_Mo^VI^O_4_·2H_2_O (method D) as a source of molybdenum, the ligand H_3_pidiox and NH_2_OH. In aqueous solution, the ligand H_3_pidiox undergoes hydrolysis in the presence of molybdenum­(VI) to yield (*Z*)-6-(hydroxyimino)­piperidin-2-one (Hhypo), piperidine-2,6-dione (pidion), and hydroxylamine, as shown in Scheme 4.^26^ Thus, the hydroxylamine present in the synthesis of compounds **1** (methods A and B) and **3**·2H_2_O (method C) is generated by the hydrolysis of H_3_pidiox, which therefore plays a dual role both as a ligand and an internal source of hydroxylamine.

**Scheme 4:**
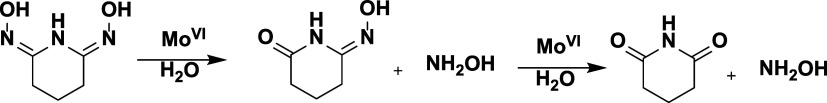
Hydrolysis of the Ligand H_3_pidiox in the Presence of Mo­(VI)

The compound **3**·2H_2_O was also synthesized by reacting H_3_pidiox with (NH_4_)_6_Mo_7_O_24_·4H_2_O according to the literature (method C).^26^ Moreover, compound **3**·2H_2_O was prepared by reacting **1** with NH_2_OH (method E).

At this point, it is worth noting that the synthesis of compound **3**·2H_2_O takes place spontaneously at room temperature (20 °C) with or without the presence of NH_2_OH (in this case hydroxylamine is produced *in situ* due to the hydrolysis of the ligand H_3_pidiox). In marked contrast, almost all the molybdenum­(II) compounds reported in the literature, containing the structural unit [Mo^II^(κ^1^ **-**NO)­(η^2^-H_2_NO)], were prepared in the presence of huge excess of hydroxylamine under elevated temperature regimes.^33–39,53^

### Characterization of the Compounds **1**·2CH_3_OH, **2**·2CH_3_OH, and **3**·2H_2_O

#### X-ray Crystallographic Results

Crystal data and structure refinement details for the compounds **1**·2CH_3_OH, **2**·2CH_3_OH, and **3**·2H_2_O are given in Table S1.

Compounds **1**·2CH_3_OH and **2**·2CH_3_OH exhibit very similar molecular structures (Figures 1 and S1), though crystallized in different space groups; the former crystallized in triclinic *P*1̅ and the latter in monoclinic *P*2_1_/*n.* Selected geometrical characteristics relevant to the coordination sphere of molybdenum for **1**·2CH_3_OH and **2**·2CH_3_OH are presented in Table S2.

**Figure 1:**
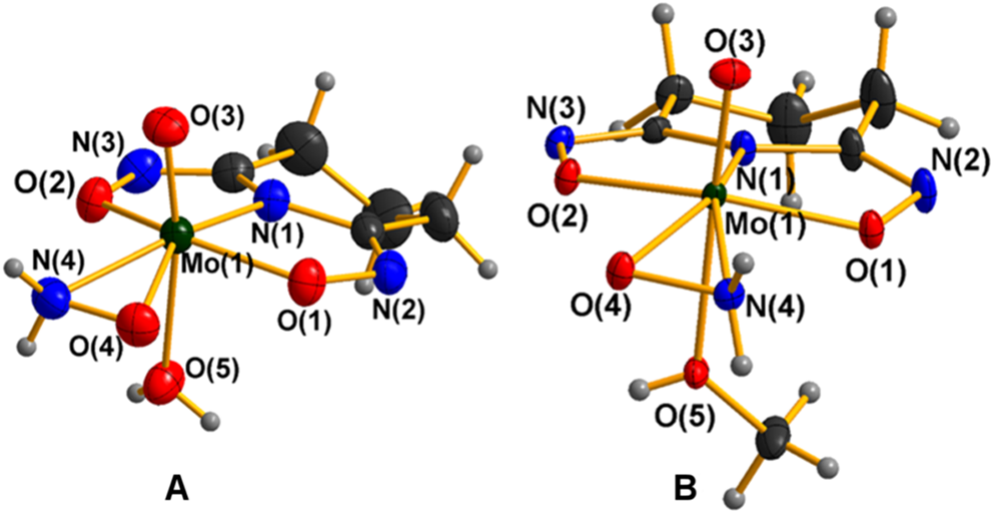
X-ray crystal structures of the compounds **1**·2CH_3_OH (A) and **2**·2CH_3_OH (B). Any cocrystallized solvent molecules were omitted for clarity. Ellipsoids were set at 50%. Color code: Mo, green; O, red; N, blue; C, black; H, gray.

The molybdenum atom in **1**·2CH_3_OH (Figure 1A) adopts a highly distorted N_2_O_5_ pentagonal bipyramidal coordination environment and is bonded to a triply deprotonated ligand pidiox^3–^ through the cyclic nitrogen atom N(1), and the two oxime oxygen atoms O(1) and O(2), forming two fused five-membered chelate rings, a bidentate hydroxylamido-*O*,*N* ligand in the equatorial plane and an oxido group with a water oxygen atom in the axial positions (Figure 1A).

The Mo­(VI)–N_amido_ [Mo(1)–N(1)] bond distance of 2.016 Å (mean value for **1**·2CH_3_OH and **2**·2CH_3_OH), is well within the range of other Mo­(VI)–N_amido_ complexes.^54–57^ The majority of oximato Mo­(VI) complexes contain either chelated or bridging =N–O^–^ group.^58^ The Mo­(VI)–O_oximate_ and the Mo­(VI)–O_water/methanol_ bond distances [Mo(1)–O(5)_H2O/CH3OH_, 2.285(3) and 2.323(3) Å for H_2_O and CH_3_OH respectively] fall within the range of other examples reported previously in the literature.^59–64^

The distortion of the coordination polyhedron in **1**·2CH_3_OH is being induced by the small bite angle of the three-membered hydroxylamido-*O*,*N* ring. The equatorial pentagon is much distorted (the distances between successive donors span the range 1.392–2.822 Å), while the largest deviation from the calculated least-squares plane is 0.015 Å. The molybdenum­(VI) atom sits 0.39 Å out of the equatorial N_4_O plane, toward the oxido group [O(3)]. The Mo=O bond distance [Mo(1)–O(3), 1.683(3) Å] is in good agreement with previously reported Mo­(VI) complexes.^65–67^

To the best of our knowledge, **1**·2CH_3_OH and **2**·2CH_3_OH are the first two examples of coordination compounds between a hydroxylamido-*N*,*O* ligand and a molybdenum­(VI) atom. Among the very few molybdenum–hydroxylamido compounds reported in the CSD, the molybdenum center is in the oxidation state II, while the hydroxylamido ligand is typically coordinated alongside a nitrosyl cation.^53,68–70^ The XPS spectra (Figure S2) derived from the surface included a molybdenum 3d_5/2_ spectrum of 232.1 eV binding energies for complex **1** and 230.3 eV for complex **3**·2H_2_O, which are in good agreement with previously reported Mo­(VI) and Mo­(II) complexes, respectively.^71,72^ This finding is in contrast to what has been reported by Li and co-workers,^26^ claiming that the oxidation state of molybdenum in complex **3**·2H_2_O is IV.

Compound **3**·2H_2_O exhibits also a 7-coordinate molybdenum complex with a distorted pentagonal bipyramidal coordination sphere around the molybdenum atom (Figures 2 and S3), yet it differs significantly from **1**·2CH_3_OH, and **2**·2CH_3_OH. Selected geometrical characteristics of **3**·2H_2_O are presented in Table S3.

**Figure 2:**
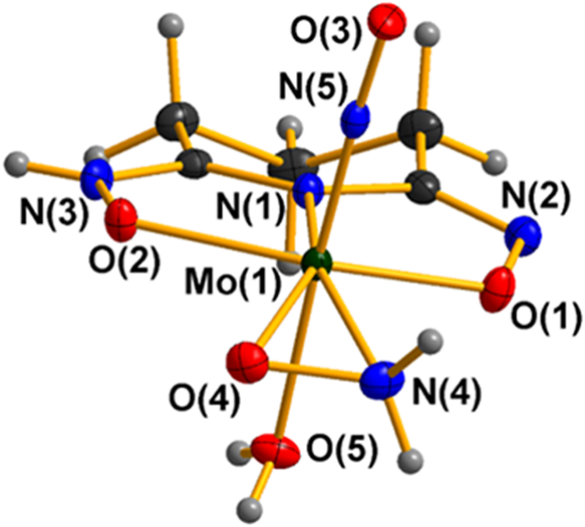
X-ray crystal structure of the compound **3**·2H_2_O. Any cocrystallized solvent molecules were omitted for clarity. Ellipsoids were set at 50%. Color code: Mo, green; O, red; N, blue; C, black; H, gray.

The Mo­(II) atom in **3**·2H_2_O is positioned 0.23 Å above the equatorial plane and toward the NO ligand. The ligand NO behaves as a π-acceptor and the metal–ligand π-bonds arise from the *back-donation* of electrons from the metal center to the vacant antibonding orbitals of the ligand. The NO as a π-acceptor ligand can induce stability of low oxidation states of transition metal centers^73^ and the terminally *N*-bound NO can adopt two different bonding modes: linear or bent (Scheme 2).

The pair of complexes **1**·2CH_3_OH and **3**·2H_2_O constitutes a unique example of Mo^VI^(=O) and Mo^II^(NO) compounds respectively with the same N_4_O_2_ coordination environment. Table 1 presents a comparison of the corresponding bond distances of **1**·2CH_3_OH and **3**.2H_2_O. The Mo­(II)–donor atom distances are longer (0.06–0.10 Å) than the corresponding Mo­(VI) distances, due to the difference in the oxidation state of Mo (VI–II), except the d­(Mo–N_H2NO‑_) which is the same for both compounds and d­(Mo–O_H2O_) which is longer by 0.07 Å as expected in **1**·2CH_3_OH due to the stronger trans influence of the oxido ligand.

**Table 1:** Comparison of Selected Bond Distances (Å) of the Complexes **1**·2CH_3_OH and **3**·2H_2_O

	**1**·2CH_3_OH	**3**·2H_2_O
Mo(1)–N(1)	2.015(3)	2.081(2)
Mo(1)–O(1)	1.974(3)	2.068(2)
Mo(1)–O(2)	2.005(3)	2.105(2)
Mo(1)–N(4)	2.084(4)	2.086(2)
Mo(1)–O(4)	1.984(3)	2.048(2)
Mo(1)–O(5)	2.285(3)	2.214(2)
Mo(1)–O(3)	1.683(3)	
Mo(1)–N(5)		1.748(2)
N(1)–C(1)	1.377(5)	1.330(3)
N(1)–C(5)	1.383(5)	1.382(3)

In addition to crystallographic analysis, infrared (IR) spectroscopy offers a valuable insight into the bonding interactions between the metal ion and the NO^+^ in the solid state.^51^ Figure S4 presents the Fourier-transform infrared (FT-IR) transmittance spectra of complexes **1** and **3**·2H_2_O, recorded under ambient conditions in the solid state, with detailed assignments discussed in theSupporting Information (SI). A comparative analysis of the IR spectra of **1** and **3**·2H_2_O in the selected spectral region, 800–1700 cm^–1^ (Figure 3), alongside density functional theory (DFT) calculations, reveals that the peaks at 1597 cm^–1^ for **3**·2H_2_O and 948 cm^–1^ for **1** correspond to the ν­(N=O) and ν­(Mo=O) stretching vibrations, respectively. The low frequency of the N=O bond stretching vibration indicates a strong π-back bonding from Mo­(II) to NO, attributed to the strong electron-donating properties of the other ligands, which provide the metal with sufficient electron density for the formation of a strong Mo=NO π-bond, thereby weakening the N=O bond.

**Figure 3:**
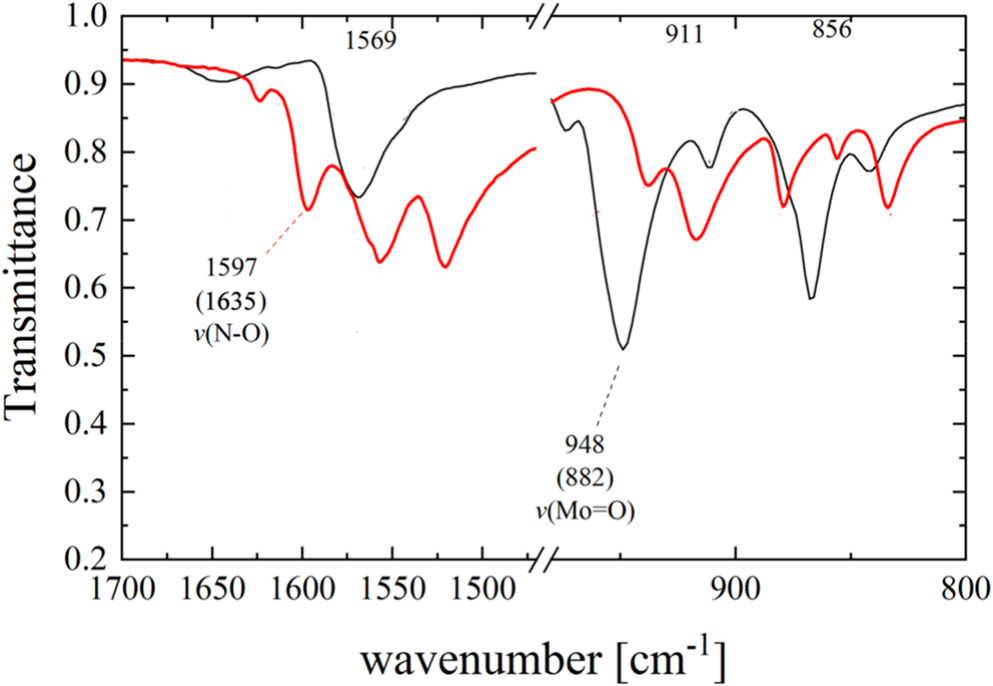
IR spectra of the complexes 1 (black color) and 3.2H_2_O (red color) (800–1700 cm^–1^). The numbers in parentheses indicate the corresponding theoretically predicted vibrational frequencies.

#### DFT Calculations of Bonding and Electronic Properties of **3**

The calculated Mo–N–O bond angle is 178.2°, at the PBE0/def2-TZVP level, falling within the range 165–180° typical for nitrosyl complexes bearing the NO^+^ cation ligand. The symmetric stretching vibrational frequency, v_s_(N–O) of the N–O bond in complex **3** is calculated to be 1699 cm^–1^, and the corresponding bond length is 1.197 Å at the same level of theory. These values suggest the presence of metal-to-ligand π-back-bonding from the Mo center to the coordinated nitrosyl ligand. Notice that, the v_s_(N–O) calculated for **3**, falls within the range of strongly π-accepting NO^+^, being linearly coordinated to metal centers. On the other hand, the Wiberg Bond Index (WBI) calculated for the N–O bond is 1.526 and taken together with the computed N–O bond length imply a classical nitrosonium-like NO^+^ ligand with a substantial reduction in bond order compared to the free NO^+^ cation, whose WBI is 2.805 at the same level of theory, consistent with its formal triple-bond description. In order to clarify the electronic distribution within the Mo–NO framework and assess the Mo oxidation state/d electron count and the redox state of the NO ligand character (NO^+^/NO^•0^/NO^–^), we performed a wave function stability analysis calculation. Accordingly, we found that the closed-shell singlet solution is fully stable with respect to spin- or symmetry-breaking. No lower energy broken-symmetry solution could be located. Thus, there is no indication of a Mo-centered spin antiferromagnetically coupled to an NO-centered unpaired electron, which rules out significant NO^•^ character. Taken together with the relatively high ν­(N–O), the moderate N–O bond elongation, and the absence of any substantial π-occupation characteristic of NO^–^, this also argues against a dominant NO^–^-like formulation. Overall, the data support a closed-shell {MNO}^4^ unit best described by a Mo­(II)–NO^+^-leaning resonance picture. In addition, the *d* electron count, as extracted from the NBO analysis, is 4.4 e^–^ which is much closer and consistent with the d^4^ Mo­(II) oxidation state rather than to d^6^ Mo(0), d^2^ Mo­(IV) or d^0^ Mo­(VI) oxidation states. Formally, we could then describe complex **3** as a {MNO}^4^ system in terms of the Enemark–Feltham scheme. We note that both Mo­(II)–NO^+^ and Mo­(IV)–NO^–^ formal assignments lead to the same Enemark–Feltham superscript *n* = 4, so the {MNO}^4^ notation does not distinguish between these limiting resonance descriptions. The computed *d*-electron count, π* occupation, and closed-shell configuration, however, clearly favor the Mo­(II)–NO^+^ resonance form. Upon coordination, the N–O bond is best described as intermediate between a single and double bond, consistent with partial back-donation from the Mo­(II) center and partial population of the π* orbitals of the NO ligand. It is important to note that in coordinated nitrosyl complexes parameters such as N–O bond length and Wiberg bond index cannot be interpreted in the same way as for isolated NO^+^/NO/NO^–^ species. Coordination intrinsically alters the π-system of the ligand, and even formally NO^+^ complexes often show substantial N–O weakening due to metal → NO π-backbonding. Thus, the observed N–O elongation and reduced bond order in this system are fully compatible with a {MNO}^4^ unit described by the Mo­(II)–NO^+^ resonance form and do not imply formal ligand reduction. The back-donation from the Mo­(II) center toward the NO ligand is also illustrated by a donor – acceptor interaction derived from NBO analysis. In Figure S5 are depicted the donor NBO, LP­(Mo), (lone pair being almost purely of Mo *d* character) as well as an acceptor NBO, BD*­(N–O) (antibonding NBO of π-type character located on NO ligand). The LP­(Mo) → BD*­(N–O) hyper-conjugative back-donation interaction reflects the strong π-back-donation from Mo to the NO ligand. The stabilization energy, Δ*E*(2), associated with the charge transfer (CT) interactions between these donor–acceptor orbitals, computed from the second-order perturbative estimates of the Fock matrix in the NBO analysis, is 186 kcal/mol and thus π-back-donation significantly stabilizes the system. Next, we set out to delineate the nature of the Mo–NO bond in **3** using the NBO method. In Figure S5 are depicted two NBOs relevant to the Mo–NO bond namely the BD1­(Mo–NO) and BD2­(Mo–NO). The former is constructed by the linear combination of a sd^4.65^ Mo hybrid (17.7% *s* and 82.2% d character) with sp^0.59^ hybrid (63% s and 37% p character) of NO ligand while the latter is a linear combination of Mo d AO with an N p AO. The BD1­(Mo-NO) has an occupation number of 1.983 and is described as σ­(Mο–NO) = 0.448h_Mo_ + 0.894h_N_. On the other hand, the BD2­(Mo-NO) has an occupation number of 1.936 and is described as σ­(Mο–NO) = 0.684h_Mo_ + 0.729h_N_. It should be emphasized that the idealized partitioning, IUPAC oxidation-state concept relying on a purely ionic approximation in which electrons are assigned according to electronegativity differences, cannot be applied for highly covalent metal–nitrosyl in any straightforward or chemically reliable way. The Mo–NO bond is dominated by a combination of σ-donation, π-back-bonding, and several contributing resonance forms, so forcing an ionic electron count inevitably collapses the actual bonding situation into an unrealistic and ambiguous picture. In contrast, the NBO analysis used here explicitly resolves the covalent σ and π components of the Mo–NO interaction and therefore provides a much more chemically meaningful description of the electron distribution in this system. For this reason, the Enemark–Feltham {MoNO}^4^ formulation, supported by both our experimental data and computed reaction profile, remains the appropriate framework, with the Mo­(II)–NO^+^ resonance form emerging as the dominant and chemically consistent description.

In Figure S6 are depicted also the MOs relevant to Mo–NO bond in **3**. Starting with the HOMO, we observe that it is bonding at the Mo–NO bond region, arising from the in-phase, side-on overlap of d_*xz*_ AO of Mo with the π* orbital of the NO ligand. The same holds also for a number of lower lying MOs i.e., HOMO–1, HOMO–2 and HOMO–4 which are also bonding combinations of d AOs (d_*yz*_/d_*xz*_) of Mo with the π* orbital of the NO ligand. There are also two other, low-lying MOs contributing to the Mo–NO bonding interaction, namely the HOMO–21 and HOMO–22. These MOs are bonding combinations of Mo d AOs with the π orbital of the NO ligand. Also, there are another two, low-lying MOs i.e., HOMO–24 and HOMO–26 which are bonding combinations of Mo d_z2_ AO with p AO located on the N and O atoms of the NO ligand.

#### UV–vis Spectroscopy

The UV–vis spectra of aqueous solutions of H_3_pidiox, complexes **1**, and **3**·2H_2_O are presented in Figure 4, and the corresponding spectroscopic data are summarized in Table 2. All three compounds exhibit a strong absorption band in the range of 225–235 nm, which is tentatively attributed to intraligand transitions of the H*_n_* pidiox^(*n*–3)–^ ligand. The peaks located at 290, and 422 for **1** are assigned to H*_n_* pidiox^(*n*–3)–^ → Mo­(VI) LMCT transitions. In a similar manner the peaks located at 280 and 326 of **3**·2H_2_O nm are originated from H*_n_* pidiox^(*n*–3)–^ → Mo­(II) and ON^+^→ Mo­(II) LMCT transitions. The calculated absorption spectra show excellent agreement with those obtained experimentally (Figures S7–S10 and Table S5).

**Figure 4:**
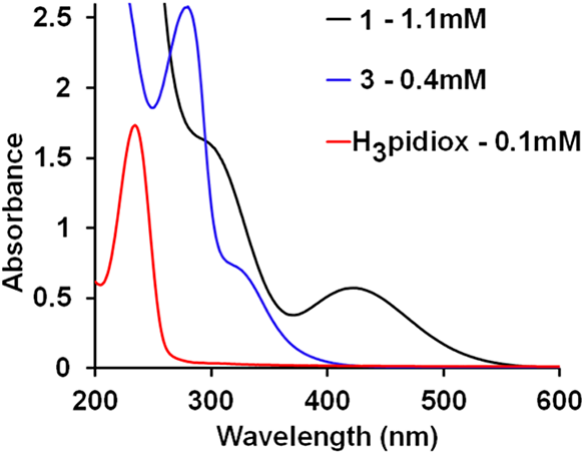
UV–vis spectra of the aqueous solution of **1** (1.1 mM), **3**·2H_2_O (0.4 mM), and the ligand H_3_pidiox (0.1 mM).

**Table 2:** UV-Vis Data in H_2_O for the Complexes **1**, **3**·2H_2_O and the Ligand H_3_pidiox

compound	λ (nm)	ε (M^–1^ cm^–1^)	λ (nm)	ε (M^–1^ cm^–1^)	λ (nm)	ε (M^–1^ cm^–1^)
**1**	225 (sh)	12.3 × 10^3^	290	1.4 × 10^3^ (sh)	422	0.5 × 10^3^
**3**·2H_2_O	-	-	280	7.3 × 10^3^	326 (sh)	1.7 × 10^3^
**H**_**3**_ **pidiox**	235	15.7 × 10^3^	–		–	–

#### NMR Spectroscopy

The NMR measurements were acquired in D_2_O solutions. The ^1^H NMR spectra of the ligand H_3_pidiox and complexes **1** (3.3 mM, pD = 5.8) and **3**·2H_2_O (3.0 mM, pD = 5.8) are shown in Figure 5 and the NMR data are summarized in Table 3. The ^1^H NMR spectrum of the crystals, obtained from the dissolution/evaporation of compound **1** in CH_3_OH, was the same with the spectrum of **1**. The only difference was a single peak at 3.2 ppm attributed to free CH_3_OH and the integrals of the peaks reveal a small amount (<30%) of CH_3_OH in the crystals in accordance with the elemental analysis. The ^1^H NMR spectrum of H_3_pidiox gave a triplet at 2.64 ppm assigned to Ha and a quintet at 1.75 ppm assigned to Hb protons (*J*_HaHb_ = 6.9 Hz, Figure 5). The ligation of pidiox^3–^ to Mo­(VI) in **1** results in a shift of the peaks to lower field and differentiation of the geminal protons Ha and Hb, in accordance with the symmetry of the complex in its crystallographically defined structure (Figures 1A and 5). This causes a further splitting of the peaks, including a large splitting between the geminal protons (*J*_HbHb′_ = 55 Hz, Figure 5). The reduced symmetry of **3**.2H_2_O compared to **1** (Figure 5) leads to the differentiation of the four Ha,a′ protons into two Ha,a′ and two Hc,c′ in the ^1^H NMR spectrum of **3**.2H2O. The ^1^H NMR spectra of the complexes **1** and **3**·2H_2_O confirm further their structural integrity in solution.

**Figure 5:**
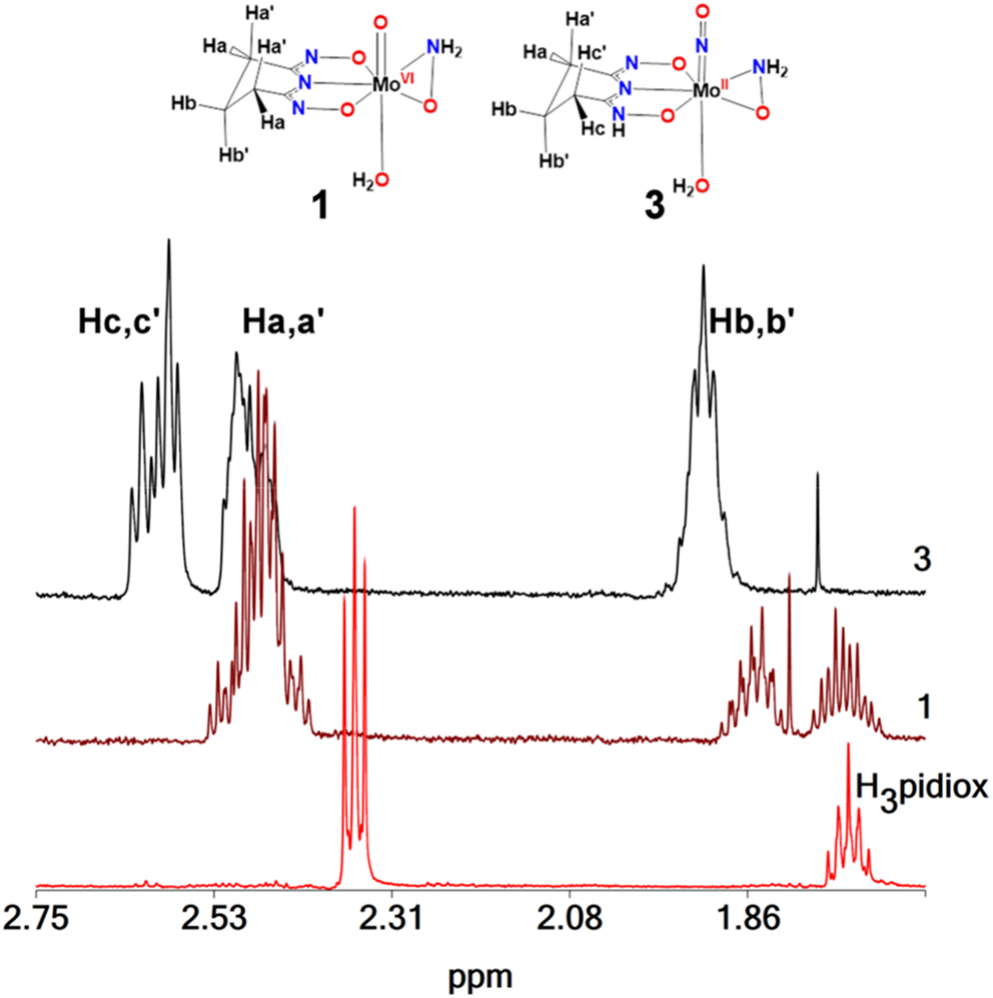
^1^H NMR spectra of H_3_pidiox (3.0 mM), **1** (3.3 mM), and **3**·2H_2_O (3.0 mM) at pD = 5.8. Labeling of the protons of the ligand pidiox^3–^ in **1** and Hpidiox^2–^ in **3**.2H_2_O.

**Table 3:** ^13^C­(^1^H) Chemical Shifts (ppm) ^1^H and ^13^C (Obtained From 2D {^1^H,^13^C} HSQC and HMBC NMR) of the Ligand H_3_pidiox and of the Complexes **1** and **3**·2H_2_O

comp.	C=N–	C=NH–	C_c_(H_c_)	C_a_(H_a_)	C_b_(H_b_)
**H**_**3**_ **pidiox**	156.40			20.16 (2.37t) (*J*_HaHb_ = 6.3 Hz)	19.64 (1.75p)
**1**	177.12			30.52 (2.46m)	19.89 (1.80m) (*J*_HbHb′_ = 55 Hz)
**3**·2H_2_O	155.48	156.46	20.57 (2.58) (*J*_HcHc′_ = 44, *J*_HcHb_ = 7.4, *J*_Hc′Hb′_ = 5.0 Hz)	20.57 (2.61) (*J*_HaHa′_ = 54, *J*_HaHb_ = 6.9, *J*_Ha′Hb′_ = 5.0 Hz)	18.14 (1.96m)

### Investigation of the Reactivity of [Mo^VI^O_4_]^2–^ with H_3_pidiox by NMR and ESI-MS Studies

#### Hydrolysis of the Ligand H_3_pidiox by Mo­(VI)

The hydrolysis of the ligand H_3_pidiox when it reacts with Mo­(VI) in D_2_O vs time was investigated by ^1^H NMR spectroscopy (Figure 6).

**Figure 6:**
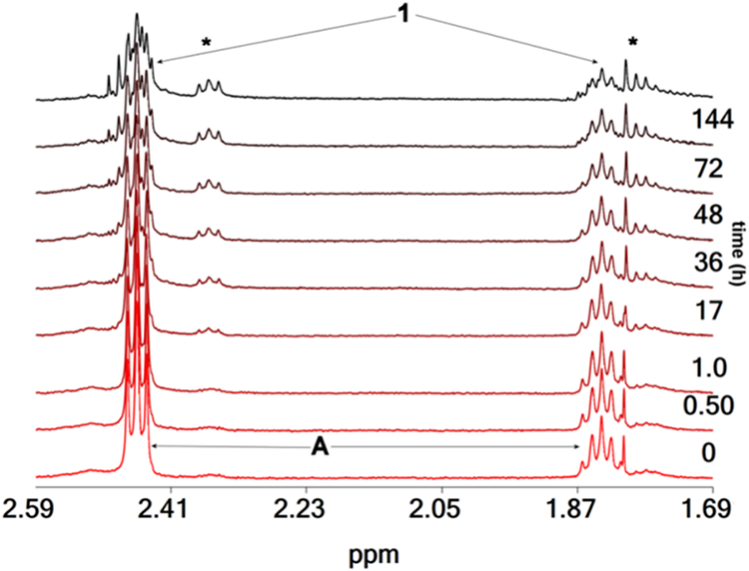
^1^H NMR spectra of H_3_pidiox (8.6 mM) and (NH_4_)_6_Mo_7_O_24_·4H_2_O [3.0 mM, 21 mM in Mo­(VI)], vs time at pD = 5.2. With an asterisk are denoted the peaks of 6-(hydroxyimino)­piperidin-2-one.

The time-resolved ^1^H NMR spectrum of a mixture of (NH_4_)_6_Mo_7_O_24_·4H_2_O and H_3_pidiox exhibited a shift of the peaks of the free ligand to lower field and it was attributed to the complexation of H_3_pidiox to molybdate forming the dioxido intermediate *cis*-[Mo^VI^O_2_(pidiox)]^−^ (species **A** in Scheme 5). The formation of species **A** was also supported by ESI-MS spectroscopy (*vide infra*). The simplicity of the ^1^H NMR spectrum of species **A** [i. e., two peaks, a triplet and a quintet (2.46 and 1.84 ppm, *J*_HaHb_ = 5.7 Hz)] (Figure 6), in comparison to the ^1^H NMR spectrum of **1** (Figure 5), is due to its higher symmetry, (species **A** has two planes of symmetry, one defined by the three atoms of the unit *cis*-[Mo^VI^O_2_]^2+^ and a second one defined by the Mo atom and the three donor atoms of the ligand pidiox^3–^). Although species **A** is hydrolytically stable at acidic pDs (∼5.5), it is hydrolyzed at pD 8.0 to give Mo^VI^O_4_^2–^ and H_3_pidiox (*cis-*[Mo^VI^(=O)_2_(pidiox)]^−^ + 2H_2_O ⇌ H_3_pidiox + Mo^VI^O_4_^2–^ + H^+^) (Figure S11). The *K*_pD=8.0_ = ([H_3_pidiox]­x­[HMo^VI^O_4_^–^])/[**A**] calculated from ^1^H NMR is ∼0.020 ± 0.004 M. The calculations are not very accurate due to the fast decomposition of the ligand. The respective *K*_pD=8.0_ for complex **1** {*K*_pD=8.0_ = ([H_3_pidiox]­x­[HMo^VI^O_4_^–^])/[**1**])} at pD = 8.0 is 100 times less than species **A**, showing the greater hydrolytic stability of complex **1** compared to species **A**.

**Scheme 5:**
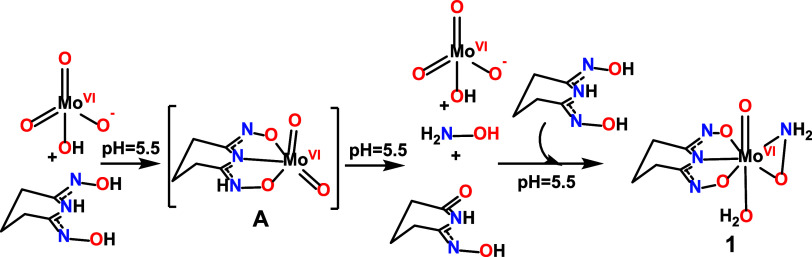
Mechanism of Hydrolysis of H_3_pidiox by [Mo^VI^O_4_]^2–^ and Formation of **1**

The hydrolysis of H_3_pidiox in the presence of molybdate Mo­(VI) salts induces the *in situ* formation of NH_2_OH, 6-(hydroxyimino)­piperidin-2-one and piperidine-2,6-dione (see Scheme 4). The hydrolysis of the pidiox^3–^ ligand coordinated to Mo^VI^ depends on the pD of the solution, and it is also correlated to the thermodynamic stability of complex **1**. The hydrolysis of H_3_pidiox in the presence of molybdate [Mo­(VI)] occurs in neutral to acidic aqueous solutions resulting in the formation of **1**. The hydrolysis of H_3_pidiox in aqueous molybdate [Mo­(VI)] solutions is slow at pD = 5.2, but nevertheless it results in the formation of **1**. However, at pD = 9, in which complex **1** is hydrolytically unstable, the aqueous solutions of H_3_pydiox-[Mo^VI^O_4_]^2–^ are stable for several weeks i.e., the ligand H_3_pidiox is not hydrolyzed and thus, the complex **1** is not formed. A possible formation mechanism of **1** is depicted in Scheme 5, where the first step requires the ligation of H_3_pidiox to *cis*-[Mo^VI^(=O)_2_]^2+^ structural unit triggering the formation of *cis*-[Mo^VI^(=O)_2_(pidiox)]^−^ (species A), and finally the formation of **1**.

#### Conversion of **1** to **3**

Aqueous solutions of compound **1** gradually convert to compound **3** at room temperature. The rate of this transformation increases with the concentration of **1** (see Figures 7 and S12–S15), as well as with the concentration of free NH_2_OH in solution at constant pD (Figures S16–S19). An increase in pD also leads to an accelerated conversion of **1** to **3** (Figures 7, 8 and S12–S15), attributable to the enhanced hydrolysis of **1**, which releases free NH_2_OH into the solution [Scheme 6(i)]. At pD 5.5, however, aqueous solutions of **1** remain stable for several weeks (Figures S12 and S17).

**Figure 7:**
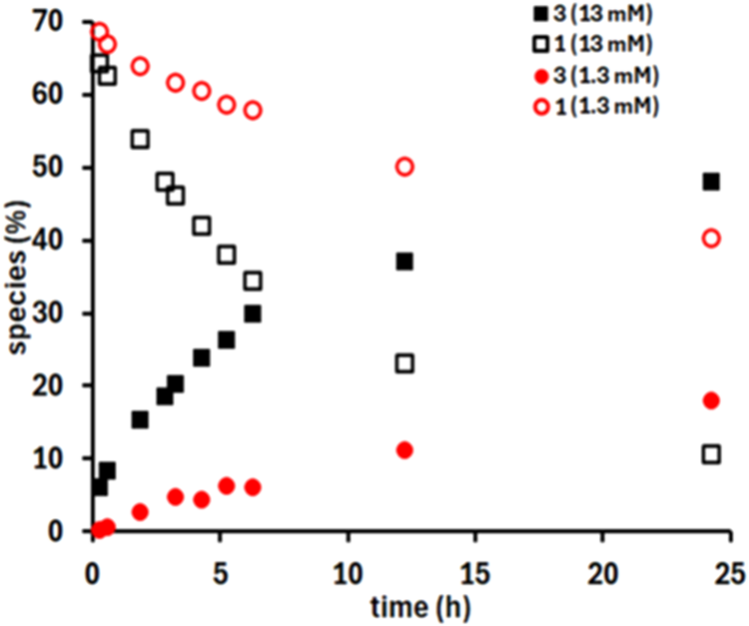
Time resolved speciation diagram of complexes **1** and **3**·2H_2_O (1.3 and 13 mM) in D_2_O at pD = 8.0 (adjusted with NaOD) based on the ^1^H NMR spectra. At pD = 8.0, 30% of **1** has been hydrolyzed.

**Figure 8:**
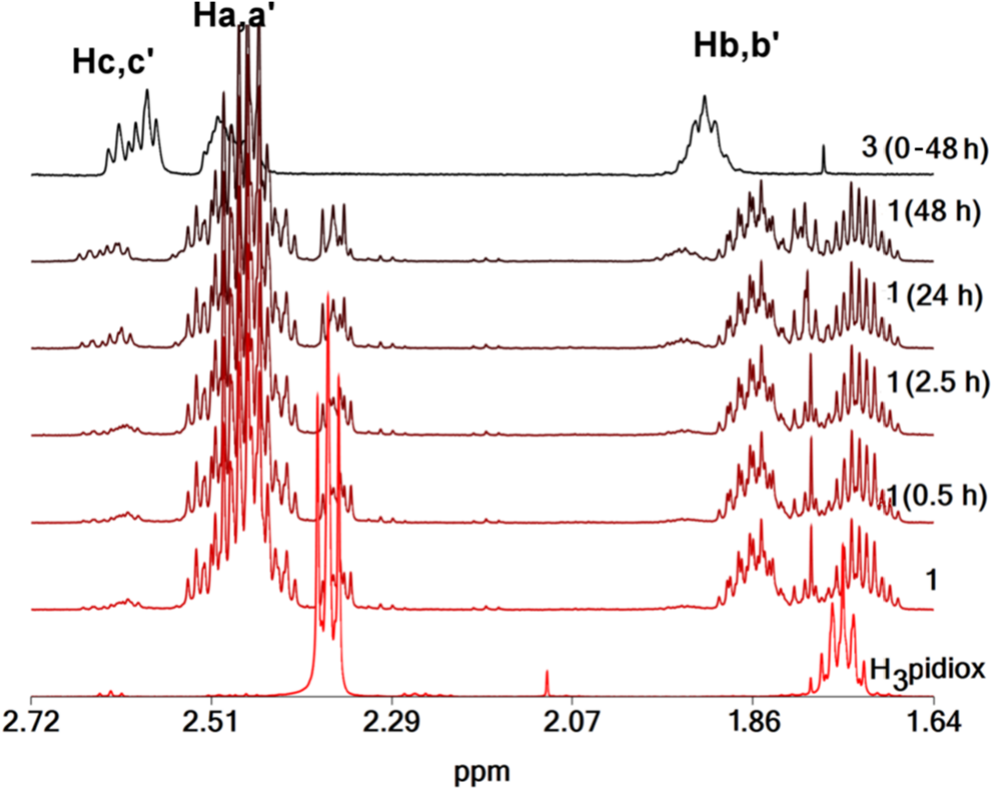
^1^H NMR spectra of H_3_pidiox, complex **1** (3.0 mM) vs time (1–48 h), and complex **3**·2H_2_O (3.0 mM) vs time (1–48 h), at pD = 6.6.

**Scheme 6:**
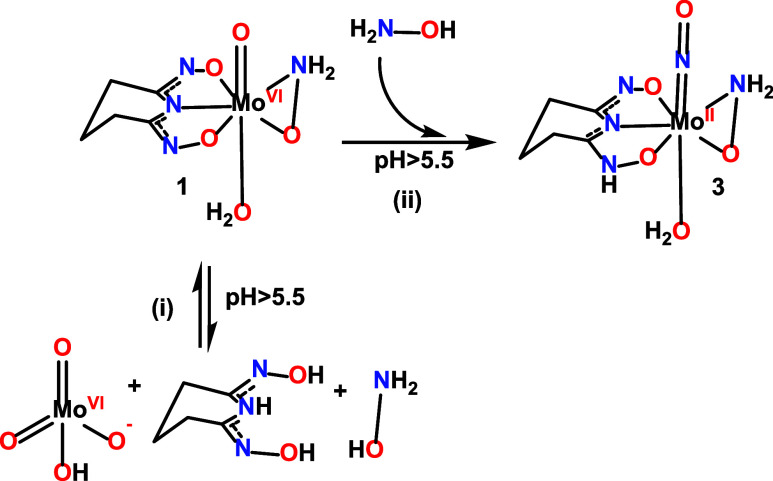
Conversion of 1 to 3 in the Presence of NH_2_OH

The mechanism underlying the conversion of compound **1** to compound **3** may proceed via either an intramolecular or an intermolecular pathway. In the intramolecular scenario, the metal-bound NH_2_O^–^ ligand induces the reduction of Mo­(VI), whereas in the intermolecular pathway, free NH_2_OH in solution is responsible for the reduction. To further elucidate the mechanism and assess the reaction rate’s dependence on the presence or absence of free hydroxylamine, a series of experiments were conducted. In these, (NH_4_)_6_Mo_7_O_24_, H_3_pidiox, and NH_2_OH were reacted in D_2_O at defined pD values, and the resulting solutions were monitored over time using ^1^H NMR spectroscopy (Figures S16–S18). The reaction at pDs within the range of 5–8 results *in immediate* formation of **1**, while the subsequent conversion of **1** to **3** is facilitated with the presence of free NH_2_OH (Figures S16 and S18). An excess of Mo­(VI) relative to H_3_pidiox and NH_2_OH shifts the equilibrium toward the formation of compound **1** [Scheme 6(i)]. The absence of free NH_2_OH in solution inhibits the conversion of **1** to **3** (Figure S16), supporting an intermolecular reaction [Scheme 6(ii)]. To further investigate the stability of **3** the release of NOx under illumination of DMSO solutions of complex **3** was investigated. The UV–vis spectra (Figure S20) show the presence of NOx in the gas phase of the irradiated solutions supporting the slow release of NOx from complex **3**.

#### ^51^V and ^1^H NMR Investigation of H_3_pidiox’s Ability to Bind V­(V) and Mo­(VI)

The hydrolysis of H_3_pidiox (15.0 mM) in the presence of equimolar quantities of [V^V^O_4_]^3–^ (10.0 mM) and [Mo^VI^O_4_]^2–^ (10.0 mM) vs time at pD = 7.9 was monitored by ^51^V and ^1^H NMR spectroscopy (Figures 9 and 10). The ^51^V NMR spectrum of V^V^O_4_^3–^/Mo^VI^O_4_^2–^ aqueous solution upon addition of H_3_pidiox shows the immediate formation of a peak at −430 ppm assigned to *cis*-[V^V^O_2_(pidiox)]^2–^. The *cis*-[V^V^O_2_(pidiox)]^2–^ is slowly converted to [V^V^(pidiox)_2_]^−^ giving a peak at −446 ppm. In addition to the peak at −446 ppm, the spectra show peaks at −559, −572 and −576 ppm assigned to monomer (V_1_), dimer (V_2_) and tetramer (V_4_) vanadate oligomers.

**Figure 9:**
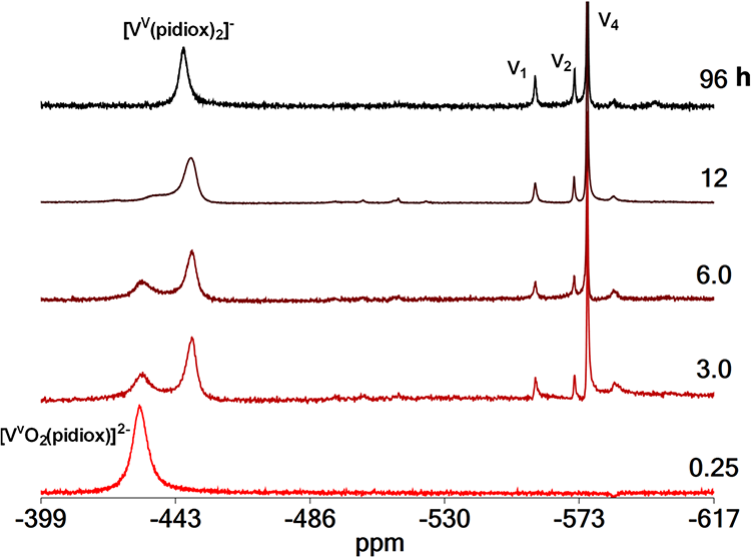
^51^V NMR spectra of a solution containing H_3_pidiox (14.0 mM), Na­[V^V^O_3_] (10.0 mM) and Na_2_[Mo^VI^O_4_]­(10.0 mM), vs time at pD = 7.9.

**Figure 10:**
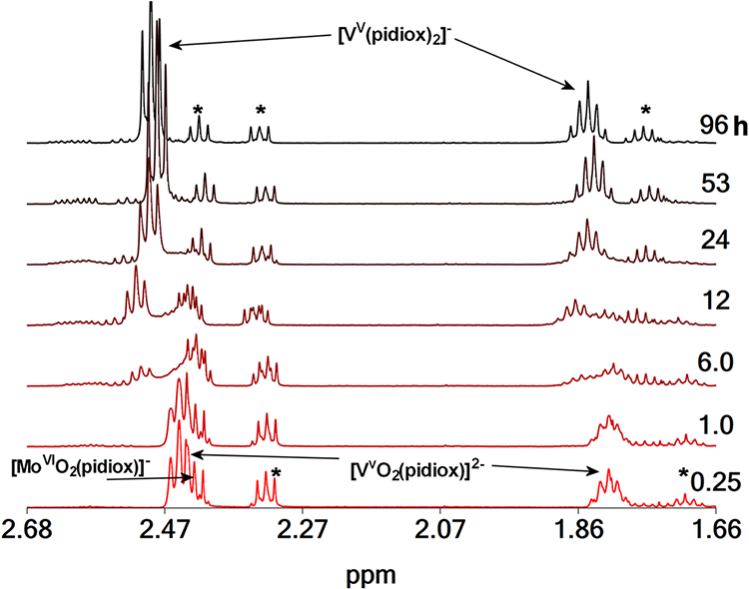
^1^H NMR spectra of a solution containing H_3_pidiox (15.0 mM), Na­[V^V^O_3_] (10.0 mM) and Na_2_[Mo^VI^O_4_] (10.0 mM), vs time at pD = 7.9. With an asterisk are denoted the peaks of H_3_pidiox and (*Z*)-6-(hydroxyimino)­piperidin-2-one.

The ^1^H NMR spectrum of [V^V^O_4_]^3–^/[Mo^VI^O_4_]^2–^ aqueous solution upon addition of H_3_pidiox (Figure 10) shows peaks at 1.82 and 2.45 assigned to *cis*-[V^V^O_2_(pidiox)]^2–^, at 1.82 and 2.43 assigned to *cis*-[Mo^VI^O_2_(pidiox)]^−^ and at 1.71, 2.32 ppm assigned to free ligand. Gradually with time the peaks are replaced with new ones at 1.85 and 2.49 ppm assigned to [V^V^(pidiox)_2_]^−^ and at 1.77, 2.33, and 2.42 ppm assigned to (*Z*)-6-(hydroxyimino)­piperidin-2-one.

The spectra show that the ligand is a much stronger binder for [V^V^O_4_]^3–^ than [Mo^VI^O_4_]^2–^. Hydrolysis of H_3_pidiox occurs only in the first 12 h, in which *cis*-[V^V^O_2_(pidiox)]^2–^ binds only 10.0 mM of H_3_pidiox, whereas 5.0 mM of H_3_pidiox are free to form [Mo^VI^O_2_(pidiox)]^−^. After the first 12 h V­(V) coordinates to pidiox^3–^ ligands forming [V^V^(pidiox)_2_]^−^, which binds all free H_3_pidiox into solution and does not allow the formation of *cis*-[Mo^VI^O_2_(pidiox)]^−^ stopping the hydrolysis of H_3_pidiox. The resulting solutions, in which 1.4 mM from the 5.0 mM of H_3_pidiox have been hydrolyzed the first 12 h of the reaction, remain stable for more than 3 weeks.

In conclusion, the markedly reduced binding affinity of oximes for Mo­(VI) relative to other metal ions found in the marine environment suggests that the degradation of oxime-based extraction materials in seawater is unlikely. This finding contradicts the observations reported by Li and colleagues.^26^

#### ESI-MS Spectrometry

In an effort to investigate the interaction of the molybdenum salts with the ligand H_3_pidiox in solution we monitored the reaction mixture as a function of time using electrospray ionization mass spectrometry (ESI-MS)^74–79^ with the aim to identify possible species generated in solution. Potential identification of intermediate species could provide additional information regarding the mechanistic aspects and operation mode of the H_3_pidiox ligand under the experimental conditions.

The ESI-MS studies were performed directly on the reaction mixture in a positive ionization mode. It was observed that the identified species in the reaction mixture formed instantly upon mixing an aqueous solution (*t* = 0) as shown in Figure S21. More specifically the dioxido and hydroxylamino based species with isotopic envelopes centered at 271.9, 286.9, and 396.1 *m*/*z* values were identified with formulas, {[MoO_2_(pidiox)]­H_2_}^+^, {[MoO­(NH_2_O)­(pidiox)]­H^+^ and {[Mo^V^O­(NH_2_O) (pidiox)]­(H_2_O)_6_H_2_}^+^ respectively. A peak at 367.9 *m*/*z* is assigned to formulas {[Mo^V^(N­(OH)_2_)­(NH_2_O)­(pidiox)]­(H_2_O)­CH_3_O}^+^ which is similar to the formula of the intermediate Im2 in Scheme 7. As a function of time the solution’s speciation does not change considerably; however, an additional dihydroxylamino species has been detected after 5 and 96 h, Figure S22, centered at 430.0 *m*/*z* values and with a formula of {[Mo­(NO)­(NH_2_O)­(Hpidiox)]­(MeOH)_4_H}^+^ presumably due to increased amount of NH_2_OH produced *in situ* in the reaction mixture as a function of time.

**Scheme 7:**
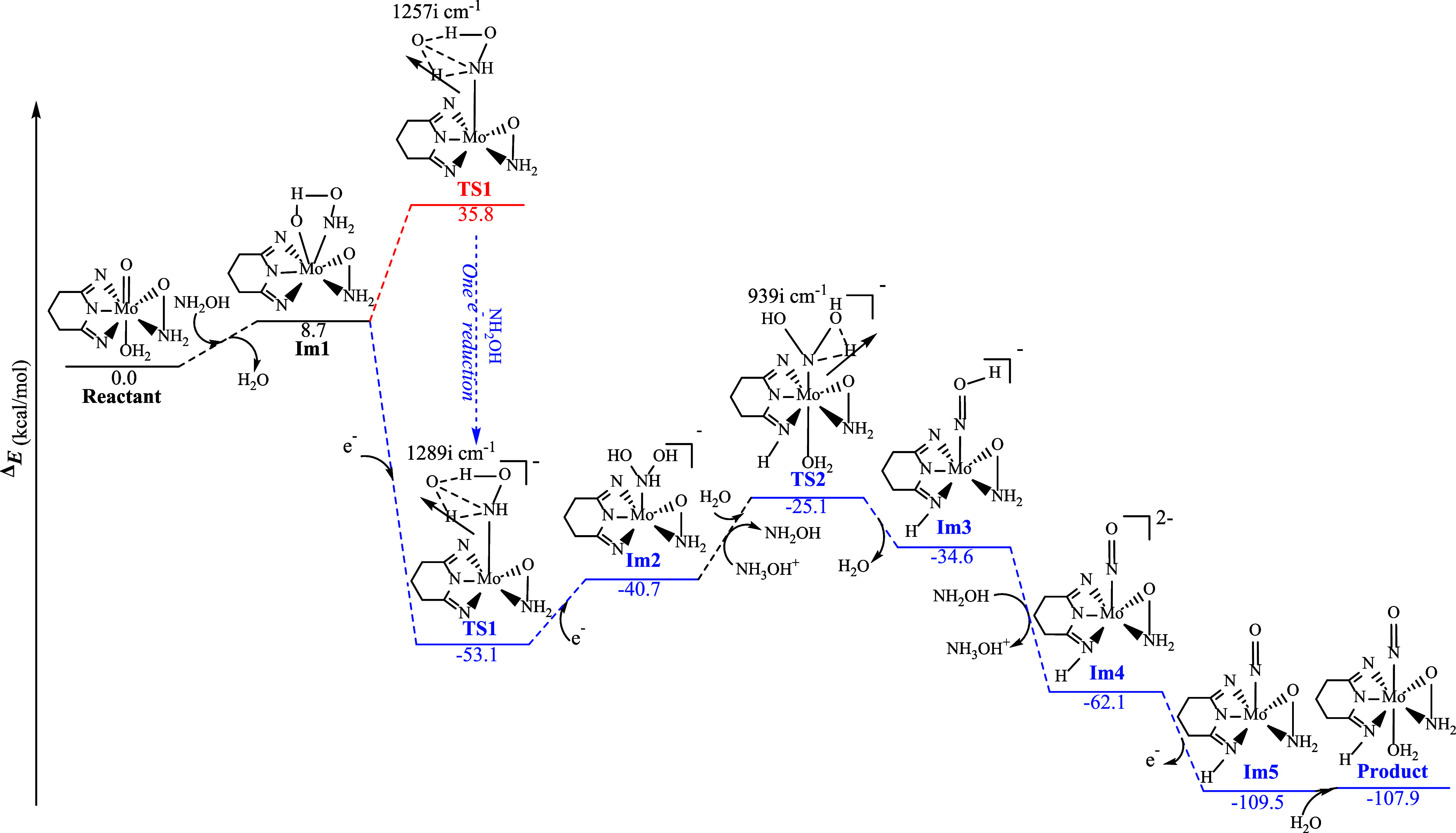
Free-Energy Reaction Profile for the Conversion of **1** to **3** Calculated at the PBE0/Def2-TZVP/(PCM, Water) Level^a^

a Coordination of NH_2_OH forms Im1 on the neutral PES; electron transfer then switches the reaction onto the reduced surface, where TS1 is located. Proton transfer and axial water coordination occur at TS2, followed by dehydration, further reduction, and oxidation steps leading to the Mo­(II)–NO product.

In a similar manner we compare the solution behavior of the complex **1** under the same experimental conditions over time at two different concentrations. In both cases the speciation of the preformed complex did not change over the period of 96 h and the main isotopic envelopes centered at 286.9, 568.9, and 588.3 *m*/*z* can be attributed to the {[MoO­(NH_2_O)­(pidiox)]­H}^+^, {[MoO­(NH_2_O)­(pidiox)]_2_H}^+^ and {[MoO­(NH_2_O)­(pidiox)]_2_(H_2_O)­H_3_}^+^ respectively, Figure S23. It is worth noting that the *in situ* alteration of the metal’s oxidation state during the course of the ion transfer it is quite common and has been reported frequently in the literature.^74,79,80^

#### Computational Study of the Conversion of **1** to **3**

Density functional theory (DFT) calculations were performed to elucidate the mechanistic details of the reduction of the Mo­(VI) complex 1 by hydroxylamine, leading to the formation of the Mo­(II) nitrosyl complex **3**. The free-energy profile, calculated at the PBE0/Def2-TZVP level in water solvent, is shown in Scheme 7.

Initially, NH_2_OH, present in the reaction mixture, is expected to coordinate through its nitrogen donor atom, displacing the axial water ligand and forming intermediate Im1. This step is only 8.7 kcal mol^–1^ uphill in free energy relative to the starting complex 1. At this stage, the reaction proceeds on the neutral potential-energy surface. After formation of Im1, an electron transfer from coordinated NH_2_OH to the Mo center takes place. This changes the electronic state of the system, and the subsequent bond-reorganization step must therefore be followed on the reduced surface. In this mechanistic scheme, TS1 belongs to the reduced PES rather than the neutral one; its apparent position “below” Im1 arises only when both surfaces are plotted on a common energy scale. The crossing of electronic surfaces is now indicated explicitly.

In the next step, the transition state TS1 is formed with concomitant reduction by NH_2_OH, which acts as the electron donor. The intrinsic solution-phase reduction free energy of TS1, obtained from PCM-solvated free energies, is Δ*G°*_red_(TS1) = −88.96 kcal mol^–1^, corresponding to a formal reduction potential of +3.9 V versus the vacuum electron. A Born–Haber cycle separation gives a gas-phase term of −45.2 kcal mol^–1^ and a differential solvation stabilization of −43.8 kcal mol^–1^, showing that solvation of the anionic state provides nearly half of the total driving force. The reduced TS1 thus lies 53.1 kcal mol^–1^ below Im1 when referenced to the reactant baseline, reflecting stabilization by one-electron reduction and justifying continuation of the mechanism on the reduced PES. The stationary nature of TS1 was verified by IRC calculations, which show continuous connection to the neighboring minima (Figure S24a).

Following the bond reorganization in TS1, the second intermediate Im2 is obtained, lying ∼12 kcal mol^–1^ above the reactant baseline. Proton transfer occurs in the next step only after the reaction has switched to the reduced PES, where the increased electron density at Mo enhances the basicity of the ligand oxygen. The subsequent step therefore involves a coupled proton-transfer and water-coordination event, proceeding through TS2 (Δ*G* ^‡^ = 15.6 kcal mol^–1^). TS2 was likewise confirmed by IRC (Figure S24b).

Loss of two water molecules from TS2 yields intermediate Im3, which is ∼9.5 kcal mol^–1^ more stable than TS2. Reduction of Im3 with concurrent deprotonation of the axial N–OH group by NH_2_OH gives intermediate Im4 in an exergonic step (ΔG = −27.5 kcal mol^–1^), producing a more reduced, deprotonated species poised for oxidation. Subsequent two-electron oxidation of [Im4]^−2^ to [Im5]^0^ is formally thermoneutral on the 1 M scale (Δ*G* ≈ 0 kcal mol^–1^) when coupled to the base-balanced NO → N_2_O redox couple and becomes increasingly favorable under near-neutral aqueous conditions. The competing O_2_ route is substantially less favorable (SI). Final water coordination to [Im5]^0^ (Δ*G* = +1.6 kcal mol^–1^) yields the neutral aqua product identified crystallographically.

Together, these results show that the sequence of electron transfer, proton transfer, and oxidation by the NO/N_2_O pool constitutes a thermodynamically viable pathway that is fully consistent with experiment. Although the formal oxidation of NH_2_OH to an N­(I) nitrosyl fragment corresponds to a two-electron process, the DFT results show that the reaction does not proceed through a single two-electron transfer. Instead, the overall electron balance is achieved through a sequence of discrete electron-transfer and proton-coupled steps, with additional reducing equivalents supplied by the NO/N_2_O redox couple under the reaction conditions.

## Conclusion

In this study, we report the first isolation and comprehensive characterization of a molybdenum­(VI) oxido-hydroxylamido complex, [Mο^VI^(O)­(η^1^,η^1^,η^1^-pidiox-*O*,*N*,*O*′)­(η^2^-NH_2_O)­(H_2_O)] (**1**), challenging the prevailing notion that molybdenum­(VI) cannot coexist with NH_2_OH, which typically acts as a reducing agent. The isolation of complex **1** enabled us to elucidate the previously unknown underlying mechanism that induces the reaction of *cis*-[Mo^VI^O_2_]^2+^ and NH_2_OH to give [Mo^II^(NO)]^3+^, a transformation that had remained unexplained for over 50 years. A combination of experimental data and DFT calculations, revealed that the reaction follows an intermolecular inner-sphere mechanism, wherein NH_2_OH attacks complex **1** to yield an eight-coordinate intermediate, [Mο^VI^(=O)­(*k* ^1^-NH_2_OH)­(η^2^-NH_2_O)­(η^1^,η^1^,η^1^-pidiox-*O*,*N*,*O*′)­(H_2_O)] (**1′**). In this intermediate, the monohapto hydroxylamine is progressively oxidized from Mo­(VI), ultimately resulting in [Mο^II^(κ^1^-NO)­(η^2^-NH_2_O)­(η^1^,η^1^,η^1^-Hpidiox-*O*,*N*,*O′*)­(H_2_O)] (**3**). The combined experimental observations and DFT calculations indicate that the electronic structure of complex **3** is most consistent with a Mo­(II) center bound to a nitrosyl ligand that behaves primarily as NO^+^. Although formal oxidation states in metal–nitrosyl systems must always be interpreted with care due to the redox noninnocence of NO, the collected metrics point consistently toward this Mo­(II)/NO^+^-leaning description. The elucidation of the redox mechanism of Mo­(VI) → Mo­(II) conversion provides a deeper insight into hydroxylamine redox chemistry relevant to molybdenum enzymes. The mechanism also highlights the importance of changes in electronic surface during the reaction, with key rearrangements occurring only after electron transfer to the metal.

Additionally, NMR and ESI-MS analyses of the interactions between H_3_pidiox and V­(V) or Mo­(VI) species indicate that the hydrolysis of H_3_pidiox by molybdate occurs upon formation of the intermediate dioxido molybdenum­(VI) complex, *cis*-[Mo^VI^(O)_2_(pidiox)]^−^, which exhibits low hydrolytic stability at pH 8.0. The presence of V­(V) in solution inhibits the decomposition of H_3_pidiox by [Mo^VI^O_4_]^2–^, as *cis*-[Mo^VI^(O)_2_(η^1^,η^1^,η^1^-pidiox-*O*,*N*,*O′*)]^−^ is not detected in solutions containing equimolar concentrations of Mo­(VI) and V­(V). These findings suggest that the degradation of oxime-based extraction materials in seawater is unlikely, since oximes display significantly weaker binding affinity for Mo­(VI) compared to other metal ions present in the marine environment.

Collectively, these findings provide molecular-level evidence for the chemical resilience of oxime-based sorbents in seawater, thereby supporting their continued optimization for large-scale extraction processes. Moreover, elucidating the underlying speciation and redox processes is crucial not only for advancing environmental chemistry, but also for paving the way for the rational design of catalysts and functional materials for energy-related applications, areas that are expected to attract considerable interest within the (bio)­chemical research community.

## Supplementary Material



## Notes

Additional experimental details, materials, and methods, XPS spectra, structural figures, additional NMR spectra, DFT UV simulation, ESI-MS spectra, mechanistic schemes, crystallographic data, Cartesian coordinates of the optimized structures (PDF)
