# Supplementary material for: Modeling Mo(VI)=O Biologically Related Interactions with Oximes and Hydroxylamines: Implications for Uranium Seawater Extraction
Source: Inorg Chem. 2026 Jan 6;65(2):1098–111. doi: 10.1021/acs.inorgchem.5c03731 (PMC12820929; doi:10.1021/acs.inorgchem.5c03731)
Supplement: Supplementary file 1 [file ic5c03731_si_001.pdf]

Supporting Information:

Modelling Mo(VI)=O Biologically Related Interactions with Oximes and Hydroxylamines: Implications for Uranium Seawater Extraction

Stamatis S. Passadis,[a,b] Maria Ch. Michaelidou,[a] Wenhao Gao,[c] Afrodite Tryfon,[d] Angelos Kalampounias,[d] John C. Plakatouras,[d] Tatjana N. Parac-Vogt,[b,*] Athanassios C. Tsipis,[d,*] Haralampos N. Miras,[c,*] Anastasios D. Keramidas,[a,*] and Themistoklis A. Kabanos[d,*]

[a] Department of Chemistry, University of Cyprus, Nicosia 2109, Cyprus. E-mail: akeramid@ucy.ac.cy

[b] Department of Chemistry, KU Leuven, Celestijnenlaan 200F, 3001 Leuven, Belgium. Email: tatjana.vogt@kuleuven.be

[c] School of Chemistry, The University of Glasgow, Glasgow G12 8QQ (UK). Email: charalampos.moiras@glasgow.ac.uk

[d] Department of Chemistry, University of Ioannina, Ioannina 45110, Greece. E-mail: tkampano@uoi.gr, attsipis@uoi.gr

Contents **Pages**

Experimental **S4**
Figure S1. Views of the 2D H-bonded sheets formed in the structures of (a) **2·2MeOH** down to [2 0 -1] direction of the unit cell and (b) **1·2MeOH** parallel to *ac* plane of the unit cell. CH hydrogen atoms have been omitted for clarity. The underlying **sql** topological networks are also shown. **S9**
Figure S2. XPS spectra of the molybdenum 3$d_{5/2}$ binding energies binding energies for compounds **1** (A) and **3**·$2H_2O$ (B) respectively. **S9**
Figure S3. A view of the 2D H-bonded layer formed in the structure of **3·2H$_2$O** parallel to *ab* plane of the unit cell. CH hydrogen atoms have been omitted for clarity. The underlying **sql** topological network is also shown. **S10**
Figure S4. (a) Experimental IR transmittance spectra of compound **1** (black line) and compound **3**·$2H_2O$ (red line) recorded under ambient conditions. **S11**
Figure S5. (a) Perturbative donor–acceptor interaction in **3**, involving the LP(Mo) (donor NBO, left) and BD*(N–O) (acceptor NBO, right); (b) 3D surfaces of the NBOs relevant to the Mo–NO bonding in **3**. **S12**
Figure S6. 3D surfaces of the bonding Mo-NO MOs in **3**. **S12**
Figure S7. Simulated absorption spectra (FWHM = 0.3 eV) of **1**, **3**·$2H_2O$ and H_3dipiox employing (a) the wavelength scale used in experiment and (b) an extended wavelength scale. **S13**
Figure S8. Simulated absorption spectra (FWHM = 0.3 eV) of **1** (black lines), **3**·(blue lines) and H_3dipiox (red lines) at the (a) CAM-B3LYP/DGDZVP/PCM(Water) level and (b) CAM-B3LYP/def2-TZVPP(+D on N,O)/PCM(H_2O) level. **S14**
Figure S9. 3D surfaces (isocontour value set to 0.08 au) of NTO pairs (*hole* - left)/*particle* – right) corresponding to the electronic transition related to the most important absorption bands found in the simulated absorption spectra of **1**. **S16**
Figure S10. 3D surfaces (isocontour value set to 0.08 au) of NTO pairs (*hole* - left)/*particle* – right) corresponding to the electronic transition related to the most important absorption bands found in the simulated absorption spectra of **3** and **H$_3$dipiox** ligand. **S17**
Figure S11. ^{1}H NMR spectra of H_3pidiox (13.0 mM) and Na_2MoO_4 (13.0 mM) at pD = 8.0. **S18**
Figure S12. ^{1}H NMR spectra of D_2O solutions of complex **1** (1.3 mM) *vs* time at pD=5.6. **S19**
Figure S13. ^{1}H NMR spectra of D_2O solutions of complex **1** (13 mM) *vs* time at pD=5.4. **S19**
Figure S14. ^{1}H NMR spectra of D_2O solutions of complex **1** (13 mM) intense orange color *vs* time at pD=8.0 (adjusted with NaOD). * free H_3pidiox6-(hydroxyimino)piperidin-2-one and piperidine-2,6-dione. **S20**
Figure S15. ^{1}H NMR spectra of D_2O solutions of complex **1** (1.3 mM) yellow orange color *vs* time at pD=8.0 (adjusted with NaOD). free H_3pidiox6-(hydroxyimino)piperidin-2-one and piperidine-2,6-dione. **S21**
Figure S16. ^{1}H NMR spectra of the D_2O solutions of $(NH_4)_5Mo_7O_{24}$ (1.50 mM), 10.5mM/Mo , H_3pidiox (2.10 mM) and NH_2OH (2.10 mM) at pD 6.0 *vs* time. **S21**
Figure S17. ^{1}H NMR spectra of D_2O solutions of $(NH_4)_5Mo_7O_{24}$ (0.75mM) , 5.25mM/Mo + H_3pidiox (10.5 mM) + NH_2OH (10.5mM) *vs* time at at pD=7.5 **S22**
Figure S18. ^{1}H NMR spectra of D_2O solutions of $(NH_4)_5Mo_7O_{24}$ (0.75mM) , 5.25mM/Mo + H_3pidiox (10.5 mM) + NH_2OH (21.0mM) *vs* time at at pD=7.5 **S22**
Figure S19. 2D{^{1}H,^{13}C}HMBC NMR spectra of the D_2O solutions of $(NH_4)_5Mo_7O_{24}$ (3.0 mM Mo^{VI}) and H_3pidiox (3.0 mM) at pD 6.0 after 8 days. **S23**
Figure S20. The UV-vis measurements of vials 3 (red line) and 4 (black line) **S24**
Figure S21. Positive mode ion mass spectrum of reaction mixture S25

$(NH_4)_6Mo_7O_{24}$ (7.5mM), and H_3pidiox (105 mM) in MeOH:H_2O at t=0.
Figure S22. Positive mode ion mass spectrum of reaction mixture $(NH_4)_6Mo_7O_{24}$ (7.5mM), and H_3pidiox (105 mM) in MeOH: H2O at t=96hrs. **S25**
Figure S23. Positive mode ion mass spectrum of complex **1** (1.3 mM) in MeOH:H_2O at t=96hrs. **S26**
Figure S24. Intrinsic reaction coordinate (IRC) energy profiles for TS1 and TS2 computed at the PBE0/def2-TZVP/PCM(water) level of theory. Energies are expressed as relative Gibbs free energies (in kcal·mol^{-1}) with respect to the lowest-energy point along each path. **S27**
Figure S25. Photodegradation experiments detail **S28**
Figure S26. CVs of **1** (5.0 mM) and **3**·2H_2O (5.0 mM) in solution (DMSO). Working Electrode, glassy carbon disk, Auxiliary Electrode platinum wire, Reference Electrode, Ag/AgCl (0.2 V *vs* NHE measured with Ferrocene 0.65 V *vs* NHE). **S28**
Table S1. Crystal data and structure refinement for the prepared compounds. **S29**
Table S2. H-bonds 1·2CH_3OH **S30**
Table S3. H-bondsof **2**·2CH_3OH **S30**
Table S4. H-bonds **3**·2H_2O **S31**
Table S5. Comparison of Experimental and TDDFT-Computed UV–Vis Absorption Maxima (in nm) for the Reactant, **1** Product, **3** and H_3dipiox Ligand. **S32**
Table S6. Cartesian coordinates and energetic data of the optimized geometries of all species involved in the reaction mechanism of **1** to **3** conversion calculated at the PBE0/Def2-TZVP level in water solvent (gas phase calculations for some species were also performed as indicated in the species legends). **S33**
Table S7. Cartesian coordinates of the geometries corresponding to the terminal points of the intrinsic reaction coordinate (IRC) paths computed at the **PBE0/Def2-TZVP/PCM(water)** level. These endpoint structures correspond to geometries located near the potential-energy minima connected by the respective transition states (TS1 and TS2) and confirm the correct connectivity along each reaction path. **S40**

Experimental Details

Crystal Structure Determinations

Diffraction data for **1**·$2CH_3OH$ were collected on a Bruker D8 Quest Eco diffractometer, equipped with a Photon II detector and a TRIUMPH (curved graphite) monochromator at room temperature. Suitable single crystal was selected and mounted adequately on the goniometer heads and single-crystal X-ray diffraction data of **2**·2MeOH were recorded on a Bruker Apex CCD diffractometer at 150 K equipped with a graphite monochromator, while data for **3**·$2H_2O$ were collected on an Xcalibur Oxford Diffractometer equipped with a Sapphire 3 CCD detector and a 4-cycle Kappa geometry goniometer at 150 K. Mo Ka radiation (λ = 0.71073 Å) was utilized for all measurements. The collected frames were integrated with instrument software and corrected for absorption. The structures were solved with SHELXT (direct methods)[1] and refined by full-matrix least squares techniques on F2 (SHELXL 2018/3)1 via the ShelXle interface.[2] The non-H atoms were treated anisotropically, whereas the organic H atoms were placed in calculated, ideal positions and refined as riding on their respective carbon atoms. In all compounds, hydroxylaminate and coordinated solvent protons were located from different Fourier maps and refined with SHELX restraints; solvated methanol or water proton positions were calculated with Calc-OH[3] implemented in WinGX[4] and were refined with SHELX restraints. PLATON[5] was used for geometric calculations, and Diamond[6] and X-Seed[7] for molecular graphics.

Details on data collection and refinement are presented in Table S1. Full details on the structures can be found in the CIF files deposited with CCDC. CCDC 2432328, 2411874, and 2432329 for complexes **1**·$2CH_3OH$, **2**·$2CH_3OH$, and **3**·$2H_2O$ respectively, contain the supplementary crystallographic data for this paper. These data can be obtained free of charge via www.ccdc.cam.ac.uk/data_request/cif.

XPS Measurements. XPS were performed on a Kratos AXIS Supra+ equipped with a dual source X-ray filament (Ag/Al).

NMR measurements. All NMR samples were prepared from crystalline molybdenum(VI) compounds in D2O at room temperature just prior to NMR spectrometric determinations. NMR spectra were recorded on a Bruker Avance III 500 MHz spectrometer. A 30°-pulse width was applied for the ^{1}H NMR (500.03 MHz) measurements, and 2 s relaxation delay d1. A 90°-pulse width was applied for the ^{51}V NMR (131.44 MHz) measurements, and 2 ms relaxation delay d1. The sweep width for ^{1}H and ^{51}V spectra was 2500 and 33000 Hz respectively. TOPSPIN standard parameters were used for the acquisition of 2D {^{1}H,^{13}C} grHMBC/grHSQC; F2 dimension: (^{1}H 500.03 MHz) number of points 4k, SW 1900 Hz, NS 64, d1 1.5 s, F1 Dimension: (^{13}C 125.75 MHz) number of points 256, SW 28000 Hz. Data acquisition and processing were accomplished using TopSpin 4.0.6 and MultiSpecNMR 4.0.0 (https://sourceforge.net/projects/multispecnmr/). Standard pulse programs implemented in TopSpin were used for data acquisition.

Sample Preparation for ^{1}H & ^{51}V NMR and UV-Vis measurements. The kinetic UV-vis measurements were recorded on a Photonics UV-vis spectrophotometer Model 400, equipped with a CCD array, operating in the range 250 to 1000 nm.

For the ^{1}H and ^{51}V NMR measurements the stock solutions (20.0 and 50.0 mM) were prepared by dissolving crystalline materials in D_2O. The final solutions were prepared by mixing the appropriate quantities of the stock solution and the pD of the solutions were adjusted by the addition of DCl/NaOD. The values recorded from the pH-meter for D_2O solutions are lower than the pH values by 0.407 units, so this value was added to the pH readings to get the pD values.[8] In the mixed metal NMR experiments a solution of $NaVO_3$ was reacted with H_3pidiox before the addition of MoO_4^{2-} in order to avoid premature decomposition of the ligand. The solutions for UV-vis measurements were prepared with a similar to NMR way in H_2O.

Cyclic Voltametry - Experimental Procedure

Cyclic voltammetry (CV) experiments were recorded using a CHI650E electrochemical workstation (CH Instruments) configured as a potentiostat/galvanostat. All electrochemical measurements were performed using a standard three-electrode system, consisting of a glassy carbon as the working electrode, a platinum wire as the auxiliary electrode, and a Ag/AgCl(s) electrode as a reference electrode. The electrochemical measurements were carried out in

DMSO solutions containing 5.0 mM of compound **1** and 5.0 mM of compound **3·2H₂O**, in the presence of tetrabutylammonium tetrafluoroborate (0.3 M) as the supporting electrolyte. The solutions were purged with Ar prior to each measurement to remove dissolved oxygen. CV scans were recorded at a scan rate (ν) of 100 mV s^{-1} at 298 K. All the potential values are reported versus the Ag/AgCl(s) saturated reference electrode.

ESI-MS Experimental Details

All MS data were collected using a Q-trap, time-of-flight MS (Maxis Impact MS) instrument supplied by Bruker Daltonics Ltd. The detector was a time-of-flight, micro-channel plate detector and all data was processed using the Bruker Daltonics Data Analysis 4.1 software, whilst simulated isotope patterns were investigated using Bruker Isotope Pattern software and Molecular Weight Calculator 6.45. The calibration solution used was Agilent ES tuning mix solution, Recorder No. G2421A, enabling calibration between approximately 100 *m/z* and 2000 *m/z*. This solution was diluted 60:1 with MeCN. The reaction mixture was introduced into the MS *via* direct injection at 180 μL h^{-1}. The ion polarity for all MS scans recorded was positive, at 180 °C, with the voltage of the capillary tip set at 4000 V, endplate offset at –500 V, funnel 1 RF at 300 Vpp and funnel 2 RF at 400 Vpp.

FT-IR spectroscopy

All Fourier Transform mid-infrared spectra were recorded for all compounds in transmittance mode in the range from 4000 to 400 cm^{-1} by means of the compact Jasco FTIR-4700 spectrophotometer (Jasco, Tokyo, Japan) equipped with a stable 45° Michelson interferometer. A standard ceramic source of high intensity was used as a light source. The signal was detected by a stable Peltier-cooled DLATGS detector of high sensitivity. The spectral resolution was set at 2 cm^{-1} for all measurements. An adequate signal-to-noise ratio was achieved with 256 scans. Approximately 2 mg of the sample in the solid state was mixed with ~200 mg spectroscopic grade anhydrous KBr powder and subsequently pressed into pellets with 1 mm thickness. To account for the compensation of the atmospheric vapor, the spectrum of a pellet with pure KBr was recorded and subtracted from each FTIR measurement.

Computational Details

All quantum-chemical calculations were performed using the Gaussian 16 C.01 program package.[8] Geometries of all species were optimized without symmetry constraints employing the hybrid density functional of Perdew, Burke, and Ernzerhof (PBE0), which mixes 25 % Hartree–Fock exchange with 75 % PBE exchange–correlation.[9-14] The def2-TZVP basis set of Weigend and Ahlrichs[15] was used for all atoms as implemented in the Gaussian 16 C.01 program suite. This basis is a contracted triple-ζ valence set with polarization functions, providing balanced accuracy across the periodic table. For heavier elements such as molybdenum, def2 effective core potentials (ECPs) were employed to account for scalar relativistic effects. The resulting computational protocol is referred to as PBE0/def2-TZVP throughout. All stationary points were characterized by analytical frequency calculations at the same level of theory. Minima were confirmed by the absence of imaginary frequencies (NImag = 0), and transition states (TSs) were verified as first-order saddle points possessing a single imaginary vibrational mode corresponding to motion along the reaction coordinate. The connectivity of each TS to its adjacent intermediates was established through intrinsic reaction coordinate (IRC) calculations, confirming correct location on the potential-energy surface. Thermochemical corrections to Gibbs free energies were derived from harmonic vibrational frequencies at 298.15 K and 1 atm, without frequency scaling. Solvent effects were incorporated via the integral equation formalism variant of the polarizable continuum model (IEF-PCM) for water, using the default self-consistent reaction field (SCRF) scheme implemented in Gaussian 16.[16] Electronic absorption spectra were simulated using time-dependent DFT (TDDFT) at the PBE0/def2-TZVP/PCM(H_2O) level, which gave the best agreement with experiment among several tested functionals and basis sets (see Table SX). Benchmark calculations at the CAM-B3LYP/DGDZVP and CAM-B3LYP/def2-TZVPP(+D) levels confirmed the robustness of the chosen protocol. In all TDDFT calculations the first 30 excited states were taken into account. Natural Bond Orbital (NBO) analyses were performed using Weinhold's NBO 3.1 program as implemented in Gaussian 16 software[17] to evaluate bonding characteristics, donor–acceptor interactions, and natural charge distributions.

Complementary wavefunction analyses were carried out using Multiwfn (version 3.8)[18] to obtain molecular orbital compositions, charge-density maps, and orbital interaction diagrams, providing a detailed picture of metal–ligand bonding and NO linkage isomerization. To simplify UV-Vis the assignment, we have calculated the Natural Transition Orbital (NTO) *hole,* (h^+)/*particle* (e^-) pairs introduced by Martin.[19]

General Computational Details

Calculations were done using *Gaussian 16* (Rev. C.01) employing the PBE0/Def2-TZVP protocol taking into account the water solvent effects with the PCM solvation model. Free energies (G) correspond to the "*Sum of electronic and thermal Free Energies*" at 298 K and 1 M standard state. All minima and transition states were verified by frequency analysis (NImag=0 and NImag = 1 respectively). Energies include zero-point and thermal corrections. For bulk water, a standard-state correction of $-RT\ln 55.34 = -2.378$ kcal mol^{-1} was applied.

Calculation of the Free Energy of Reduction and Reduction Potential for TS1

Thermodynamic framework

The free energy for reduction of TS1, $\Delta G^{0}_{soln,red}$(TS1) was calculated using the following equation:

$\Delta G^{0}_{soln,red}(TS1) = G_{soln}(TS1^{-}) - G(TS1^{0})$

(G values are the *Sum of electronic and thermal Free Energies* (298 K, 1atm) (see Table below) computed at the PBE0/Def2-TZVP level in water solvent.

Reduction potential was calculated using the equation

$E^{0}_{red}(TS1) = -\Delta G^{0}_{soln,red}/F$

(F is the Faraday constant being equal to 23.061 kcal/mol V).

Calculation of $\Delta G^{0}_{soln,red}$(TS1) was done using the following equation pertaining the Born – Haber cycle:

$\Delta G^{0}_{soln,red} = [G_{gas}(\mathbf{TS1^{-}}) - G_{gas}(\mathbf{TS1^{0}})] + [\Delta G_{solv}(\mathbf{TS1^{-}}) - \Delta G_{solv}(\mathbf{TS1^{0}})]$

where

$\Delta G^{0}_{gas,red} = [G_{gas}(TS1^{-}) - G_{gas}(TS1^{0})]$

$\Delta\Delta G^{0}_{solv} = [\Delta G_{solv}(TS1^{-}) - \Delta G_{solv}(TS1^{0})]$

and

$\Delta G_{solv}(TS1^{q}) = G_{soln}(TS1^{q}) - G_{gas}(TS1^{q})]$ (q = -1 or 0)

Numerical results obtained at the PBE0/Def2-TZVP@PCM(Water) protocol

Quantity	$TS1^{0}$	$TS1^{-}$	Difference (a.u.)	Difference (kcal/mol)
G_{gas} (a.u.)	-914.417885	-914.489836	-0.071951	-45.15
G_{soln} (a.u.)	-914.441805	-914.583567	-0.141762	-88.96
Solvation free energy (a.u.)	-0.023920	-0.093731	-0.069811	-43.81

$\Delta G^{0}_{soln,red}$ (TS1) = -0.141762 a.u. = -88.96 kcal/mol

E^{0}_{red} (TS1) = +3.86 V (1e^{-})

The total stabilization therefore derives from both gas-phase electronic reorganization (-45.2 kcal/mol) and solvation (-43.8 kcal/mol).

Combining $\Delta G^{0}_{soln,red}$ (TS1) with the relative energies of the stationary points yields the placement of TS1:

$\Delta G_{rel}(TS1^{-}) = G(TS1^{-}) - G(Im1) + \Delta G^{0}_{soln,red}$

leading to TS1^{-} located 53.1 kcal mol^{-1} below Im1 relative to the reactant baseline. This energetic stabilization confirms that the reaction proceeds on the reduced potential-energy surface.

Thermochemistry of Reductions and Proton Transfers

Proton Transfer (Im3 → Im4)

Deprotonation of the axial N-OH ligand in Im3 by hydroxylamine is thought to proceed via the following reaction:

$[Im3]^{-} + NH_2OH \rightarrow [Im4]^{2-} + NH_3OH^{+}$

with

$\Delta G_{PT} = G([Im4]^{2-}) + G(NH_3OH^{+}) - G([Im3]^{-}) - G(NH_2OH)$

ΔG_{PT} = -0.043793 au or <u>-27.5 kcal/mol</u>

This step is strongly exergonic, accounting for spontaneous formation of $[Im4]^{2-}$ upon reaction with NH_2OH. The G values calculated for the species involved in this step are as follows:

Species	G (a.u.)
Im3	-838.783285
Im4	-838.406557
NH_2OH	-131.617949
NH_3OH^+	-132.038470

Two-Electron Oxidation ($[Im4]^{2-} \rightarrow [Im5]^{0}$)

$\Delta G_{2e}^{int} = G([Im5]^0) - G([Im4]^{2-}) = +0.262644$ a.u. = +164.81 kcal/mol

Oxidant half-reactions

i) $NO \rightarrow N_2O$

$2NO + H_2O + 2e^- \rightarrow N_2O + 2OH^-$

$\Delta G_{red}^{(2e)} = -164.63$ kcal/mol

$\Delta G_{ox} = +0.18$ kcal/mol (~ thermoneutral)

ii) O_2 route

$O_2 + H_2O + 2e^- \rightarrow HO_2^- + OH^-$

$\Delta G_{red}^{(2e)} = -122.24$ kcal/mol

$\Delta G_{ox} = +42.6$ kcal/mol (endergonic)

Although the formal 1M free energies are pH-independent the $NO \rightarrow N_2O$ half-reaction produces OH^-. Upon inclusion of analytic correction $-m(14\text{-pH})RT\ln 10$ (m = 2) renders this step exergonic near neutrality, signifying the NO/N_2O pool as the most likely oxidant.

Water Association ($[Im5]^0 \rightarrow$ Product)

$[Im5]^0 + H_2O \rightarrow$ Product

$\Delta G_{bind}^{(1M)} = +4$ kcal/mol, $\Delta G_{bind} = +1.6$ kcal/mol (corrected)

Correction has been done for the ΔG_{bind} since Gaussian16 software reports solution free energies at the 1M standard state for all species. When a reaction binds an explicit solvent molecule (H_2O) the 1M convention overestimates the entropic penalty because bulk water in liquid water is ~ 55.34 M and not 1M. To represent the real thermodynamic driving force for pulling one water molecule from the bulk, we correct association free energy by replacing the standard-state activity of water (1M) with its bulk concentration (55.34 M). For a reaction that consumes one H_2O (no H_2O on the product side) the correction is:

$\Delta G_{bind} = \Delta G_{bind}^{(1M)} - RT\ln(55.34)$

At 298 K $RT\ln(55.34) = 2.378$ kcal/mol.

For our reaction step

$[Im5]^0 + H_2O \rightarrow$ Product

we will have

$\Delta G_{bind}^{(1M)} = G(\text{Product}) - G([Im5]^0) - G(H_2O) = +4$ kcal/mol

and after correction

$\Delta G_{bind} = +4 - 2.378 = \underline{+1.6 \text{ kcal/mol}}$

Summary of Computed Thermochemical Steps

Reaction	ΔG (kcal/mol)	Comment
$[Im1]^0 \rightarrow TS1^-$	-53.1	Reduction stabilized TS
$Im3^- + NH_2OH \rightarrow Im4^{2-} + NH_3OH^+$	-27.5	Proton transfer, favorable
$Im4^{2-} \rightarrow Im5^0$ ($NO \rightarrow N_2O$)	≈ 0	Thermoneutral st 1M, exergonic in H_2O
$Im4^{2-} \rightarrow Im5^0$ (O_2)	+42.6	Endergonic, disfavored
$Im5^0 + H_2O \rightarrow Product^0$	+1.6	Weakly endergonic binding

NOx detection experiment

Photodegradation experiment. This experiment is founded on the Griess test, which identifies the presence of NO_2^- through quantitative spectrophotometric analysis. Given that the degradation products are anticipated to encompass nitrogen oxides (NO_x), NaOH was employed as the absorption solution to transform the gaseous NO_x into $NaNO_2$, which was subsequently analyzed utilizing the Griess test.

Stock solutions. Sulfanilamide: A stock solution was prepared by dissolving 1000.8 mg (5.81 mmol) of sulfanilamide in 30 mL of H_2O, followed by the addition of 6 mL of H_3PO_4. The mixture was then sonicated to aid dissolution. Finally, the solution was diluted to 100 mL with deionized water, resulting in a final concentration of 58.1 mmol/L. N-(1-naphthyl)ethylenediamine: A stock solution was prepared by adding 100.0 mg (0.39 mmol) of N-(1-naphthyl)ethylenediamine dihydrochloride to 50 mL of H_2O in a beaker. The mixture was stirred for 2 hours, then filtered through filter paper to clarify it. Finally, the filtrate was diluted to 100 mL with deionized water. NaOH: A total of 319.7 mg (8.0 mmol) of NaOH was dissolved in 40 mL of water to prepare a 0.2 mol/L NaOH solution.

Samples Preparation: Two vials containing 10.3 mg (**vial 1**) and 5.2 mg (**vial 2**) of compound **3** were combined with 10 ml of DMSO. Subsequently, the mixtures were subjected to sonication to facilitate dissolution (at this stage, the solutions exhibited a yellow hue) and were sealed with rubber stoppers to maintain airtight conditions. Following complete dissolution, vial 1 was subjected to 100 W UV illumination for a duration of 20 minutes. At the conclusion of the experiment, the solution had developed a more intense yellow color.

Samples Preparation for UV-vis measurements (Figure S25): Two vials (**vial 3** and **vial 4**) containing 5 mL of NaOH stock solution, followed by the injection of 5 mL of headspace gas from **vial 1** (post-illumination) into **vial 3** and from **vial 2** (after illumination) into **vial 4** After the gas injection, the pH of the solution was adjusted to 12.84 using HCl. Then, 2.5 mL of sulfanilamide solution was introduced to **vial 3** and **vial 4**, and the mixtures were stored in the dark for 3 minutes. Subsequently, 2.5 mL of N-(1-naphthyl)ethylenediamine solution was added, resulting in a yellow coloration of the solutions. The mixtures were left to develop in the dark for 3 hours, during which the colour of the solution changed to pink. UV–vis measurements were then conducted on both vials.

The baseline was corrected with a blank sample (**Vial 5**). The vial contained 5 mL of NaOH. The pH was adjusted to 12.29 by addition of HCl. Then, 2.5 mL of sulfanilamide solution was introduced to **vial 5** (in a similar fashion to **vials 3** and **4**), and the mixtures were stored in the dark for 3 minutes. Subsequently, 2.5 mL of N-(1-naphthyl)ethylenediamine solution was added, resulting in a yellow coloration of the solutions. The mixtures were left to develop in the dark for 3 hours. During this period of time though the final colour of this sample remained yellow.

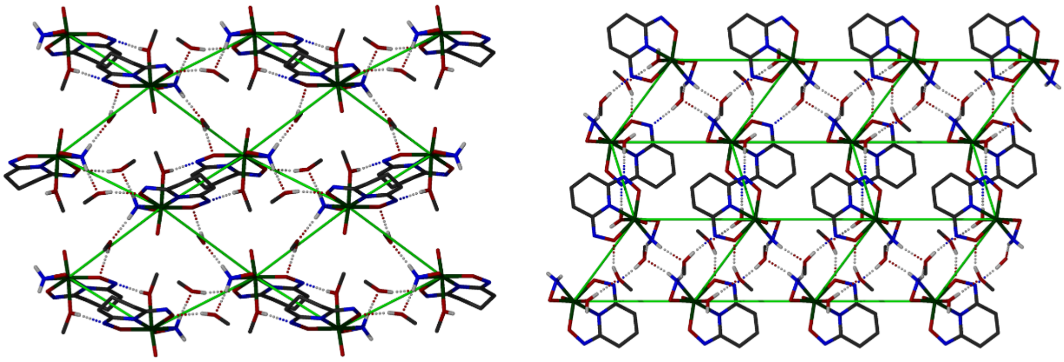

(a) (b)

Figure S1. Views of the 2D H-bonded sheets formed in the structures of (a) **2·2MeOH** down to [2 0 -1] direction of the unit cell and (b) **1·2MeOH** parallel to *ac* plane of the unit cell. CH hydrogen atoms have been omitted for clarity. The underlying **sql** topological networks are also shown.

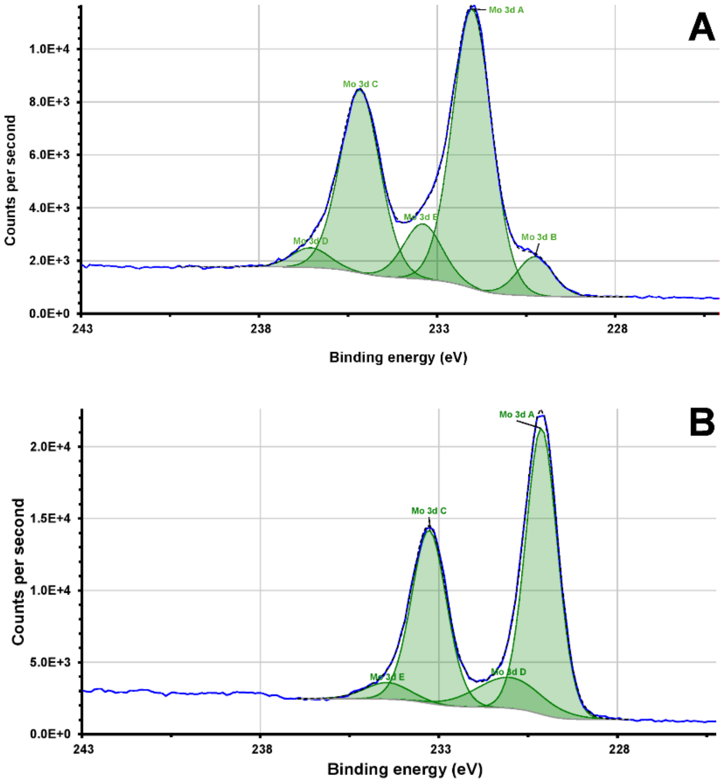

Figure S2. XPS spectra of the molybdenum $3d_{5/2}$ binding energies binding energies for compounds **1** (A) and **3**·$2H_2O$ (B) respectively.

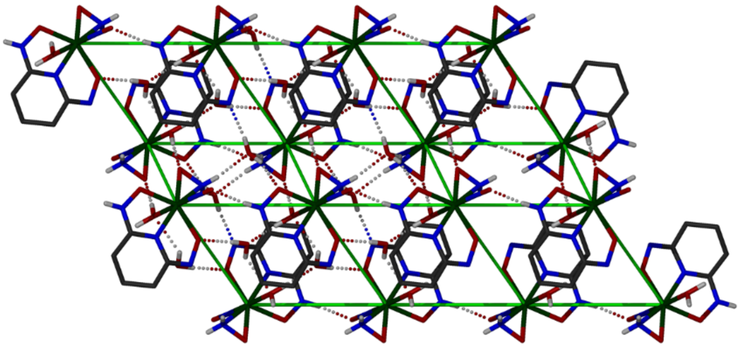

Figure S3. A view of the 2D H-bonded layer formed in the structure of **3·2H$_2$O** parallel to *ab* plane of the unit cell. CH hydrogen atoms have been omitted for clarity. The underlying **sql** topological network is also shown.

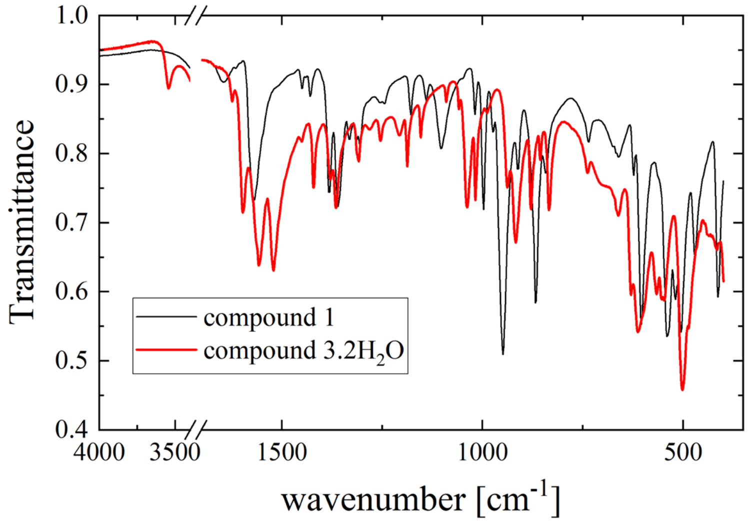

Figure S4. (a) Experimental IR transmittance spectra of compound **1** (black line) and compound **3**·$2H_2O$ (red line) recorded under ambient conditions.

The high-frequency region of the spectra is dominated by the presence of the N–H stretching mode of complex **3**·$2H_2O$, which is absent in the spectrum of compound **1**. The vibrational frequency of this mode is observed experimentally at 3543 cm^{-1}, whereas the corresponding theoretically predicted frequency was estimated to be equal to 3467 cm^{-1}.

The fingerprint region of the spectra is characterized by several sharp, intense and strongly overlapping bands reflecting the inherent structural complexity of the molybdenum-based complexes. In the medium frequency range, three main bands are observed in the spectrum of complex **3**·$2H_2O$. The band observed at 1624 cm^{-1} corresponds to the symmetric stretching vibration of the C=N bonds while the band at 1597 cm^{-1} is attributed to the stretching vibration of N-O (the nitrosyl group). Furthermore, the experimental spectrum of compound **1** exhibits extensive deviation compared to that of compound **3**·$2H_2O$. The peak of compound **1** observed experimentally at ~1569 cm^{-1} is attributed to the symmetric stretching vibration of the C = N bond.

At lower frequencies, four prominent bands are observed in the FT-IR spectrum of compound **1**. The band at 948 cm^{-1} is assigned to the Mo=O stretching vibration, while the band at 911 cm^{-1} corresponds to the N–O stretching vibration of the hydroxylamine ligand coordinated to the molybdenum(VI) atom. Finally, the bands at 868 and 842 cm^{-1} are associated with the symmetric stretching and the breathing mode of the ring, respectively. In the experimental spectrum of compound **3**·$2H_2O$, the band observed at 938 cm^{-1} is assigned to the N-O stretching vibration of the hydroxylamine group, while the band at 917 cm^{-1} is attributed to the C=**N-O**$^-$ stretching vibration, of the group double-bonded to the piperidine ring. The vibrations at 879, 856 and 834 cm^{-1} correspond to the symmetric stretching, asymmetric stretching, and breathing modes of the ring, respectively. The theoretically estimated frequencies of these modes are all red shifted.

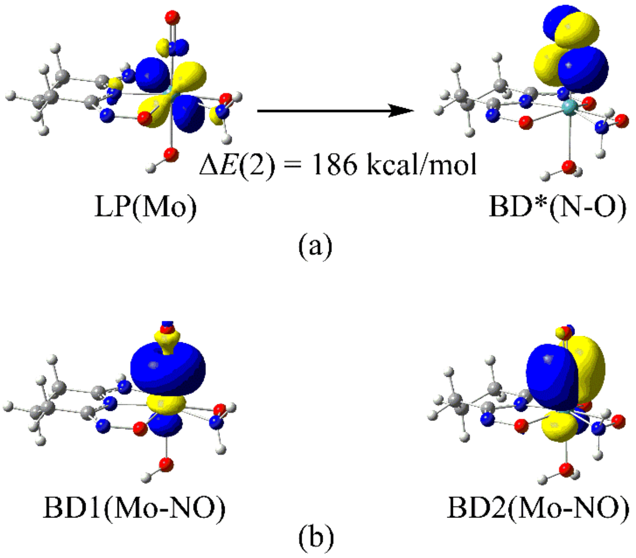

Figure S5. (a) Perturbative donor–acceptor interaction in **3**, involving the LP(Mo) (donor NBO, left) and BD*(N–O) (acceptor NBO, right); (b) 3D surfaces of the NBOs relevant to the Mo–NO bonding in **3**.

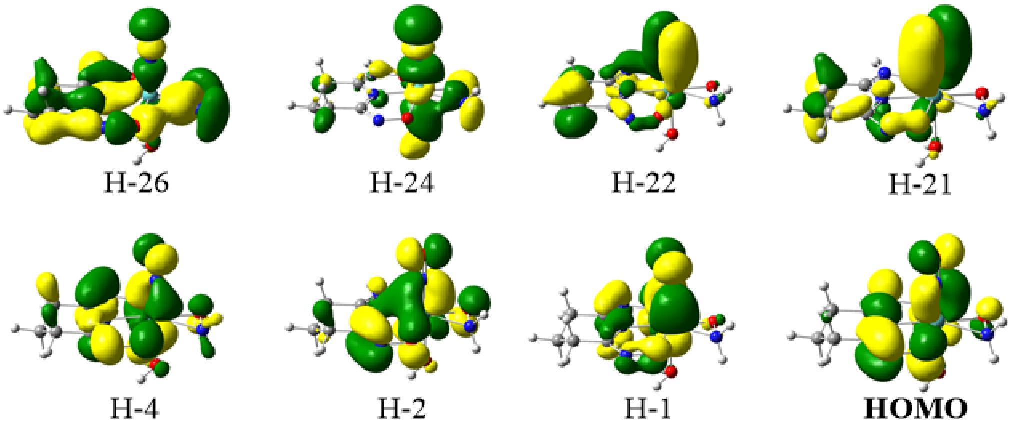

Figure S6. *Kohn–Sham orbitals relevant to Mo–NO bonding, showing delocalized π and σ interactions between Mo d and NO π/π** orbitals.

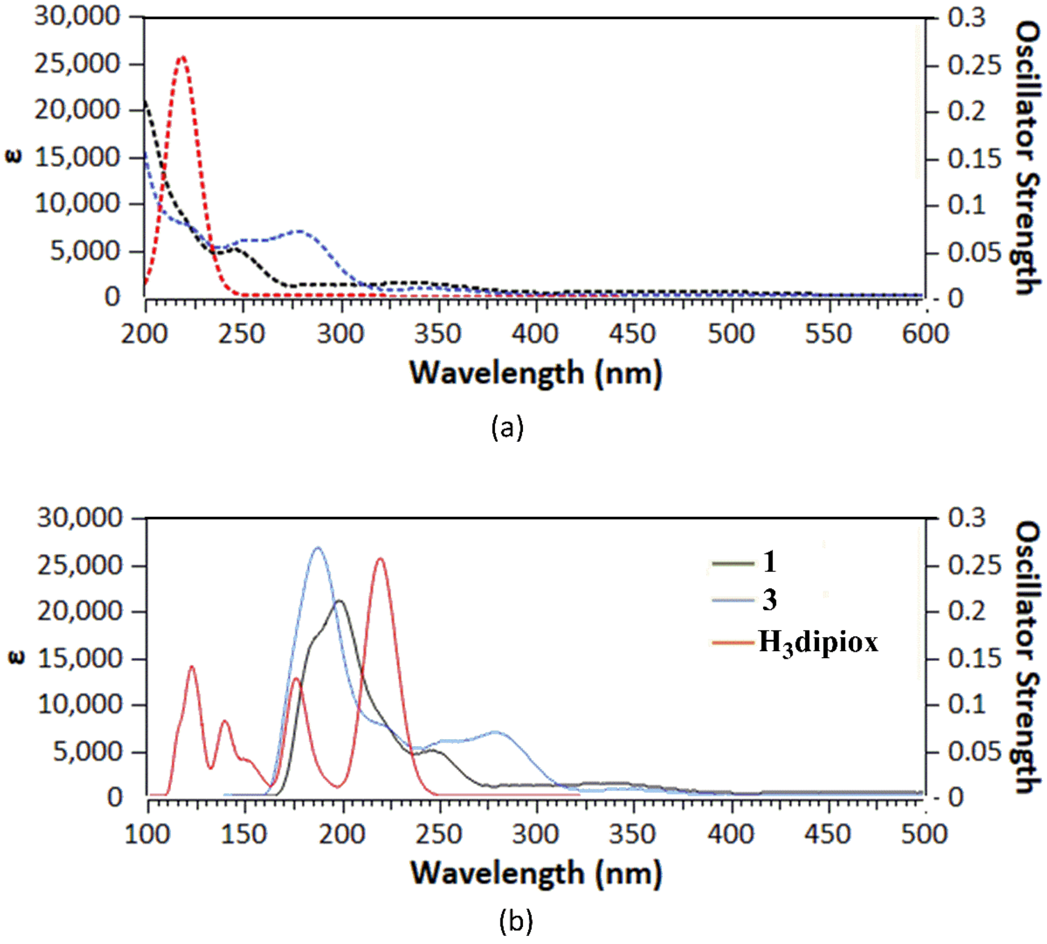

Figure S7. Simulated absorption spectra (FWHM = 0.3 eV) of **1** (black lines), **3** (blue lines) and H_3dipiox (red lines) employing (a) the wavelength scale used in experiment and (b) an extended wavelength scale.

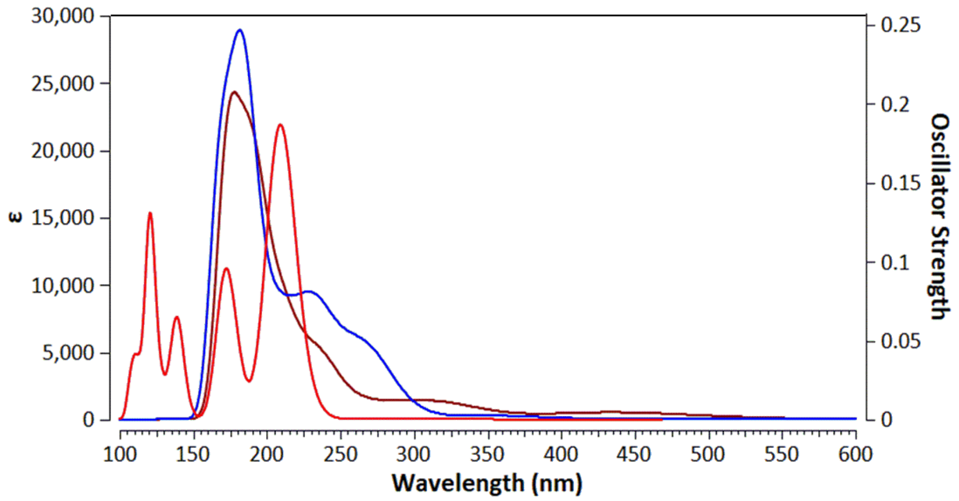

(a)

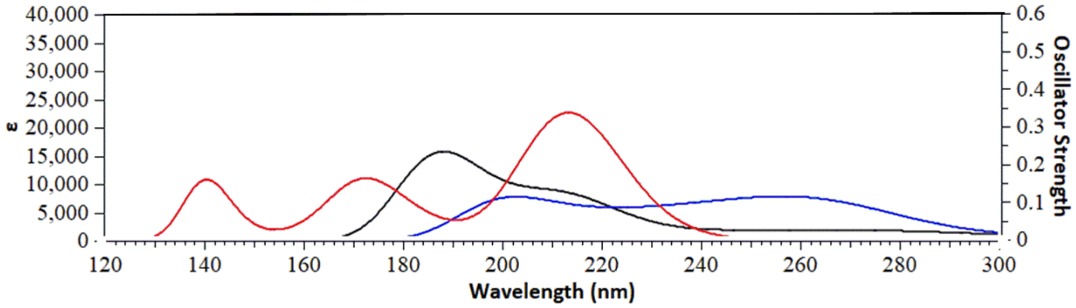

(b)

Figure S8. Simulated absorption spectra (FWHM = 0.3 eV) of **1** (black lines), **3** (blue lines) and H_3dipiox (red lines) at the (a) CAM-B3LYP/DGDZVP/PCM(Water) level and (b) CAM-B3LYP/def2-TZVPP(+D on N,O)/PCM(H_2O) level.

In order to further assist and provide a more accurate assignment of the bands appearing in the absorption spectra recorded for **1** and **3**, we set out to simulate them employing TDDFT calculations at the PBE0/DGDZVP level in water solvent. Notice that, the PBE0/DGDZVP/PCM(H_2O) protocol, was found to reproduce the experimental UV–Vis spectra most accurately among several tested methods (Table S5, Figures S7 & S8). Benchmarking against CAM-B3LYP/DGDZVP and CAM-B3LYP/Def2-TZVPP(+D on N,O) confirmed that the PBE0/DGDZVP level offers the best agreement with experiment for both band positions and intensities.

The simulated absorption spectra, at the PBE0/DGDZVP level in water solvent, show excellent agreement with those obtained experimentally (Figure S7). Accordingly, the simulated absorption spectra exhibit bands spanning the region 200 – 500 nm for both **1** and **3** complexes in line with the experimentally derived spectra (Figure 4). Specifically, the simulated spectrum of **1** exhibits an electronic transition at 224 nm in excellent agreement with the experimental spectrum which shows a maximum at 225 nm (Table 2 and Figure 4). The electronic transition at 224 nm could be described by a set of three NTO pairs depicted in Figure S9. Based upon the shape of these NTOs, this transition and consequently the experimentally found peak at 225 nm, could be assigned as basically LMCT $H_n$pidiox$^{(n-3)-}$ →Mo(VI) since all '*hole*' NTOs are mainly located on the $H_n$pidiox$^{(n-3)-}$ ligand while the respective '*particle*' NTOs are all located on the Mo metal center. It should be notice however that, this peak

has a complex nature with IL and LL′CT components as well and overall it could be assigned as LMCT/IL/LL′CT. In addition, the simulated absorption spectrum of **1**, shows an electronic transition at 249 nm in excellent agreement with the bands around 250 nm observed in the respective experimental spectrum. This electronic transition is described by a set of three NTO pairs (Figure S9). The shapes of the latter basically point towards a mainly LMCT character for the band around 250 nm. Next, the simulated absorption spectrum of **1** exhibits an electronic transition at 297 nm. The latter is in very good agreement with the peak found at 290 nm in the experimentally derived absorption spectrum (Table 2 and Figure 4). Based upon the the NTO pair that describe this electronic transition, this electronic transition could be assigned as of mainly LMCT character with minor IL/LL′CT character as well. The low energy electronic transition at 445 nm is in line with the peak at 422 nm observed in the experimentally measured absorption spectrum of **1** (Figure 4). This peak is described by a single NTO pair (Figure S9) and assigned as LMCT/LL′CT since '*hole*' is located on the $H_n$pidiox$^{(n-3)-}$ ligand while the respective '*particle*' is located on the Mo as well as on the axial O ligand. Finally, the simulated absorption spectrum of **1** shows intense bands just below 200 nm arising mainly from an electronic transition at 198 nm. The latter is described by a set of three NTO pairs (Figure S8) and based upon these it could be assigned of mainly LMCT character ($H_n$pidiox$^{(n-3)-}$ →Mo). Let's analyze now the simulated absorption spectrum of **3**, in more detail. This simulated spectrum exhibits electronic transitions peaking at 285 and 325 nm (Figure S7) in excellent agreement with those found in the experimental absorption spectrum of this complex i.e. at 280 and 326 nm (Table 2). The latter could be described by a set of two NTO pairs where the '*holes*' are mainly located on the Mo-N-O framework as well as the dipiox ligand while the '*particles*' are located on the Mo metal center and the ligands. Therefore, the lowest energy peak in the spectrum of **3** has a complex nature and assigned as LMCT/LL′CT/IL/MC. On the other hand, the high energy peak in the region 280 – 290 nm is described by a single NTO pair (Figure S9) and has also a LMCT/LL′CT/IL/MC complex assignment. In addition, the band in the region 220 – 250 nm arises due to an electronic transition at 233 nm which is described by an NTO pair (Figure S10). Based upon this NTO pair, this electronic transition and by extension the band in between 220 and 250 nm is assigned as of mainly LMCT character. Finally, the simulated absorption spectrum of **3** shows also a very intense band around 180 nm. This band is due mainly to an electronic transition at 183 nm which could be described by a set of three NTO pairs (Figure S10). Based on the latter, this band could be assign as MLCT with IL/LL′CT contributions as well.

Finally, the simulated absorption spectrum of the H_3pidiox ligand exhibits a maximum at 220 nm in line with the experimentally derived spectrum, exhibiting a band peaking around 235 nm. This electronic transition is described by an NTO pair (Figure S9) and could be assigned as $\pi^* \rightarrow \pi^*$ (Figure S10).

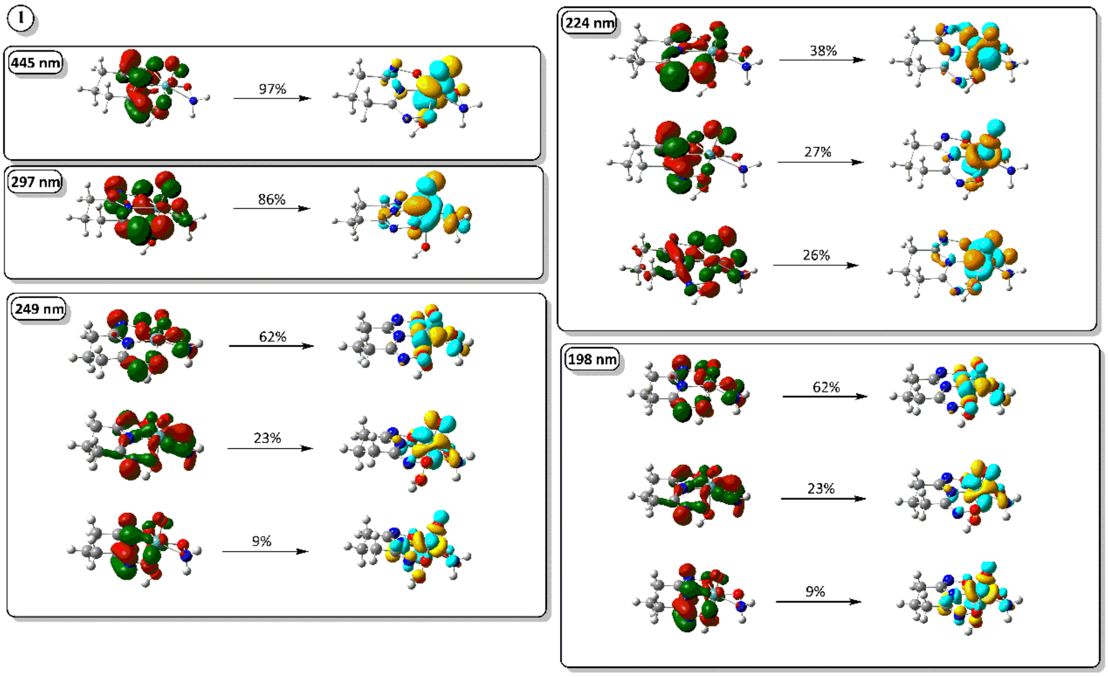

Figure S9. 3D surfaces (isocontour value set to 0.08 au) of NTO pairs (*hole* - left)/*particle* – right) corresponding to the electronic transition related to the most important absorption bands found in the simulated absorption spectra of **1**.

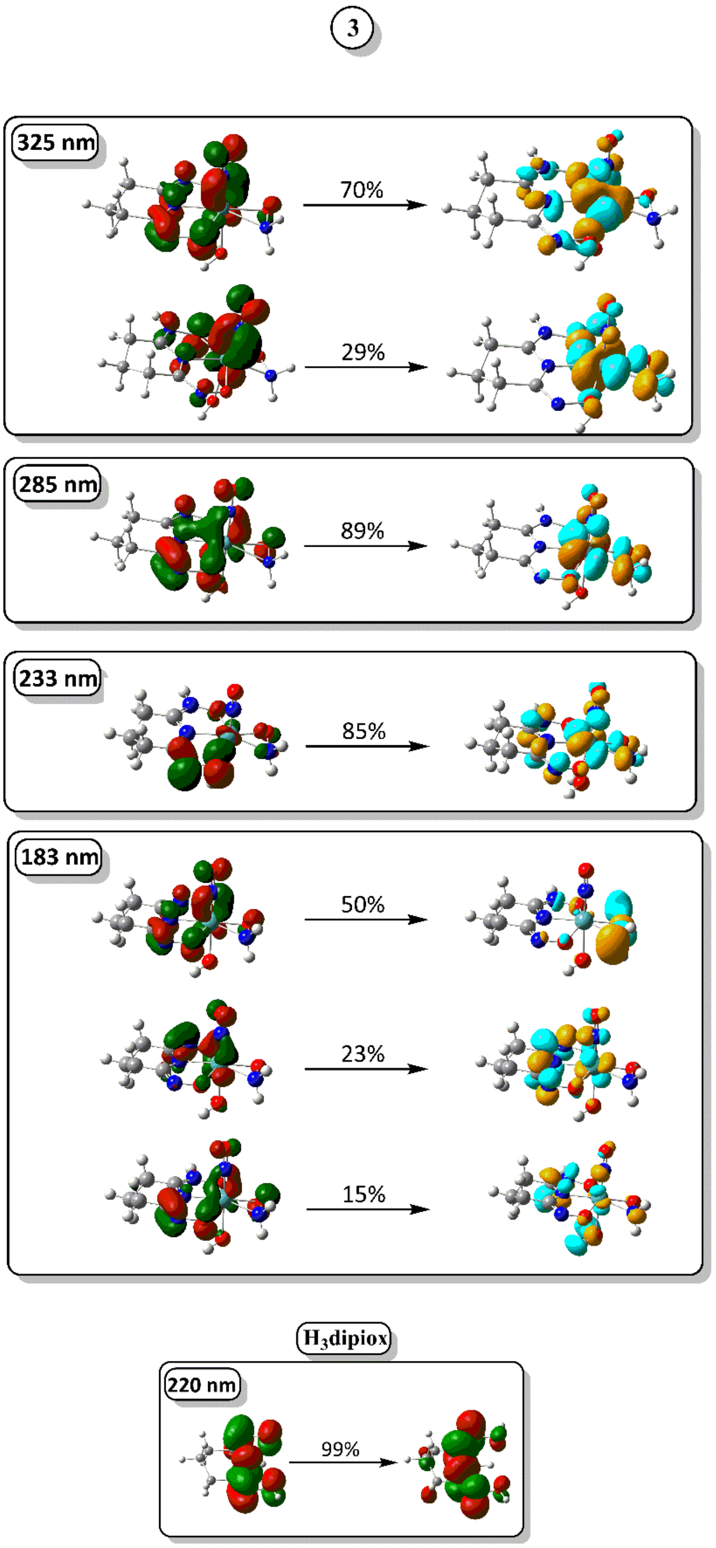

Figure S10. 3D surfaces (isocontour value set to 0.08 au) of NTO pairs (*hole* - left)/*particle* – right) corresponding to the electronic transition related to the most important absorption bands found in the simulated absorption spectra of **3** and **H_3dipiox** ligand.

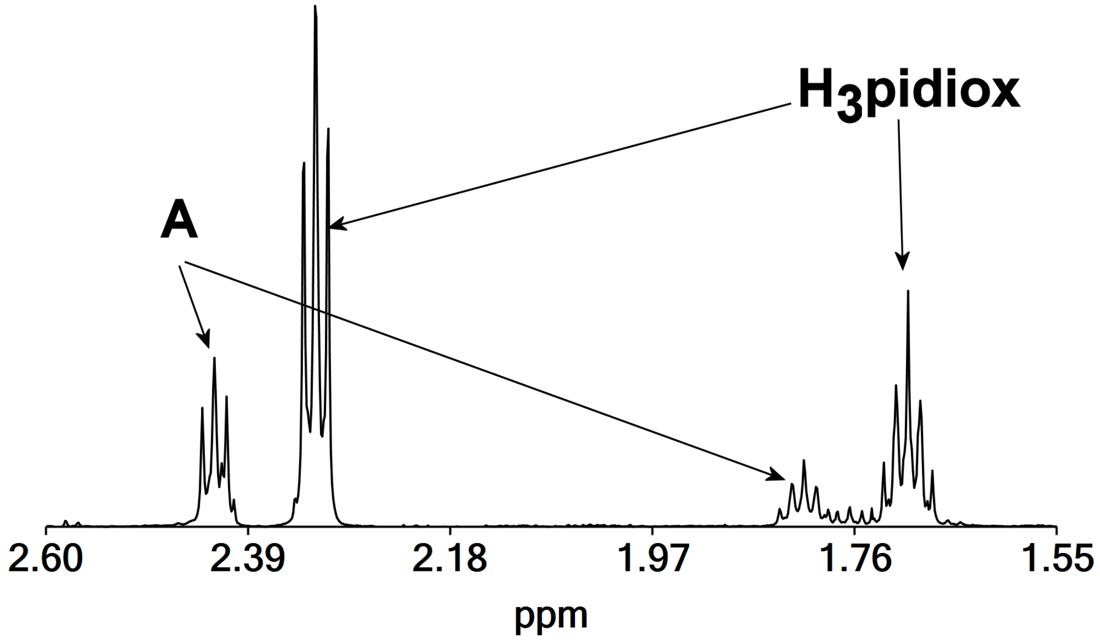

Figure S11. ^{1}H NMR spectra of H_3pidiox (13.0 mM) and Na_2MoO_4 (13.0 mM) at pD = 8.0.

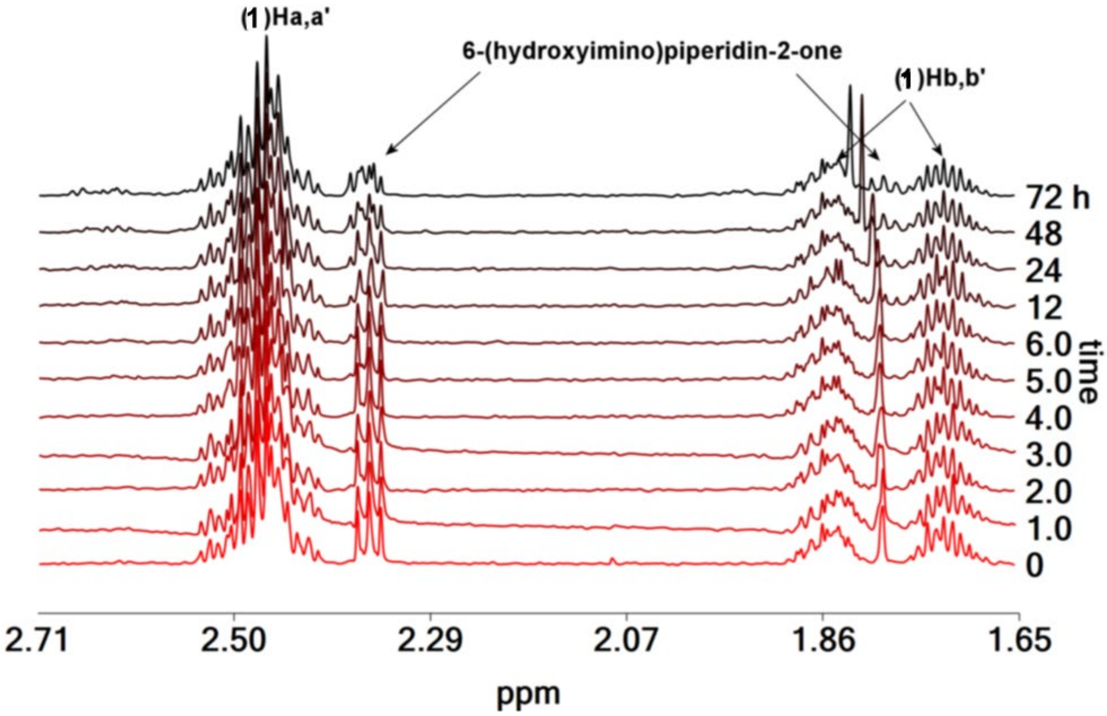

Figure S12. ^{1}H NMR spectra of D_2O solutions of complex **1** (1.3 mM) *vs* time at pD=5.6.

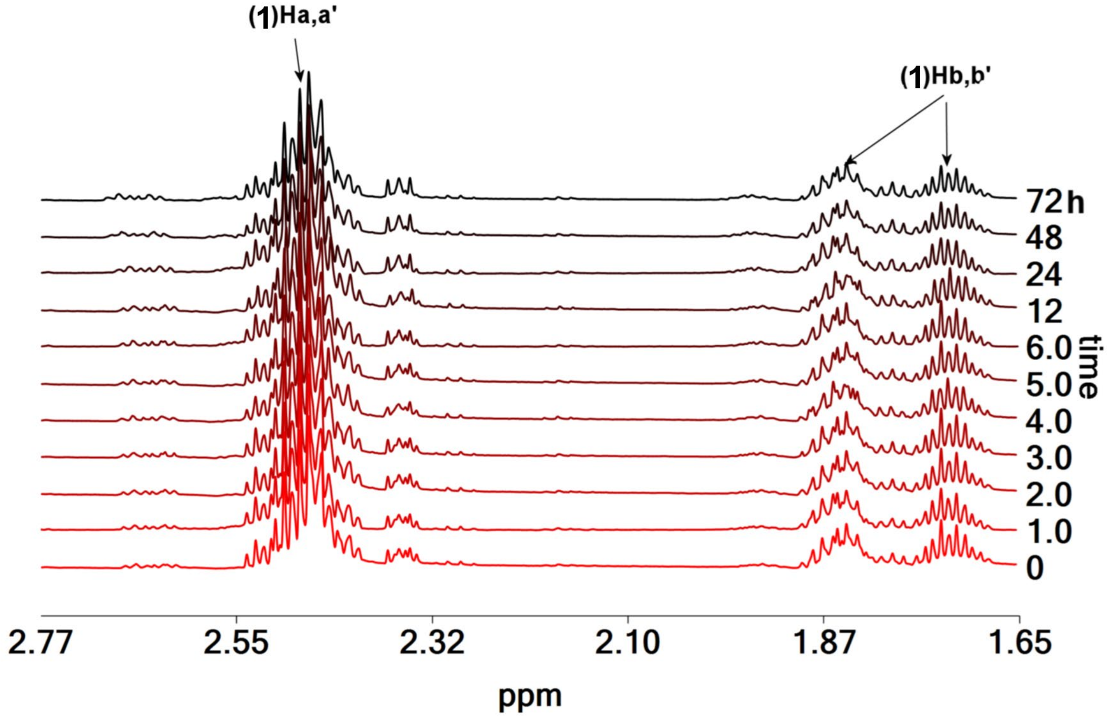

Figure S13. ^{1}H NMR spectra of D_2O solutions of complex **1** (13 mM) *vs* time at pD=5.4.

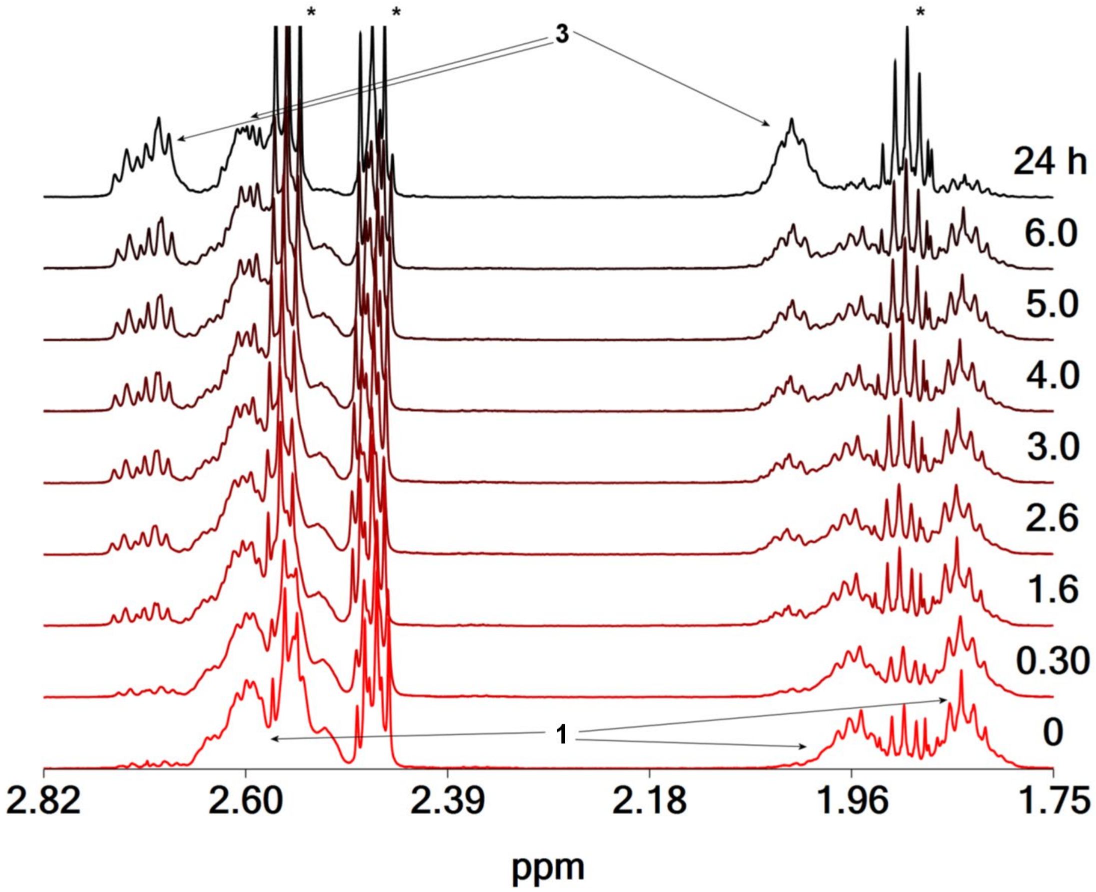

Figure S14. ^{1}H NMR spectra of D_2O solutions of complex **1** (13 mM) intense orange color *vs* time at pD=8.0 (adjusted with NaOD). * free H_3pidiox6-(hydroxyimino)piperidin-2-one and piperidine-2,6-dione.

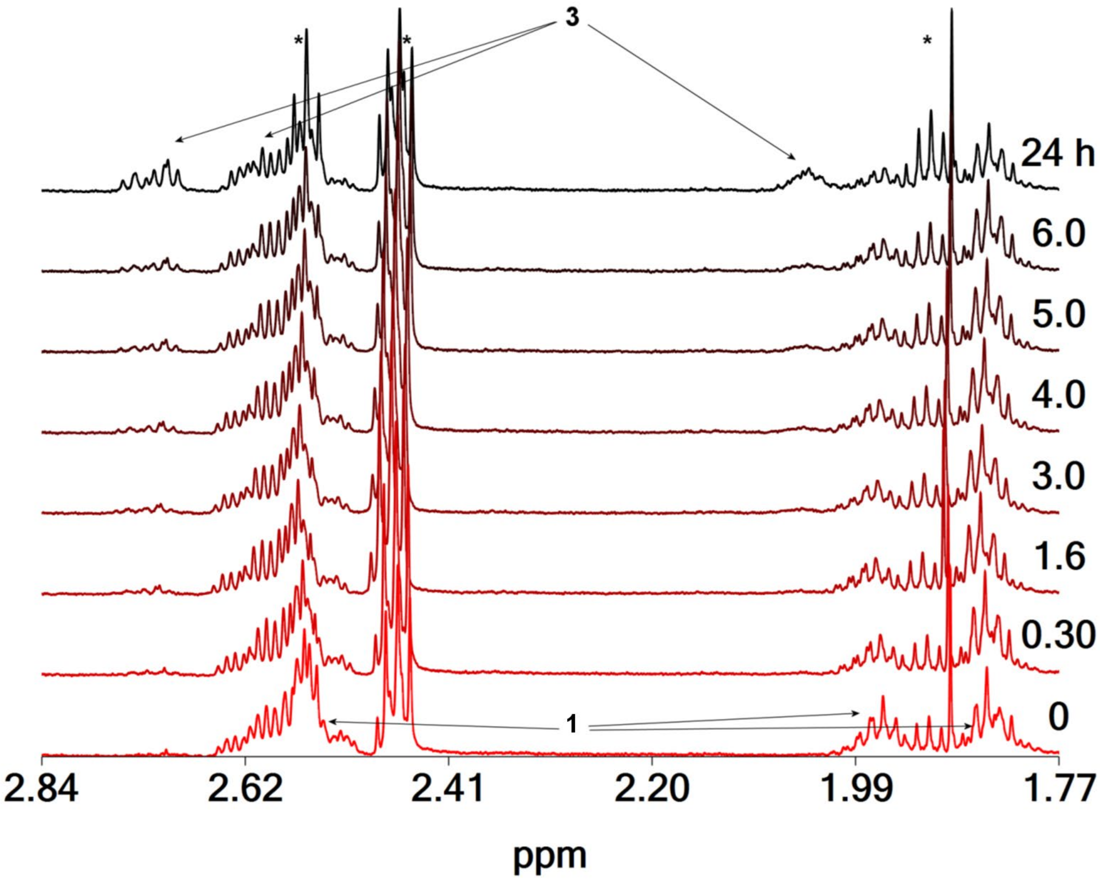

Figure S15. ^{1}H NMR spectra of D_2O solutions of complex **1** (1.3 mM) yellow orange color *vs* time at pD=8.0 (adjusted with NaOD). free H_3pidiox6-(hydroxyimino)piperidin-2-one and piperidine-2,6-dione.

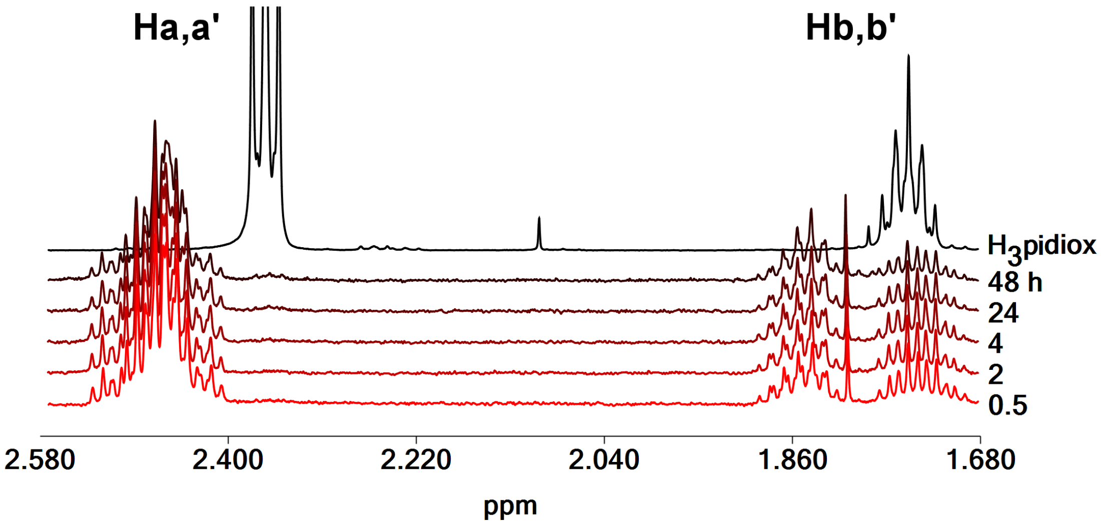

Figure S16. ^{1}H NMR spectra of the D_2O solutions of $(NH_4)_5Mo_7O_{24}$ (1.50 mM) , 10.5mM/Mo , H_3pidiox (2.10 mM) and NH_2OH (2.10 mM) at pD 6.0 *vs* time.

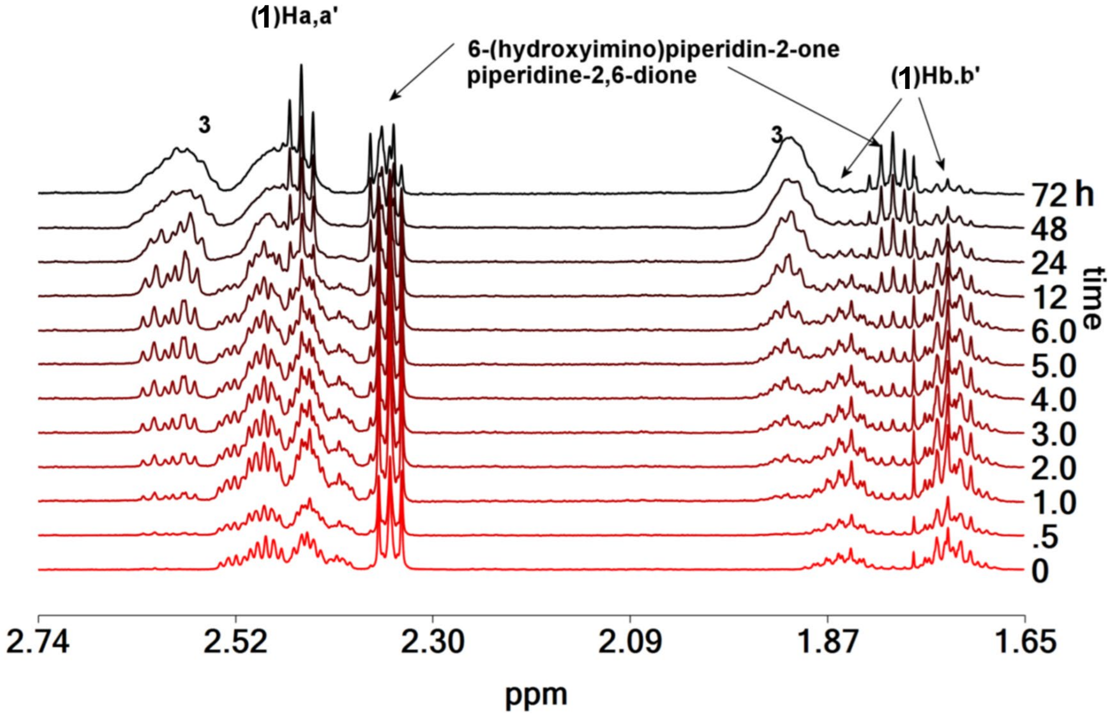

Figure S17. ^{1}H NMR spectra of D_2O solutions of $(NH_4)_5Mo_7O_{24}$ (0.75mM) , 5.25mM/Mo + H_3pidiox (10.5 mM) + NH_2OH (10.5mM) *vs* time at at pD=7.5

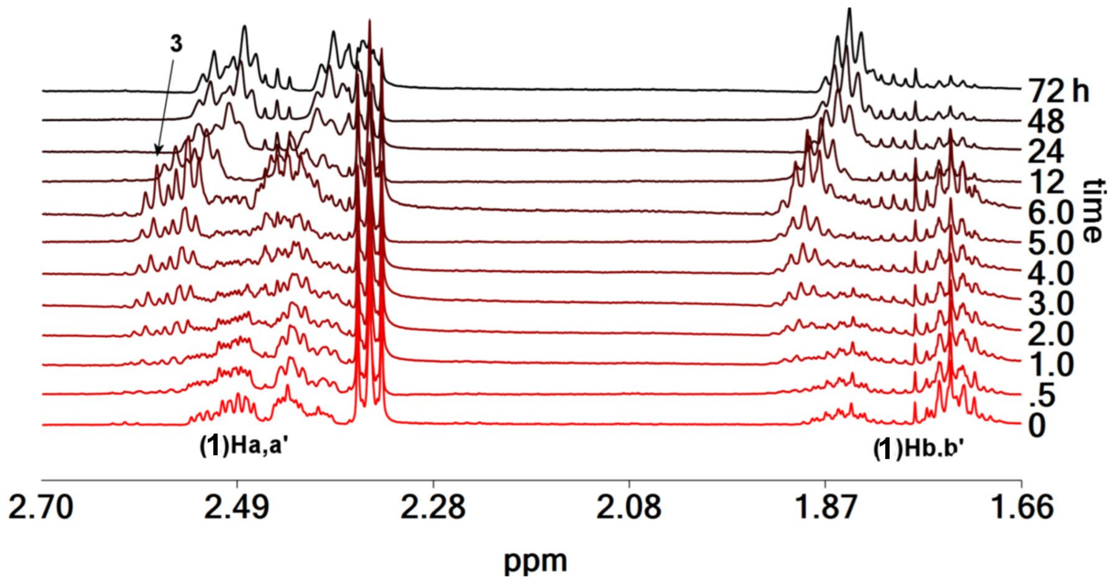

Figure S18. ^{1}H NMR spectra of D_2O solutions of $(NH_4)_5Mo_7O_{24}$ (0.75mM) , 5.25mM/Mo + H_3pidiox (10.5 mM) + NH_2OH (21.0mM) *vs* time at at pD=7.5

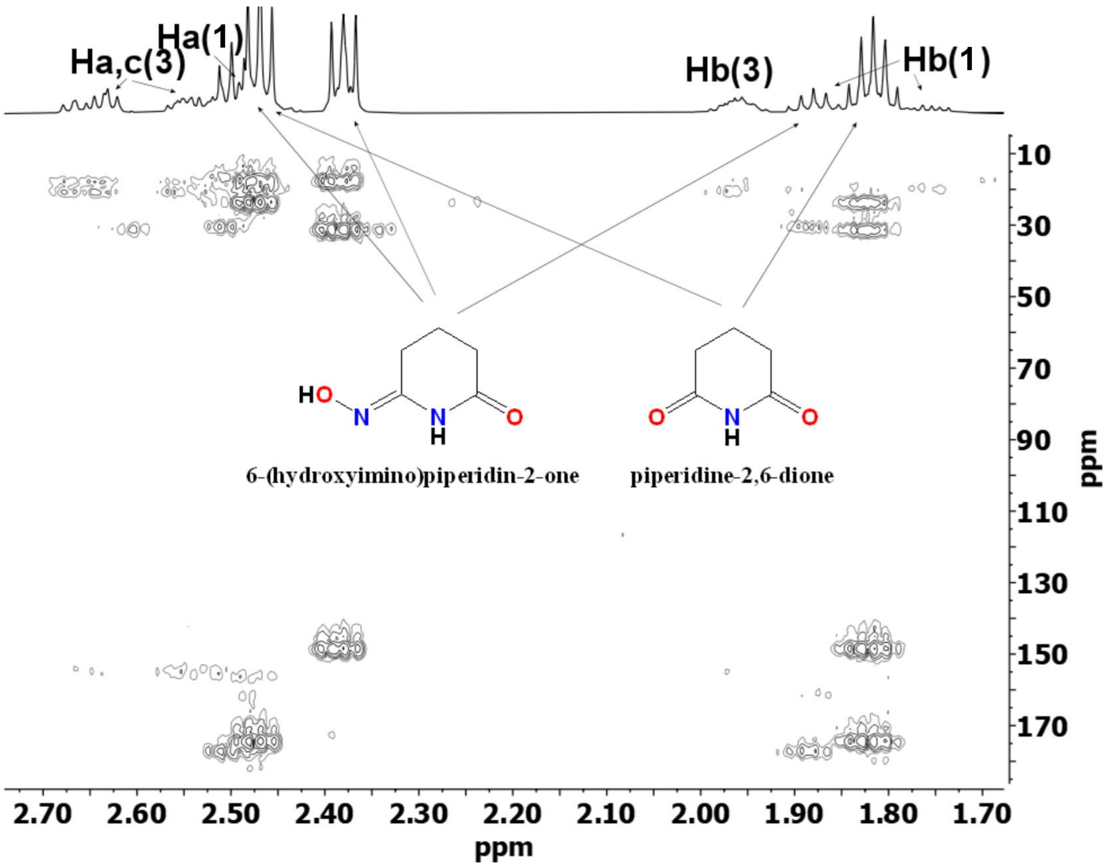

Figure S19. 2D{^{1}H,^{13}C}HMBC NMR spectra of the D_2O solutions of $(NH_4)_5Mo_7O_{24}$ (3.0 mM Mo^{VI}) and H_3pidiox (3.0 mM) at pD 6.0 after 8 days.

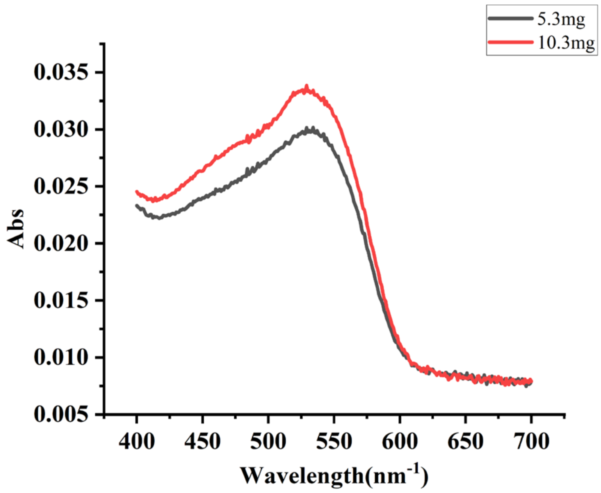

Figure S20. The UV-vis measurements of vials 3 (red line) and 4 (black line)

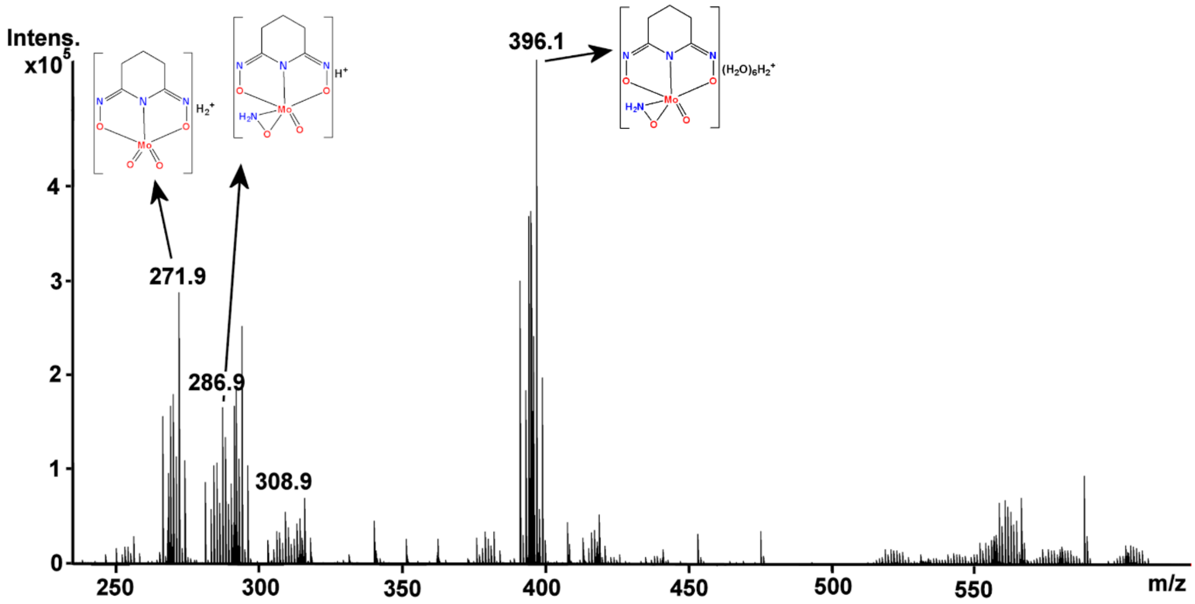

Figure S21. Positive mode ion mass spectrum of reaction mixture $(NH_4)_6Mo_7O_{24}$ (7.5mM), and H_3pidiox (105 mM) in MeOH:H_2O at t=0.

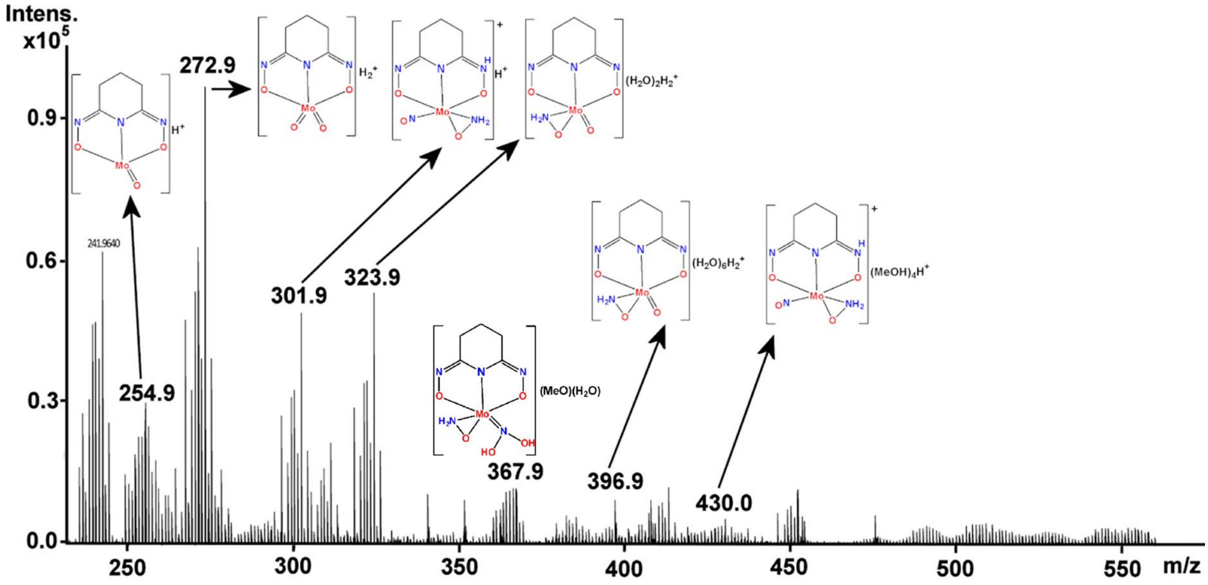

Figure S22. Positive mode ion mass spectrum of reaction mixture $(NH_4)_6Mo_7O_{24}$ (7.5mM), and H_3pidiox (105 mM) in MeOH: H2O at t=96hrs.

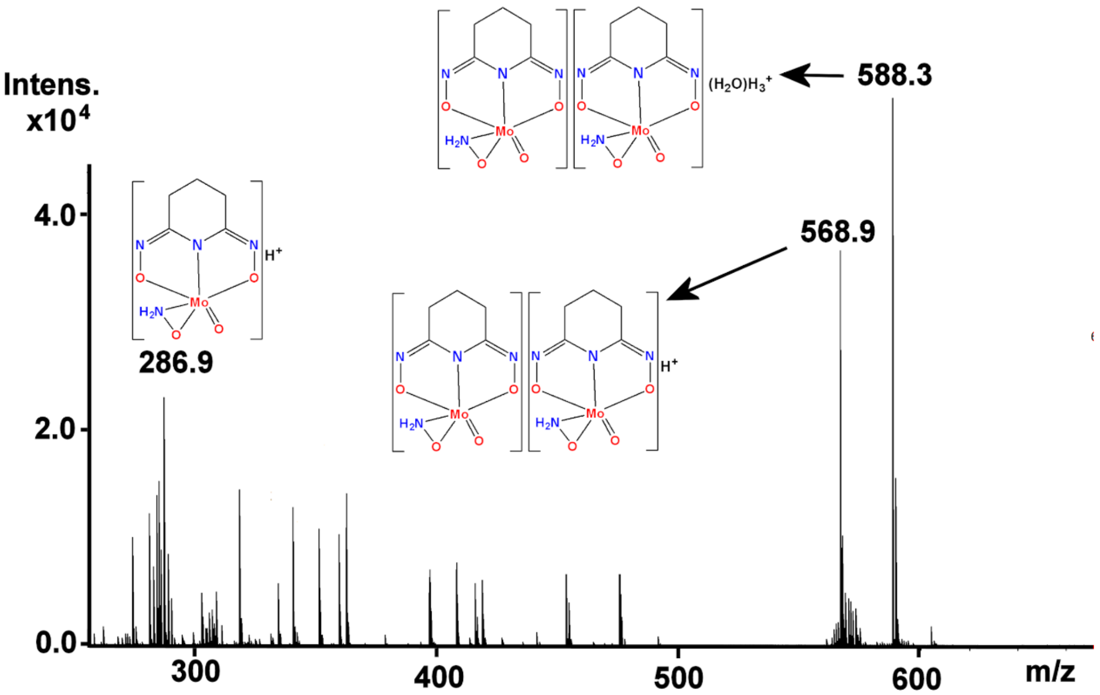

Figure S23. Positive mode ion mass spectrum of complex **1** (1.3 mM) in MeOH:H_2O at t=96hrs.

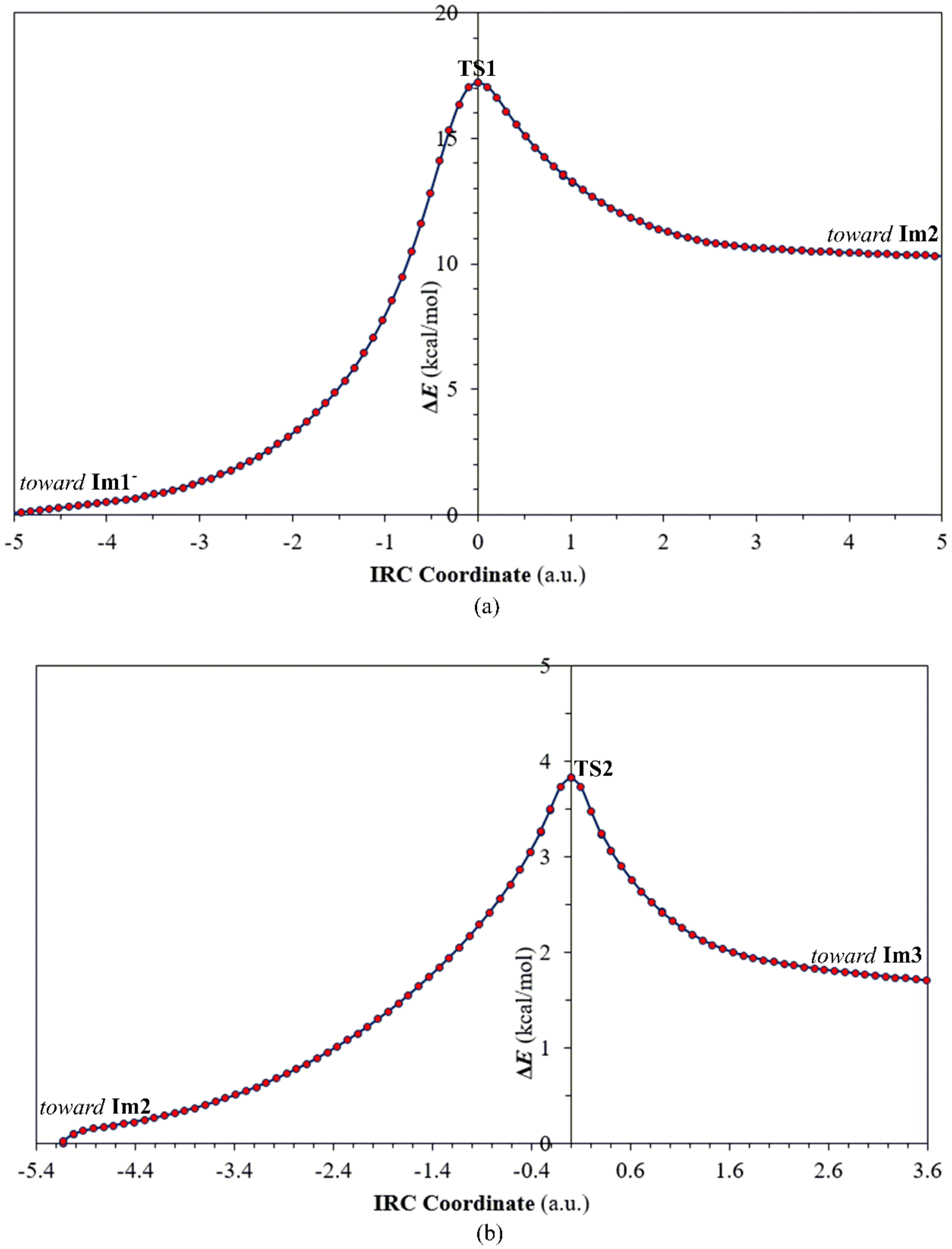

Figure S24. Intrinsic reaction coordinate (IRC) energy profiles for TS1 and TS2 computed at the PBE0/def2-TZVP/PCM(water) level of theory. Energies are expressed as relative Gibbs free energies (in kcal·mol^{-1}) with respect to the lowest-energy point along each path.

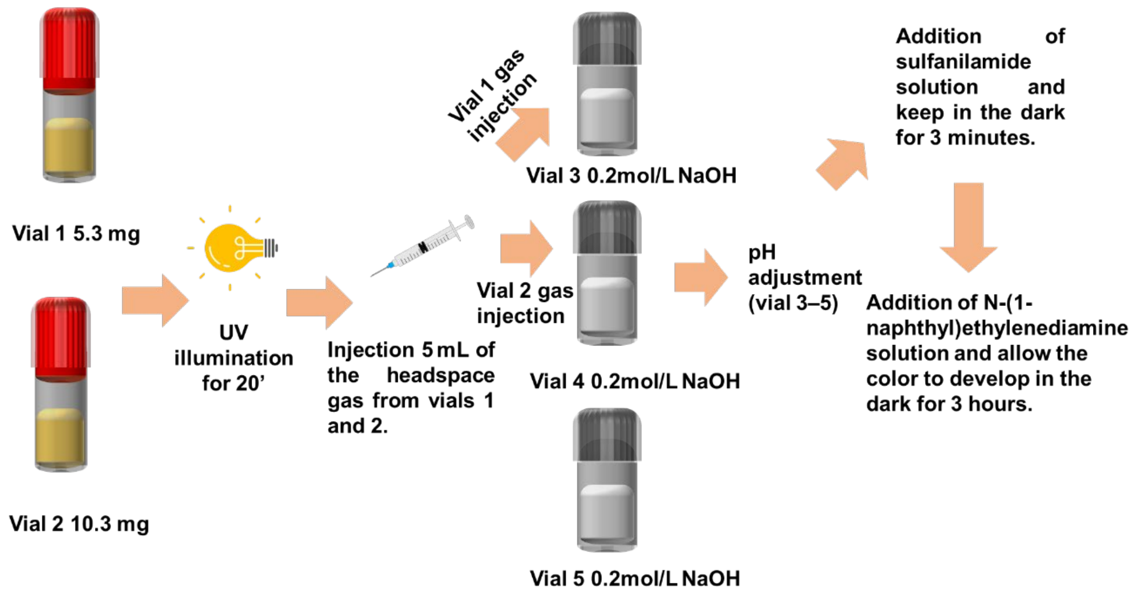

Figure S25. Photodegradation experiments detail

*Electrochemistry***.** The CVs of H_3pidiox, **1** and **3**·2H_2O were acquired in two different solvent media (H_2O and DMSO). The CVs in both solvents were similar. CVs of the DMSO solutions of **1** and **3**·2H_2O are shown in Figure S26.

The DMSO solution of H_3pidiox gave an anodic peak at 1650 mV and a corresponding cathodic peak at -1250 mV. In addition to the peaks of the ligand, the CV of **1** gave a cathodic peak at 1050 mV assigned to the reduction of Mo^{VI} metal center. The CV of complex **3**·2H_2O gave a couple of peaks at 860 (anodic) and 410 mV (cathodic) assigned to the oxidation and reduction of the Mo^{II} metal center respectively.

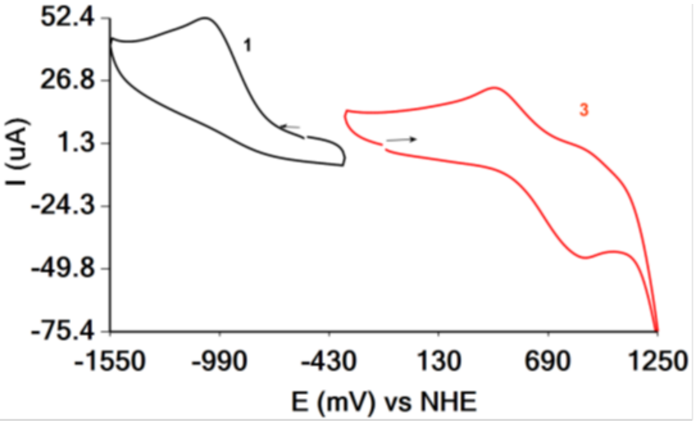

Figure S26. CVs of **1** (5.0 mM) and **3**·2H_2O (5.0 mM) in solution (DMSO). Working Electrode, glassy carbon disk, Auxiliary Electrode platinum wire, Reference Electrode, Ag/AgCl (0.2 V *vs* NHE measured with Ferrocene 0.65 V *vs* NHE).

**Table S1**. Crystal data and structure refinement for the prepared compounds.			
Compound	**1**·2CH_3OH	**2**·2CH3OH	**3**·2H_2O
Empirical formula	$C_7H_{18}MoN_4O_7$	C8H20MoN4O7	$C_5H_{15}MoN_5O_7$
Formula	[MoO(pidiox)(ONH_2)(H_2O)]·2CH_3OH	[MoO(pidiox)(ONH2)(CH3OH)]□2MeOH	[Mo(NO)(Hpidiox)(ONH_2)(H_2O)]·2H_2O
Formula weight	366.19	380.22	353.16
Temperature (K)	296(2)	150(2)	150(2)
Wavelength (Å)			
Crystal system	Triclinic	Monoclinic	Triclinic
Space group	$P\bar{1}$	$P2_1/n$	$P\bar{1}$
Unit cell dimensions *a, b, c* (Å), *α, β, γ* (°)	8.1255(4), 8.3227(4), 11.0103(6), 83.520(2), 76.822(2), 76.706(2)	8.7888(9), 11.6195(12), 14.4347(15), 90, 100.771(4), 90	6.8386(2), 8.1649(2), 11.3570(3), 106.873(2), 94.442(2), 103.075(2)
Volume (Å^3)	704.11(6)	1448.1(3)	584.09(3)
Z	2	4	2
Density (calculated) (g/cm^3)	1.727	1.744	2.008
Absorption coefficient (mm^{-1})	0.965	0.942	1.162
F(000)	372	776	356
Crystal size (mm^3)	0.35 x 0.15 x 0.1	0.12 x 0.12 x 0.05	0.12 x 0.08 x 0.04
θ range for data collection (°)	2.910 to 26.442	2.266 to 26.415	2.700 to 25.998
Index ranges	$-10 \leq h \leq 10$, $-10 \leq k \leq 10$, $-13 \leq l \leq 13$	$-10 \leq h \leq 10$, $-14 \leq k \leq 14$,	$-8 \leq h \leq 8$, $-10 \leq k \leq 10$, $-13 \leq l \leq 13$
Reflections collected	11541	$-18 \leq l \leq 18$	7048
Independent reflections	2880 [R_{int} = 0.0654]	30188	2295 [R_{int} = 0.0212]
Completeness to θ (°) °	99.6% (24.999)	2962 [R_{int} = 0.0400]	100% (25.242)
Refinement method	Full-matrix least-squares on F^2		
Data / restraints / parameters	2880 / 6 / 192	2962 / 0 / 195	2295 / 9 / 181
Goodness-of-fit	1.145	1.077	1.085
Final R indices [I > 2σ(I)]	R_{obs} = 0.0458, wR_{obs} = 0.0811	R_{obs} = 0.0218, wR_{obs} = 0.0577	R_{obs} = 0.0180, wR_{obs} = 0.0421

R indices [all data]	R_{all} = 0.0576, wR_{all} = 0.0841	R_{all} = 0.0226, wR_{all} = 0.0583	R_{all} = 0.0201, wR_{all} = 0.0427
Largest diff. peak and hole (e·Å^{-3})	0.800 and -0.613	1.462 and -0.635	0.355 and -0.330
R = Σ‖F_o|-|F_c‖ / Σ|F_o|, wR = {Σ[w(|F_o|^2 - |F_c|^2)^2] / Σ[w(|F_o|^4)]}$^{1/2}$ and w=1/[σ^2(Fo^2)+(aP)^2+bP] where P=(Fo^2+2Fc^2)/3. **1**: a = 0.0275, b = 2.1407; **2**: a = 0.0120, b = 1.2484; **3**: a = 0.0168, b = 0.4289.			

Table S2. H-bonds 1·2CH_3OH

D – H ··· A	D – H (Å)	H ··· A (Å)	D ··· A (Å)	∠ D – H ··· A (°)
O(5) – H(15) ··· O(2S)	0.84(5)	1.86(5)	2.704(6)	177(5)
O(5) – H(25) ··· N(2)#1	0.83(5)	1.89(4)	2.720(5)	178(6)
N(4) – H(14) ··· O(1S)#2	0.84(5)	2.07(5)	2.887(5)	163(6)
N(4) – H(24) ··· O(1S)#3	0.83(6)	2.02(6)	2.843(5)	170(5)
O(1S) – H(1S) ··· N(3)	0.85(5)	1.95(5)	2.770(5)	164(4)
		1.95(4)	2.750(5)	153(3)
O(2S) – H(2S) ··· O(2)#2	0.86(5)			

Symmetry transformation used to generate equivalent atoms: #1, -x+1, -y+1, -z+2; #2, -x+1, -y+1, -z+1; #3, x+1, y, z.

Table S3. H-bondsof **2·2CH_3OH**

D – H ··· A	D – H (Å)	H ··· A (Å)	D ··· A (Å)	∠ D – H ··· A (°)
O(7) – H(5) ··· O(2)	0.84	1.94	2.762(2)	167
O(8) – H(6) ··· O(1)	0.84	1.87	2.695(2)	166
N(4) – H(9A) ··· O(7)#1	0.90(3)	1.88(3)	2.769(2)	171(3)
N(4) – H(9B) ··· O(8)#2	0.88(3)	1.92(3)	2.787(2)	169(3)
O(5) – H(10) ··· N(3)#3	0.74(3)	2.04(5)	2.771(2)	173(3)

Symmetry transformation used to generate equivalent atoms: #1, -x+1/2, y-1/2, -z+1/2; #2, -x+1, -y+1, -z+1; #3, -x+1, -y+2, -z+1.

Table S4. H-bonds **3**·2H_2O

D – H ··· A	D – H (Å)	H ··· A (Å)	D ··· A (Å)	∠ D – H ··· A (°)

O(1S) – H(1SA) ··· O(1)	0.83(3)	1.87(3)	2.698(2)	175(3)
O(1S) – H(1SB) ··· O(1)#1	0.81(3)	2.39(2)	3.142(2)	154(3)
O(1S) – H(1SB) ··· O(5)#1	0.81(3)	2.42(3)	3.032(3)	133(2)
N(3) – H(3) ··· O(3)#2	0.88	1.95	2.819(2)	171
O(2S) – H(2SA) ··· O(2)#3	0.82(2)	1.93(2)	2.735(2)	167(2)
O(2S) – H(2SB) ··· N(2)#4	0.79(2)	2.03(3)	2.809(2)	170(3)
O(5) – H(5A) ··· O(1S)#2	0.83(3)	1.79(3)	2.621(3)	176(2)
O(5) – H(5B) ··· O(4)#5	0.80(2)	1.94(2)	2.735(2)	175(3)
N(4) – H(12A) ··· O(2S)#6	0.91	1.97	2.820(2)	156
N(4) – H(12B) ··· O(2S)#1	0.91	2.00	2.852(2)	156

Symmetry transformation used to generate equivalent atoms: #1, -x+2, -y+1, -z; #2, x-1, y, z; #3, x+1, y, z; #4, x, y-1, z; #5, -x+1, -y, -z; #6, -x+2, -y, -z.

Table S5. Comparison of Experimental and TDDFT-Computed UV–Vis Absorption Maxima (in nm) for the Reactant, **1** Product, **3** and H_3dipiox Ligand.

Compound	Experiment	PBE0/DGDZVP	CAM-B3YP/DGDZVP	CAM-B3YP/Def2-TZVPP(+D)
Reactant, **1**	225, 422	224, 445	211, 237, 420	214, 384
Product, **3**	280, 326	285, 325	271, 352	266, 334
H_3dipiox	235	220	209	213

Table S6. Cartesian coordinates and energetic data of the optimized geometries of all species involved in the reaction mechanism of **1** to **3** conversion calculated at the PBE0/Def2-TZVP level in water solvent (gas phase calculations for some species were also performed as indicated in the species legends).

Reactant, 1

Mo	-1.035825000	0.022052000	-0.303991000
O	-0.454373000	1.861171000	0.061165000
N	0.969797000	0.010371000	-0.248173000
C	1.621218000	1.206330000	-0.091564000
N	0.875846000	2.235976000	0.062328000
O	-0.486487000	-1.836661000	-0.008669000
C	3.112624000	1.242530000	-0.094726000
N	0.842902000	-2.230079000	-0.037854000
O	-1.321211000	0.027243000	-1.955778000
C	3.675631000	-0.037228000	0.518141000
O	-0.940399000	-0.168530000	2.171343000
N	-2.947555000	-0.567076000	0.363435000
C	3.091451000	-1.265269000	-0.175991000
C	1.601173000	-1.206956000	-0.157629000
O	-2.729292000	0.781417000	0.389705000
H	3.452851000	2.132149000	0.435057000
H	3.455237000	1.327610000	-1.132257000
H	4.762362000	-0.043460000	0.423676000
H	3.443340000	-0.070272000	1.586841000
H	-0.263183000	-0.758785000	2.518025000
H	-0.819271000	0.677646000	2.615618000
H	-3.028991000	-0.969158000	1.294110000
H	-3.690159000	-0.841999000	-0.273836000
H	3.425583000	-1.284420000	-1.219626000
H	3.421442000	-2.193394000	0.290343000

Sum of electronic and zero-point Energies= -859.221660
Sum of electronic and thermal Energies= -859.206594
Sum of electronic and thermal Enthalpies= -859.205650
Sum of electronic and thermal Free Energies= -859.262712

Im1

Mo	1.128155000	-0.216549000	-0.062741000
O	0.571086000	1.388652000	-1.110121000
N	-0.952961000	-0.098327000	-0.034356000
C	-1.541579000	0.813591000	-0.853301000
N	-0.752977000	1.590076000	-1.508415000
O	0.466031000	-1.308527000	1.403651000
C	-3.034560000	0.883172000	-0.931837000
N	-0.870354000	-1.524321000	1.693394000
O	2.759920000	0.144289000	0.277122000
C	-3.658872000	-0.410124000	-0.412265000
N	1.551521000	-2.017109000	-1.070993000
C	-3.101721000	-0.773144000	0.963733000
C	-1.610178000	-0.822892000	0.901537000
O	1.167798000	-0.975177000	-1.870134000
N	0.879704000	1.408019000	1.584854000
O	0.935819000	2.723859000	1.114764000
H	-3.340054000	1.096175000	-1.957146000
H	-3.369843000	1.725954000	-0.317291000

H	-4.741951000	-0.295270000	-0.352789000
H	-3.459176000	-1.225301000	-1.114156000
H	0.892707000	-2.791100000	-1.077782000
H	2.512987000	-2.306304000	-1.231960000
H	-3.398749000	-0.011900000	1.693893000
H	-3.487771000	-1.728843000	1.320084000
H	0.015145000	1.305027000	2.111647000
H	1.654977000	1.297892000	2.233550000
H	0.830899000	2.591946000	0.146214000

Sum of electronic and zero-point Energies= -914.442123
Sum of electronic and thermal Energies= -914.426456
Sum of electronic and thermal Enthalpies= -914.425512
Sum of electronic and thermal Free Energies= -914.485070

TS1, Neutral

Mo	1.112124000	0.034791000	-0.041396000
O	0.513833000	1.910106000	0.023259000
N	-0.894049000	0.068146000	-0.337520000
C	-1.557316000	1.242506000	-0.066081000
N	-0.809882000	2.261572000	0.135440000
O	0.548957000	-1.736183000	-0.543684000
C	-3.049303000	1.265751000	-0.035172000
N	-0.760152000	-2.153724000	-0.593774000
O	2.439571000	-0.118260000	1.219839000
C	-3.613925000	0.138802000	-0.894439000
N	2.694658000	-0.394726000	-1.388303000
C	-3.018640000	-1.199658000	-0.469758000
C	-1.528279000	-1.127988000	-0.468225000
O	2.219714000	0.879524000	-1.428585000
N	0.336385000	-0.041822000	2.030437000
O	-0.726666000	-0.867033000	2.419410000
H	-3.403509000	2.244210000	-0.360452000
H	-3.376519000	1.129384000	1.001780000
H	-4.699368000	0.107176000	-0.793901000
H	-3.391847000	0.327032000	-1.948926000
H	2.531087000	-0.913360000	-2.247163000
H	3.645661000	-0.456686000	-1.035104000
H	-3.349696000	-1.442677000	0.545798000
H	-3.340310000	-2.014662000	-1.118853000
H	1.645848000	-0.212313000	2.065917000
H	0.053453000	0.887160000	2.332602000
H	-0.351208000	-1.754339000	2.453127000

Sum of electronic and zero-point Energies= -914.400024
Sum of electronic and thermal Energies= -914.384757
Sum of electronic and thermal Enthalpies= -914.383813
Sum of electronic and thermal Free Energies= -914.441805

TS1, Neutral (Gas phase)

Mo	1.114679000	0.049774000	-0.033835000
O	0.508416000	1.902867000	0.133418000
N	-0.887264000	0.086102000	-0.353298000
C	-1.553134000	1.248210000	-0.022924000
N	-0.808904000	2.252917000	0.239176000
O	0.555287000	-1.709130000	-0.574439000
C	-3.045695000	1.268431000	0.003966000
N	-0.750060000	-2.132661000	-0.648732000
O	2.442976000	-0.151297000	1.204638000

C	-3.606150000	0.168742000	-0.893373000
N	2.682362000	-0.342039000	-1.416263000
C	-3.008020000	-1.182148000	-0.511508000
C	-1.516922000	-1.107654000	-0.508819000
O	2.156795000	0.915265000	-1.464092000
N	0.346618000	-0.149020000	2.030886000
O	-0.723579000	-0.988334000	2.380017000
H	-3.396889000	2.258406000	-0.288794000
H	-3.378114000	1.098325000	1.034697000
H	-4.693246000	0.132000000	-0.802415000
H	-3.379190000	0.392255000	-1.940468000
H	2.519267000	-0.883161000	-2.260186000
H	3.638019000	-0.360884000	-1.071884000
H	-3.336651000	-1.456066000	0.497320000
H	-3.326412000	-1.980403000	-1.183095000
H	1.654965000	-0.309486000	2.056897000
H	0.071090000	0.760312000	2.392583000
H	-0.408254000	-1.877358000	2.183640000

Sum of electronic and zero-point Energies= -914.376429
Sum of electronic and thermal Energies= -914.361270
Sum of electronic and thermal Enthalpies= -914.360326
Sum of electronic and thermal Free Energies= -914.417885

TS1 (Reduced form)

Mo	1.135302000	0.105493000	0.020815000
O	0.502415000	1.645212000	1.104296000
N	-0.924974000	0.171388000	-0.122972000
C	-1.574506000	1.051108000	0.682127000
N	-0.843481000	1.857373000	1.371768000
O	0.598893000	-1.221047000	-1.343682000
C	-3.069690000	1.027128000	0.734143000
N	-0.734320000	-1.586465000	-1.530779000
O	2.648012000	-0.522180000	0.919874000
C	-3.630254000	0.458985000	-0.568938000
N	2.443969000	0.853734000	-1.476397000
C	-3.005610000	-0.899633000	-0.883012000
C	-1.512077000	-0.791924000	-0.869938000
O	1.223965000	1.479675000	-1.516075000
N	0.587885000	-1.206905000	1.720123000
O	-0.001797000	-2.474812000	1.465475000
H	-3.451124000	2.029582000	0.933211000
H	-3.386032000	0.390376000	1.568846000
H	-4.714327000	0.357491000	-0.492718000
H	-3.429208000	1.154028000	-1.390295000
H	2.630809000	0.310565000	-2.315225000
H	3.208989000	1.486755000	-1.263681000
H	-3.315127000	-1.622721000	-0.119339000
H	-3.341021000	-1.286797000	-1.845989000
H	1.877292000	-1.075511000	1.628320000
H	0.022459000	-0.759615000	2.436537000
H	-0.508034000	-2.367483000	0.648504000

Sum of electronic and zero-point Energies= -914.539915
Sum of electronic and thermal Energies= -914.523958
Sum of electronic and thermal Enthalpies= -914.523013
Sum of electronic and thermal Free Energies= -914.583567

TS1 (Reduced form in gas phase)

Mo	1.130560000	-0.121215000	-0.023881000
O	0.471929000	-1.734678000	-0.975444000
N	-0.928972000	-0.146725000	0.122695000
C	-1.583799000	-1.085594000	-0.607459000
N	-0.864525000	-1.955372000	-1.225722000
O	0.601723000	1.329466000	1.236225000
C	-3.079071000	-1.051959000	-0.657883000
N	-0.726360000	1.690286000	1.431387000
O	2.653114000	0.400726000	-0.943860000
C	-3.626666000	-0.404946000	0.615144000
N	2.425959000	-0.646224000	1.576352000
C	-2.995573000	0.969775000	0.838491000
C	-1.501519000	0.858372000	0.820946000
O	1.269196000	-1.377839000	1.621061000
N	0.624953000	1.066664000	-1.822690000
O	-0.045824000	2.317592000	-1.685794000
H	-3.464006000	-2.063685000	-0.799033000
H	-3.401030000	-0.458787000	-1.523992000
H	-4.715145000	-0.311610000	0.552263000
H	-3.406176000	-1.049382000	1.472953000
H	2.510850000	0.002025000	2.354882000
H	3.258429000	-1.208295000	1.432890000
H	-3.310420000	1.642821000	0.030864000
H	-3.318330000	1.420273000	1.778954000
H	1.911748000	0.936834000	-1.706659000
H	0.081693000	0.536370000	-2.496957000
H	-0.229835000	2.388044000	-0.740272000

Sum of electronic and zero-point Energies= -914.446146
Sum of electronic and thermal Energies= -914.430186
Sum of electronic and thermal Enthalpies= -914.429242
Sum of electronic and thermal Free Energies= -914.489836

Im2

Mo	0.772935000	-0.437100000	-0.231475000
O	0.240155000	1.095076000	-1.524992000
N	-1.177988000	-0.010582000	0.114025000
C	-1.679954000	1.142796000	-0.438774000
N	-0.920087000	1.781004000	-1.262477000
O	-0.116767000	-2.142095000	0.163424000
C	-3.078439000	1.548216000	-0.111284000
N	-1.440933000	-2.206901000	0.590071000
O	3.209284000	1.302885000	0.834799000
C	-3.977897000	0.309621000	-0.108983000
N	2.684353000	-1.355719000	0.079745000
C	-3.420103000	-0.771178000	0.818167000
C	-1.980426000	-1.036154000	0.509325000
O	2.539202000	-0.885603000	-1.213366000
N	1.844105000	1.390596000	0.559033000
O	1.166644000	1.796684000	1.707513000
H	-3.427331000	2.292500000	-0.828634000
H	-3.107474000	2.009579000	0.884229000
H	3.484694000	2.176751000	1.149187000
H	-4.988207000	0.580574000	0.205678000
H	-4.052332000	-0.084794000	-1.127745000
H	2.772958000	-2.367161000	0.097346000

H	3.463856000	-0.912040000	0.555487000
H	-3.498387000	-0.423543000	1.855659000
H	-3.992880000	-1.696740000	0.745208000
H	1.713104000	2.156721000	-0.103206000
H	0.755334000	0.942193000	1.976220000

Sum of electronic and zero-point Energies= -914.453436
Sum of electronic and thermal Energies= -914.437248
Sum of electronic and thermal Enthalpies= -914.436304
Sum of electronic and thermal Free Energies= -914.497969

TS2

Mo	-0.811054000	-0.238294000	0.205235000
O	-0.073322000	0.797621000	1.859942000
N	1.229152000	0.058345000	-0.087794000
C	1.853311000	0.933195000	0.781837000
N	1.175106000	1.318923000	1.804063000
O	-0.202752000	-1.621010000	-1.183522000
C	3.269626000	1.313858000	0.504411000
O	-3.744222000	1.230125000	-1.518630000
C	4.018086000	0.114498000	-0.079836000
N	-2.828861000	-0.854309000	0.118605000
C	3.305594000	-0.432342000	-1.316709000
C	1.850268000	-0.589756000	-1.035421000
O	-2.453279000	-0.372294000	1.360302000
N	-1.358667000	1.185260000	-0.820095000
O	-0.683845000	2.469429000	-0.894155000
N	1.033395000	-1.372815000	-1.764805000
H	3.742941000	1.672662000	1.418995000
H	3.293141000	2.136558000	-0.220599000
H	-4.185878000	1.958775000	-1.075489000
H	-2.540379000	1.315232000	-1.246963000
H	5.037533000	0.401270000	-0.342886000
H	4.088682000	-0.673710000	0.675677000
H	-3.137240000	-1.818656000	0.176785000
H	-3.479713000	-0.215015000	-0.371730000
H	3.435520000	0.256768000	-2.159730000
H	3.734934000	-1.388418000	-1.626763000
H	0.059721000	2.384357000	-0.280456000
H	1.415899000	-2.181234000	-2.237363000
O	-0.172721000	-2.407495000	1.428075000
H	0.682958000	-2.299373000	1.855955000
H	0.025087000	-2.696410000	0.523092000

Sum of electronic and zero-point Energies= -991.484851
Sum of electronic and thermal Energies= -991.465045
Sum of electronic and thermal Enthalpies= -991.464101
Sum of electronic and thermal Free Energies= -991.531520

Im3

Mo	-1.099521000	0.095851000	-0.144097000
O	-0.440613000	-1.756670000	-0.844012000
N	0.978244000	0.056564000	-0.023471000
C	1.590777000	-1.165593000	-0.225562000
N	0.847827000	-2.119704000	-0.667947000
O	-0.482221000	2.022884000	-0.156601000
C	3.064707000	-1.260222000	-0.016226000
C	3.731208000	0.023283000	-0.516186000
N	-3.160540000	0.631604000	-0.347041000

C	3.124812000	1.256915000	0.153160000
C	1.636723000	1.177088000	0.121698000
O	-2.800401000	-0.505888000	-1.050889000
N	-1.475668000	-0.260757000	1.585841000
O	-0.621895000	-1.203610000	2.378111000
N	0.826629000	2.247423000	0.272539000
H	3.456032000	-2.139011000	-0.530113000
H	3.280215000	-1.381621000	1.052353000
H	4.804335000	-0.009625000	-0.320096000
H	3.602757000	0.099128000	-1.600174000
H	-3.525723000	1.344762000	-0.969397000
H	-3.826092000	0.406829000	0.390709000
H	3.464294000	1.320501000	1.194449000
H	3.462423000	2.173309000	-0.338652000
H	0.040174000	-1.518245000	1.743954000
H	1.177709000	3.149773000	-0.024592000

Sum of electronic and zero-point Energies= -838.741912
Sum of electronic and thermal Energies= -838.726932
Sum of electronic and thermal Enthalpies= -838.725988
Sum of electronic and thermal Free Energies= -838.783285

Im4

Mo	-1.125283000	-0.045699000	-0.190840000
O	-0.400781000	-1.903684000	0.353498000
N	1.107124000	0.083684000	0.002635000
C	1.670643000	-1.112789000	0.368876000
N	0.932969000	-2.148896000	0.558730000
O	-0.934776000	0.765297000	-1.751229000
C	3.146324000	-1.266989000	0.568567000
C	3.873686000	-0.432042000	-0.464998000
N	-3.324088000	-0.103936000	-0.590961000
C	3.412271000	0.998335000	-0.284282000
C	1.899126000	1.175203000	-0.185907000
O	-2.765317000	-1.284926000	-0.115408000
N	-1.384337000	0.971453000	1.209413000
O	-1.569198000	1.928849000	1.993363000
N	1.405855000	2.367251000	-0.281555000
H	3.397127000	-2.327273000	0.515644000
H	3.427384000	-0.912557000	1.569164000
H	4.957949000	-0.505901000	-0.348554000
H	3.629299000	-0.790322000	-1.470818000
H	-3.707098000	-0.234462000	-1.523006000
H	-4.041825000	0.234902000	0.042985000
H	3.848196000	1.400725000	0.637770000
H	3.764098000	1.638826000	-1.097276000
H	2.172357000	3.013945000	-0.453798000

Sum of electronic and zero-point Energies= -838.364008
Sum of electronic and thermal Energies= -838.348863
Sum of electronic and thermal Enthalpies= -838.347919
Sum of electronic and thermal Free Energies= -838.406557

Im5

Mo	-1.061698000	-0.004333000	-0.173886000
O	-0.405216000	-1.874068000	-0.591930000
N	1.011156000	-0.000676000	-0.063132000
C	1.635179000	-1.225836000	-0.073535000
N	0.891097000	-2.235852000	-0.336248000

O	-0.454458000	1.961964000	-0.391929000
C	3.102399000	-1.283576000	0.189419000
C	3.779740000	-0.031296000	-0.367886000
N	-3.018664000	0.615788000	-0.652872000
C	3.124534000	1.240972000	0.171697000
C	1.648111000	1.154846000	-0.008269000
O	-2.822865000	-0.723958000	-0.887330000
N	-1.297524000	-0.125201000	1.519983000
O	-1.393343000	-0.236216000	2.710106000
N	0.850607000	2.184793000	-0.138062000
H	3.520015000	-2.190371000	-0.248338000
H	3.270671000	-1.334516000	1.271472000
H	4.837452000	-0.030648000	-0.102472000
H	3.720059000	-0.035842000	-1.459944000
H	-3.215401000	1.133011000	-1.505452000
H	-3.721880000	0.788831000	0.059428000
H	3.332266000	1.344275000	1.242156000
H	3.514069000	2.132911000	-0.321057000
H	1.144632000	3.149946000	-0.120171000

Sum of electronic and zero-point Energies= -838.103810
Sum of electronic and thermal Energies= -838.090141
Sum of electronic and thermal Enthalpies= -838.089197
Sum of electronic and thermal Free Energies= -838.143913

Product, 3

Mo	1.015711000	0.004686000	0.013703000
O	0.411581000	-1.909455000	-0.346412000
O	0.384112000	1.986432000	-0.222251000
O	0.928764000	0.013604000	-2.311922000
O	2.852821000	0.774059000	-0.421267000
N	-0.903785000	-2.261630000	-0.166019000
N	-1.067512000	-0.013072000	0.017815000
N	2.983563000	-0.590960000	-0.429319000
C	-1.669354000	-1.248999000	0.011384000
C	-3.148927000	-1.324905000	0.201603000
C	-3.819100000	-0.080054000	-0.378862000
C	-3.203707000	1.196592000	0.194672000
C	-1.720981000	1.131794000	0.063594000
H	-3.367919000	-1.384405000	1.274188000
H	-3.533742000	-2.235259000	-0.258648000
H	-4.887240000	-0.095375000	-0.159064000
H	-3.712741000	-0.076907000	-1.467469000
H	-3.589145000	2.087393000	-0.303760000
H	-3.451157000	1.286690000	1.257901000
H	3.633497000	-0.926457000	0.274849000
H	3.201587000	-0.952048000	-1.354532000
H	0.666406000	0.866270000	-2.678167000
H	0.303858000	-0.639493000	-2.647165000
N	1.143537000	-0.066863000	1.733823000
O	1.194943000	-0.117510000	2.929093000
N	-0.937799000	2.177407000	-0.024865000
H	-1.254632000	3.134976000	-0.005969000

Sum of electronic and zero-point Energies= -914.476040
Sum of electronic and thermal Energies= -914.459427
Sum of electronic and thermal Enthalpies= -914.458483
Sum of electronic and thermal Free Energies= -914.519275

Table S7. Cartesian coordinates of the geometries corresponding to the terminal points of the intrinsic reaction coordinate (IRC) paths computed at the **PBE0/Def2-TZVP/PCM(water)** level. These endpoint structures correspond to geometries located near the potential-energy minima connected by the respective transition states (TS1 and TS2) and confirm the correct connectivity along each reaction path.

TS1 (IRC reverse endpoint → toward Im1⁻)

Mo	-1.09857400	-0.03972300	0.07979900
O	-0.50236200	-1.87443500	0.66538900
N	0.98914900	-0.16464300	-0.18546800
C	1.59427100	-1.30255100	0.23107000
N	0.83777700	-2.24318200	0.68870600
O	-0.46356000	1.70082900	-0.73937800
C	3.08674400	-1.40638400	0.16179800
N	0.88935500	1.93111700	-1.03913100
O	-2.48491000	0.29631100	1.08525700
C	3.62373200	-0.52009600	-0.96160300
N	-2.39987900	-0.16249300	-1.65410800
C	3.10606100	0.91023600	-0.82283900
C	1.61298500	0.91463100	-0.70212800
O	-1.20947200	-0.85710300	-1.80834300
N	-0.14961100	0.67615100	2.07404100
O	0.45215900	1.95152400	2.04589500
H	3.38352800	-2.44801100	0.02972100
H	3.51082000	-1.06963400	1.11570900
H	4.71548200	-0.52196100	-0.94600900
H	3.31595900	-0.92866200	-1.92965500
H	-2.47421800	0.60131400	-2.31957500
H	-3.20799100	-0.77421500	-1.71534600
H	3.52647600	1.35777700	0.08600700
H	3.41647200	1.53647300	-1.66066600
H	-0.91606100	0.72159600	2.74007200
H	0.55580300	0.03666100	2.43096800
H	0.30197500	2.24099700	1.13030700

TS1 (IRC forward endpoint → toward Im2)

Mo	-1.02770700	-0.00319900	0.11288900
O	-0.46666900	-1.81942600	0.62889500
N	1.00696100	-0.17525400	-0.24986200
C	1.62349400	-1.28526500	0.22480700
N	0.86277500	-2.19898300	0.72583300
O	-0.44463400	1.60781700	-0.85949000
C	3.11457300	-1.38309300	0.15972400
N	0.89267900	1.91971700	-1.07806700
O	-2.71692300	0.24185800	1.07944600
C	3.64334700	-0.52477900	-0.98846100
N	-2.38617100	-0.08415900	-1.50511600
C	3.12850100	0.90941500	-0.87879400
C	1.63592700	0.91336900	-0.75161900
O	-1.31714800	-0.87861700	-1.77129300
N	-0.36492300	0.61135300	2.00081000
O	0.41324600	1.81385300	2.08532800
H	3.41547000	-2.42632500	0.05464300
H	3.53555100	-1.01965600	1.10469000
H	4.73476500	-0.52583700	-0.97988600

H	3.32875000	-0.95591800	-1.94404600
H	-2.46214400	0.71262400	-2.13287200
H	-3.26281200	-0.58892400	-1.40997300
H	3.56069600	1.37969600	0.01208300
H	3.42913200	1.51309600	-1.73616200
H	-2.40894400	0.47873200	1.96729500
H	0.22694100	-0.10897000	2.41241300
H	0.98201600	1.83223300	1.30351300

TS2 (IRC reverse endpoint → toward Im2)

Mo	-0.72843900	-0.23285200	0.16981500
O	0.01080100	0.76844000	1.85804900
N	1.32285400	0.02171800	-0.08916000
C	1.94045700	0.90483400	0.77431100
N	1.25928800	1.30071300	1.79012900
O	-0.14155800	-1.57641600	-1.25616700
C	3.35140000	1.29926000	0.48603600
O	-3.94326400	0.91781500	-1.43411600
C	4.10477700	0.11006600	-0.11277200
N	-2.75126600	-0.75528000	-0.01043400
C	3.38832700	-0.43680500	-1.34762700
C	1.93669900	-0.60820900	-1.05434600
O	-2.38523000	-0.30798400	1.25613500
N	-1.17358700	1.28274500	-0.81983800
O	-0.52104900	2.56634200	-0.83495500
N	1.11374900	-1.37242600	-1.78441600
H	3.83144800	1.65737400	1.39737700
H	3.35948900	2.12621700	-0.23450600
H	-4.49360400	1.50312000	-0.90836600
H	-2.07938000	1.44565200	-1.28246000
H	5.11926900	0.40764000	-0.38276100
H	4.18953800	-0.68198700	0.63726000
H	-2.99573500	-1.74160300	0.04686700
H	-3.43055900	0.02267100	-0.64558000
H	3.50200900	0.25956200	-2.18670600
H	3.82542500	-1.38587000	-1.66757800
H	0.15916900	2.47687300	-0.15375900
H	1.46518200	-2.15319900	-2.32041100
O	-0.22985600	-2.25437800	1.42808500
H	0.51421200	-2.09195800	2.01832900
H	0.11797300	-2.79515400	0.70849600

TS2 (IRC forward endpoint → toward Im3)

Mo	-0.74813700	-0.19782800	0.17623800
O	-0.00657500	0.82746800	1.83269800
N	1.29528900	0.08836400	-0.10926500
C	1.92414900	0.95269200	0.76828200
N	1.24521300	1.33359500	1.79312100
O	-0.12669500	-1.62012900	-1.16824600
C	3.34677800	1.31771500	0.50395500
O	-3.84160400	1.28231100	-1.59048900
C	4.08870200	0.10908400	-0.06942600
N	-2.76663500	-0.86968800	0.14523100
C	3.38055100	-0.43621200	-1.30943400
C	1.92091500	-0.57464300	-1.04306500

O	-2.36290900	-0.36594000	1.37007200
N	-1.28476400	1.17739200	-0.88448800
O	-0.54528100	2.44753100	-0.96489600
N	1.10329800	-1.36099900	-1.77890800
H	3.81421700	1.67382000	1.42266300
H	3.38659900	2.13835500	-0.22263400
H	-4.23220300	2.03932700	-1.14673000
H	-2.84043700	1.34178100	-1.39653700
H	5.11303100	0.38514700	-0.32507300
H	4.14537100	-0.67657600	0.68990600
H	-3.04488100	-1.84194000	0.22396700
H	-3.48464600	-0.28299000	-0.28894200
H	3.52897700	0.24571800	-2.15567500
H	3.80125900	-1.39951800	-1.60966500
H	0.19117200	2.34480200	-0.34324100
H	1.50084200	-2.18936300	-2.20476300
O	0.00804600	-2.62584000	1.36683000
H	0.90236600	-2.49445600	1.69587600
H	0.09316000	-2.63094900	0.39441500

References

(1) G.M. Sheldrick. Crystal structure refinement with SHELXL. Acta Cryst. C71, , 3.
(2) C.B. Hübschle, G.M. Sheldrick, B. Dittrich. ShelXle: a Qt graphical user interface for SHELXL. J. Appl. Cryst. 44, **2011**, 1281.
(3) M. Nardelli. Modeling hydroxyl and water H atoms. J. Appl. Cryst. 32, **1999**, 563.
(4) L.G. Farrugia. WinGX and ORTEP for Windows: an update. J. Appl. Cryst. 45, **2012**, 849.
(5) A.L. Spek. Structure validation in chemical crystallography. Acta Cryst. D65, **2009**, 148.
(6) Diamond - Crystal and Molecular Structure Visualization, Crystal Impact - Dr. H. Putz & Dr. K. Brandenburg GbR, Kreuzherrenstr. 102, 53227 Bonn, Germany, https://www.crystalimpact.de/diamond.
(7) L.J. Barbour. X-Seed — A Software Tool for Supramolecular Crystallography. J. Supramol. Chem. 1, **2001**, 189.
(8) Gaussian 16, Revision C.01, Frisch, M. J.; Trucks, G. W.; Schlegel, H. B.; Scuseria, G. E.; Robb, M. A.; Cheeseman, J. R.; Scalmani, G.; Barone, V.; Petersson, G. A.; Nakatsuji, H.; Li, X.; Caricato, M.; Marenich, A. V.; Bloino, J.; Janesko, B. G.; Gomperts, R.; Mennucci, B.; Hratchian, H. P.; Ortiz, J. V.; Izmaylov, A. F.; Sonnenberg, J. L.; Williams-Young, D.; Ding, F.; Lipparini, F.; Egidi, F.; Goings, J.; Peng, B.; Petrone, A.; Henderson, T.; Ranasinghe, D.; Zakrzewski, V. G.; Gao, J.; Rega, N.; Zheng, G.; Liang, W.; Hada, M.; Ehara, M.; Toyota, K.; Fukuda, R.; Hasegawa, J.; Ishida, M.; Nakajima, T.; Honda, Y.; Kitao, O.; Nakai, H.; Vreven, T.; Throssell, K.; Montgomery, J. A., Jr.; Peralta, J. E.; Ogliaro, F.; Bearpark, M. J.; Heyd, J. J.; Brothers, E. N.; Kudin, K. N.; Staroverov, V. N.; Keith, T. A.; Kobayashi, R.; Normand, J.; Raghavachari, K.; Rendell, A. P.; Burant, J. C.; Iyengar, S. S.; Tomasi, J.; Cossi, M.; Millam, J. M.; Klene, M.; Adamo, C.; Cammi, R.; Ochterski, J. W.; Martin, R. L.; Morokuma, K.; Farkas, O.; Foresman, J. B.; Fox, D. J. Gaussian, Inc., Wallingford CT, 2016.
(9) Vetere, V.; Adamo, C; Maldivi, P., *Chem. Phys. Lett.*, **2000**, *325*, 99.
(10) Adamo, C; Barone, V., *Theor. Chem. Acc.*, **2000**, *105*, 169.
(11) Adamo, C.; Barone, V., *J. Chem. Phys.*, **1999**, *110*, 6158.
(12) Ernzerhof, M.; Scuseria, G. E., *J. Chem. Phys.*, **1999**, *110*, 5029.
(13) Adamo, C.; Scuseria, G. E.; Barone, V., *J. Chem. Phys.*, **1999**, *111*, 2889.
(14) Perdew, J. P.; Burke, K.; Ernzerhof, M., *Phys. Rev. Lett.*, **1996**, *77*, 3865.
(15) Weigend, F.; Ahlrichs, R. *Phys. Chem. Chem. Phys.* **2005**, *7*, 3297–3305.
(16) Tomasi, J.; Mennucci, B.; Cammi, R. *Chem. Rev.* **2005**, *105*, 2999–3094.
(17) Reed, A. E.; Curtiss, L. A.; Weinhold, F. *Chem. Rev.* **1988**, *88*, 899–926.
(18) Lu, T.; Chen, F. *J. Comput. Chem.* **2012**, *33*, 580–592.
(19) Martin, R. L. *J. Chem. Phys.* **2003**, *118*, 4775–4777.
